# Epoxide Stereochemistry Controls Regioselective Ketoreduction in Epoxyquinoid Biosynthesis

**DOI:** 10.1021/jacs.5c10778

**Published:** 2025-07-29

**Authors:** Szu-Yu Wang, Kuei-Wei Chiu, Ke-Li Lin, Hsin-Yu Wei, Yu-Rong Chen, Zhijay Tu, Yi-Tzu Lin, Chun-Hung Lin, Rong-Jie Chein, Hsiao-Ching Lin

**Affiliations:** † Institute of Biological Chemistry, 38017 Academia Sinica, Taipei 115201, Taiwan R.O.C; ‡ Institute of Biochemical Sciences, National Taiwan University, Taipei 106, Taiwan R.O.C; § Institute of Chemistry, Academia Sinica, Taipei 115201, Taiwan R.O.C; ∥ Department of Chemistry, National Taiwan University, Taipei 106, Taiwan R.O.C

## Abstract

Epoxyquinoids possess multiple contiguous chiral centers and reactive functional groups, offering valuable opportunities for drug discovery and chemical biology. Despite their structural diversity, the biosynthetic pathways of these species remain poorly understood. Here, we fully elucidate the biosynthesis of (−)-asperpentyn (**2**) through heterologous reconstitution in , chemical synthesis, chiral analysis, and biochemical assays. We identify critical pathway enzymes, including the FAD-binding monooxygenase AtyG, cupin domain-containing protein AtyE, and ketoreductases AtyD and AtyC. AtyE performs oxidation and stereoselective (2*R*,3*S*)-epoxidation, converting siccayne (**4**) to (2*R*,3*S*)-β-epoxyquinone (**6**). AtyD and AtyC control *S*- and *R*-stereochemistry, respectively, for ketoreductions at C-4 and C-1 positions, with the regioselectivity of AtyD influenced by substrate epoxide configuration. Specifically, AtyD regioselectively reduces the C4-keto group on **6** but cannot further reduce the C1-keto group on (+)-harveynone (**8**), whereas (2*S*,3*R*)-α-epoxyquinone (**10**) undergoes reduction at both C-1 and C-4 positions. Subsequent AtyC-mediated reduction installs opposite stereochemistry at C-1, yielding **2**. Kinetic analyses confirm the biosynthetic sequence from **6** to **8** (AtyD) and **8** to **2** (AtyC). Mutagenesis demonstrates that residue F97 in AtyD critically determines regioselectivity, while mutations at L137A and Q246A shift the regioselectivity. Four new epoxyquinols (**7**, **11**, **15,** and **16**) and four new epoxyhydroquinones (**9**, **12**, **14,** and **17**) were generated, enhancing mechanistic insights into AtyD and AtyC catalyses. Notably, an *in vitro* one-pot reaction using AtyD_F97A and AtyC successfully converted **10** to (+)-asperpentyn (**1**). This comprehensive characterization of stereochemistry control in **2** biosynthesis expands enzymatic tools for generating chemical complexity among epoxyquinoid natural products.

## Graphical abstract

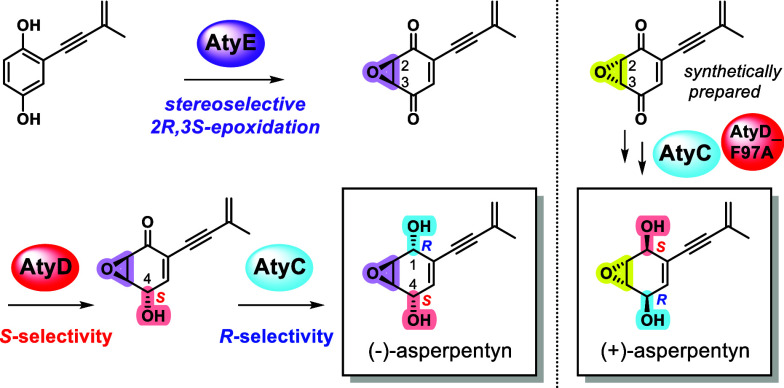

## Introduction

Epoxyquinoid natural products contain a characteristic fused ring system comprising an epoxy (oxirane) and a cyclohexenone moiety, and are classified as epoxycyclohexenones. These compounds are widespread across terrestrial and marine organisms, including fungi, bacteria, insects, plants, mollusks, and sponges.^1^ Structural diversity arises from variations in the epoxyquinone scaffold across different oxidation states (classes I–III) and additional functional group modifications (Figure 1A). Epoxyquinoids exhibit diverse bioactivities, such as antimicrobial (e.g., terreic acid,^2^ a covalent inhibitor of MurA), immunomodulatory (e.g., 4’-oxomacrophorin A^3^) antitumor (e.g., panepoxydone,^4^ NF-κB inhibitor), and anti-HIV (e.g., integrasone^5^) effects. Their biological reactivity stems from electrophilic quinone groups and strained epoxide rings, facilitating protein cross-linking interactions,^2,4,6^ thus underpinning their broad range of pharmacological activities.

**Figure 1:**
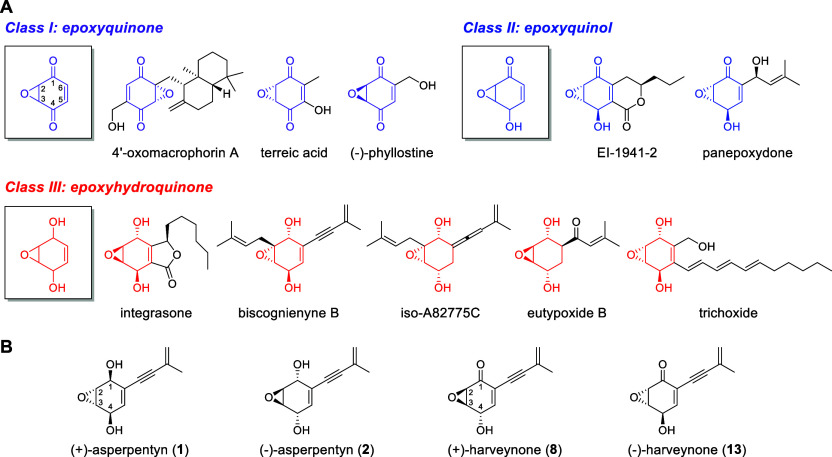
Structures of (A) epoxyquinoid classes (I–III) with representative natural products; (B) enantiomeric pairs of asperpentyn (**1** and **2**) and harveynone (**8** and **13**).

The highly oxygenated epoxyquinoid cores possess multiple contiguous chiral centers and reactive functionalities (Figure 1A). Remarkable stereochemical diversity arises from various permutations of hydroxyl and epoxide configurations observed in epoxyquinol (class II) and epoxyhydroquinone (class III) natural products.^7^ These structural features contribute significantly to their pharmacological potential and chemical biology relevance. Despite their extensive stereochemical variations, the biosynthetic pathways and enzymes responsible for epoxyquinoid formation remain poorly understood. Elucidating the enzymatic mechanisms controlling stereochemistry in epoxyquinoids is therefore essential to advance fundamental knowledge and enable practical applications in biosynthetic engineering.

Asperpentyn and harveynone are epoxy-containing, 1,3-enyne cyclohexanoid terpenoids classified into epoxyquinol and epoxyhydroquinone groups, respectively (Figure 1B). Both enantiomers have been isolated from fungi: (+)-asperpentyn (**1**) from *Aspergillus sp.* PSU-RSPG185,^8^ (−)-asperpentyn (**2**) from ,^9^ *sp.* PSU-MA69^10^ and ;^11^ (+)-harveynone (**8**) from the tea pathogen ,^12^ and (−)-harveynone (**13**) from .^13^ Their distinctive structural complexity has inspired many total syntheses, employing methods such as Pd-catalyzed Sonogashira and Stille couplings.^14^ Shared stereochemical patterns at the 4-hydroxyl and epoxy moieties between **1**/**13** and **2**/**8** suggest critical stereo- and regioselective enzymatic transformations during biosynthesis, yet the enzymatic mechanisms governing their stereochemical control remain unexplored.

We recently identified the *aty* gene cluster (GenBank accession number PQ683697.1) responsible for asperpentyn biosynthesis in *sp.* PSU-RSPG185 (Figure 3A).^15^ This cluster includes the phenylalanine ammonia-lyase AtyH, which converts l-phenylalanine into cinnamic acid, supplying benzenoid precursors such as *p*-hydroxybenzoic acid (Figure 2). UbiA prenyltransferase AtyB uniquely transfers a five-carbon prenyl chain to produce 4-hydroxy-3-prenylbenzoic acid (**3a**). Additionally, the P450 monooxygenase AtyI catalyzes a four-electron oxidation, converting **3a** into eutypinic acid (**3**), and installs the characteristic enyne functionality. The *aty* cluster further encodes four enzymes: AtyG (FAD-binding monooxygenase), AtyE (a cupin domain-containing protein), AtyC (a short-chain dehydrogenase/reductase, SDR), and AtyD (an aldehyde reductase), which are likely responsible for subsequent oxidative and reductive tailoring from intermediate **3** to **1**. However, the detailed enzymatic mechanisms governing epoxidation and ketoreduction, particularly their stereoselectivity and regioselectivity, remain unknown.

**Figure 2:**
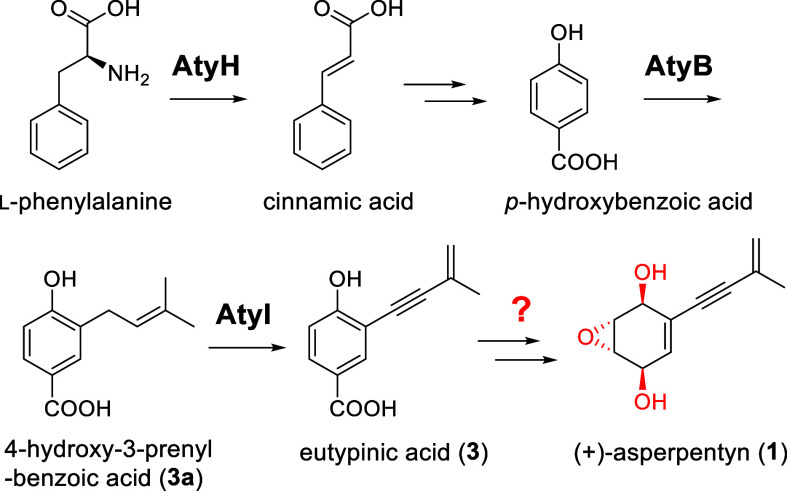
Upstream biosynthetic pathway leading to eutypinic acid (**3**) and the unknown redox tailoring steps toward **1** biosynthesis.

In this study, we fully elucidated the biosynthetic pathway of asperpentyn encoded by the *aty* gene cluster and determined its absolute stereochemistry as (−)-asperpentyn (**2**), which is distinct from the previously reported (+)-asperpentyn (**1**) isolated from *Aspergillus* sp. PSU-RSPG185. Additionally, we generated several novel epoxyquinol and epoxyhydroquinone compounds through enzymatic reductions catalyzed by AtyC, AtyD and its mutant variants. These products resulted from altered regio- and stereoselectivity when using precursors with opposite epoxide configurations.

## Results

### Biosynthetic Reconstitution in and Chemical Characterization of (−)-Asperpentyn (**2**)

To confirm the downstream biosynthetic genes responsible for asperpentyn production, intron-free *atyH*/*B*/*I*/*G*/*E*/*C*/*D* genes from sp. PSU-RSPG185 were cloned and coexpressed in NSAR1 (*niaD*–, *sC*–, Δ*argB*, *adeA*−). Coexpression yielded (−)-asperpentyn (**2**), eutypinic acid (**3**), and siccayne (**4**) (Figure 3B). Compound **2** was isolated from large-scale cultures and structurally identified as (−)-asperpentyn through detailed NMR analyses, consistent with previously reported spectra (Table S2 and Figure S23).^9–11^ The presence of the oxirane ring in **2** is supported by the characteristic ^13^C NMR signals at δ 53.0 (C-2) and δ 51.9 (C-3). The absolute stereochemistry was unequivocally confirmed by comparing chemically synthesized **1** and synthetic asperpentyn racemates using chiral column chromatography (Figure S1). These findings indicate that enzymes AtyG, AtyE, AtyC, and AtyD collectively orchestrate the biosynthetic formation of **2**.

**Figure 3:**
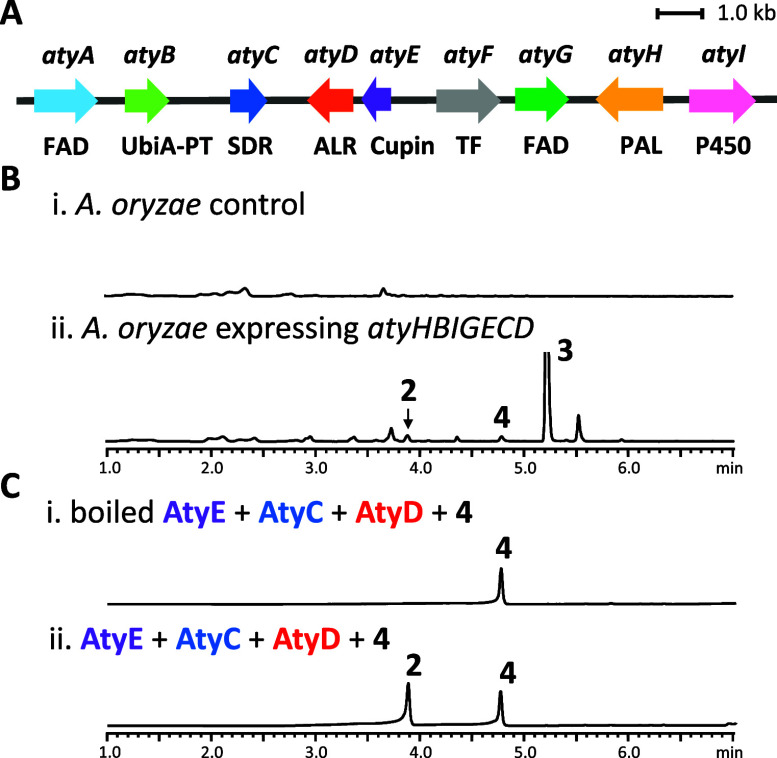
(A) The *aty* gene cluster from *sp.* PSU-RSPG185; liquid chromatography-diode array detection-mass spectrometry (LC-DAD-MS) analyses (λ = 257 nm) of (B) NSAR1 control and the transformant coexpressing *atyHBIGECD*, and (C) *in vitro* assays of AtyE, AtyC and AtyD with **4**.

### AtyG Catalyzes the Oxidative Decarboxylation of Eutypinic Acid (**3**) into Siccayne (**4**)

Interestingly, the absolute stereochemistry of **2** produced by expressing *atyH*/*B*/*I*/*G*/*E*/*C*/*D* differed from the previously reported **1** from sp. PSU-RSPG185.^8^ To verify the roles of the enzymes and the underlying biosynthetic logic, intron-free versions of *atyG*, *atyE*, *atyC* and *atyD* were cloned into expression vectors. The recombinant proteins were purified (Figure S2) and used for *in vitro* enzymatic assays.

AtyG, a FAD-binding monooxygenase sharing 38%/56% identity/similarity with VibMO1 (known for oxidative decarboxylation of **3a**) from the basidiomycete ,^16^ was expressed as a maltose-binding protein fusion (MBP-AtyG) in BL21 and purified. MBP-AtyG successfully converted **3** into **4** in the presence of NADPH (Figure 4A). Conversely, incubation with **3a** yielded no product, clearly distinguishing the substrate specificity of AtyG from VibMO1 (Figure 4B).

**Figure 4:**
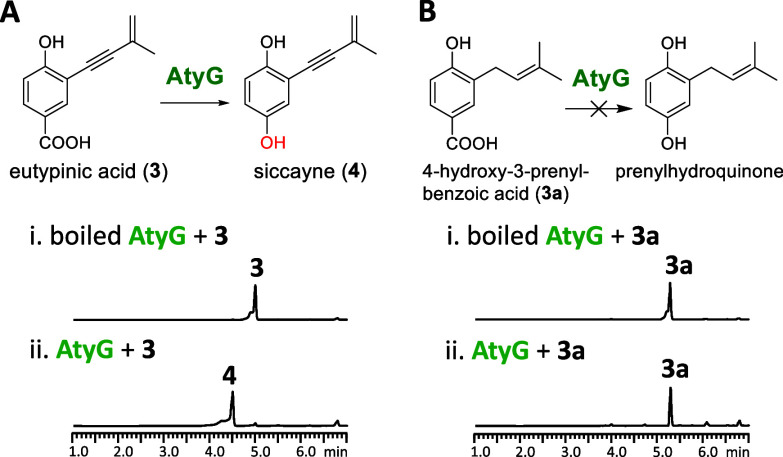
Functional verification of AtyG. LC-DAD-MS analyses (λ = 257 nm) of *in vitro* reactions of AtyG with NADPH and substrates (A) **3** and (B) **3a**.

### Identification of the Minimal Enzyme Set for (−)-Asperpentyn (2) Formation from Siccayne (**4**)

To identify the minimal enzyme set required for converting **4** into **2** and confirm its absolute stereochemistry, *in vitro* assays were performed using recombinant AtyE, AtyC, and AtyD proteins. Incubation of these purified enzymes with **4** in the presence of NADPH successfully yielded **2** (Figure 3C). The absolute stereochemistry of enzymatically synthesized **2** was validated by comparison to chemically synthesized asperpentyn racemates and **1** using chiral column chromatography (Figure S3A,B). These results establish that the minimal enzymatic machinery necessary for converting **4** to **2** comprises the cupin domain-containing protein AtyE, SDR AtyC, and aldehyde reductase AtyD.

### Functional Characterization of the Cupin Domain-Containing Epoxidase AtyE That Catalyzes Stereoselective Hydroquinone Oxidation and Epoxidation

AtyE, encoding a protein with a cupin domain (COG0662, mannose-6-phosphate isomerase, ManC) that belongs to the cupin superfamily. Sequence analysis reveals that the cupin domain of AtyE (coverage of 33% amino acid residues from 67 to 111) shares ∼ 30% identity with TcmJ^17^ from and MomA^18^ from IFO13271, both known quinone-forming monooxygenases involved in aromatic polyketide pathways such as tetracenomycin and mompain biosynthesis (Figure S4A). Within Ascomycota, AtyE displays moderate identity to BisC,^19^ IacF^7a^ and VirH^7b^ (65%, 64% and 50%, respectively), which are involved in epoxyquinone biosynthetic pathways of biscognienyne B, Iso-A82775C and trichoxide (Figure S4B). Although these enzymes have been associated with epoxyquinone production via heterologous coexpression or gene knockout studies, their exact biochemical roles have remained unclear due to a lack of detailed enzymatic characterization.

To investigate the catalytic function and stereoselectivity of AtyE, (2*R*,3*S*)-β-epoxyquinone (**6**) and (2*S*,3*R*)-α**-**epoxyquinone (**10**) were chemically synthesized (Supporting Information and Figure 5A). Analytical methods using gas chromatography–electron ionization mass spectrometry (GC-EI-MS) with chiral column separation were developed to distinguish both enantiomeric isomers (Figure 5B). Incubation of recombinant AtyE with **4** and NADPH produced exclusively **6**, confirming the stereoselective epoxidation activity of AtyE (Figures 5C and S5). To examine if 2-methylenyne-benzoquinone (**5**) is the potential intermediate in the conversion of **4** to **6**, **5** was chemically synthesized (Supporting Information). Incubation of **5** with AtyE and NADPH also yielded **6** (Figure 5D). Moreover, reactions combining **5** with AtyE, AtyC, and AtyD resulted in enhanced production of **2**, compared to that under the same conditions with **4** (Figure S6). These results establish AtyE as a ManC-type cupin protein catalyzing oxidation and stereospecific epoxidation of **4** via quinone intermediate **5**, forming **6**.

**Figure 5:**
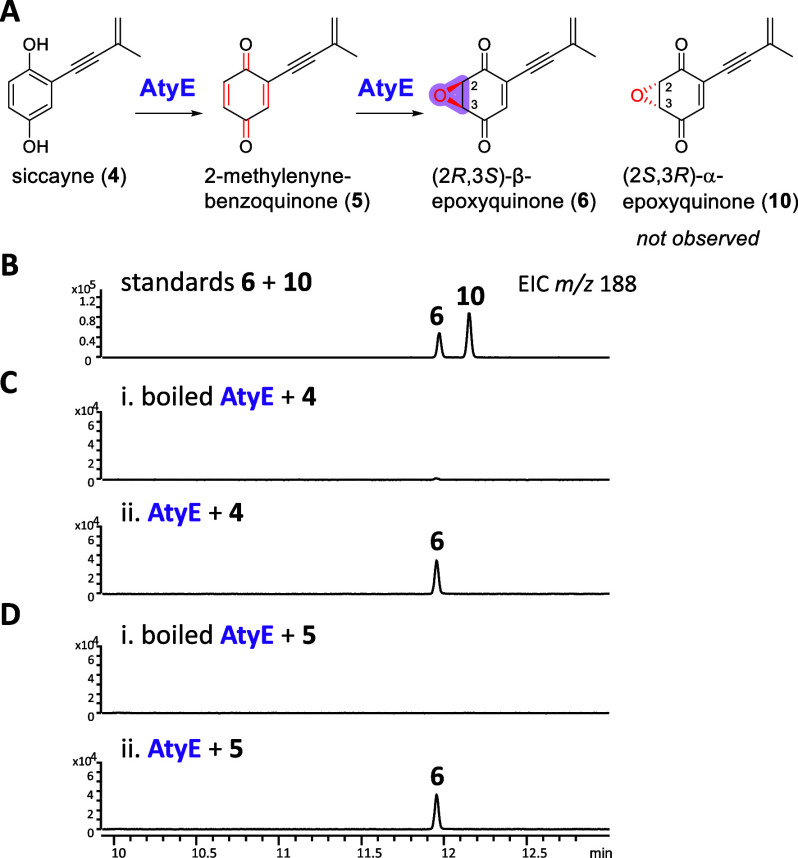
(A) Functional verification of AtyE. GC-EI-MS analyses of (B) synthetic standards **6** and **10**, and (C) *in vitro* assay of AtyE with substrates **4**, and (D) **5**.

### Biosynthetic Conversion of (2*R*,3*S*)-β-Epoxyquinone (**6**) to **2**

We first evaluated the catalytic functions of AtyC and AtyD using **6** as substrate. Co-incubation of **6** with NADPH and purified AtyC and AtyD yielded exclusively **2** (Figure 6E). The absolute stereochemistry of **2** was confirmed by comparison with chemically synthesized standards using chiral column chromatography (Figure S3C). To further verify the regio- and stereoselectivity of AtyC and AtyD, potential intermediates, 1*R*-hydroxy-asperpenone (**7)** and (+)-harveynone (**8**; 4*S*-hydroxyl at C-4), were synthesized (Figure 6A and Supporting Information).

**Figure 6:**
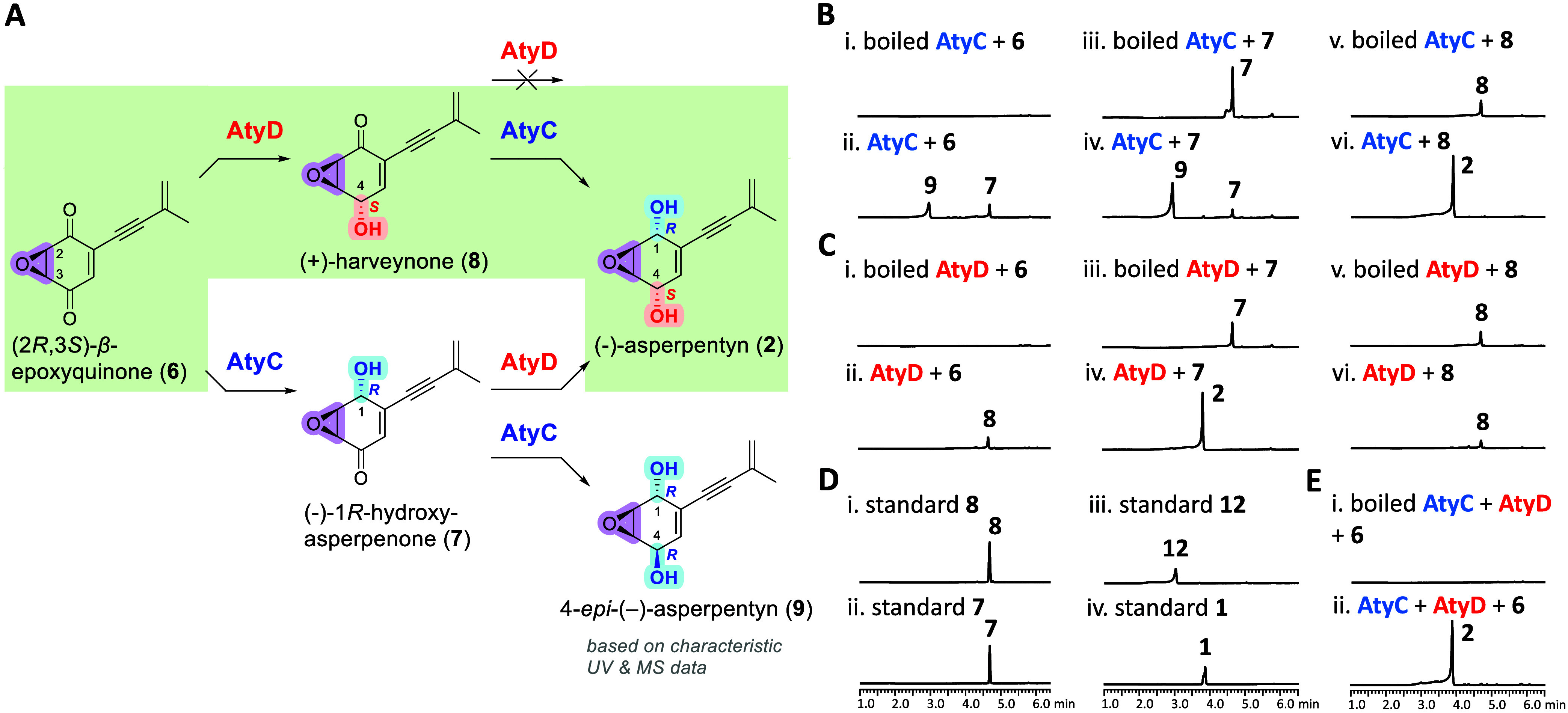
(A) Functional verification of AtyC and AtyD using **6** as substrate. The reactions highlighted in green are the main biosynthetic pathway of **2** verified by the kinetic analyses. LC-DAD-MS analyses (λ = 257 nm) of *in vitro* reactions: (B) AtyC with NADPH and substrates **6–8**; (C) AtyD with NADPH and substrates **6**–**8**; (D) synthetic standards **1**, **7**, **8**, and **12** (**1** and **12** are the enantiomer of **2** and **9**, respectively); and (E) combined incubation of AtyC and AtyD with NADPH and **6**.

#### AtyD Is a (S)-Ketoreductase and Shows Regioselectivity at C-4 on β-Epoxyquinoids

Incubation of **6** with AtyD and NADPH selectively produced **8** (*m*/*z* 189.1 [M–H]^−^) (Figure 6C, i and ii). Furthermore, incubating AtyD with **7** or **8** individually showed formation of **2** exclusively from intermediate **7**, while **8** remained unchanged (Figure 6C, iii–vi). These results confirm that AtyD functions as a regio- and stereoselective ketoreductase, selectively reducing the C-4 keto group of **6** and **7** to yield **8** and **2**, respectively, with precise *S*-stereochemistry at C-4.

#### The SDR AtyC Is a (R)-Ketoreductase at Both C-1 and C-4 on β-Epoxyquinoids

Incubating AtyC with NADPH and **6** yielded compounds **7** and **9** (*m*/*z* 175.1 [M + H–H_2_O]^+^) (Figure 6B,i and ii). Subsequent incubation of AtyC with **7** resulted in formation of **9** (Figure 6B, iii and iv). Compound **9** shared identical *m*/*z* with **2** (Figures S39B and S40C), yet had distinct retention times (*R*_t_ 2.9 min for **9** vs *R*_t_ 3.9 min for **2**), indicating a different stereochemistry at C-4. Thus, **9** was assigned as 4-*epi*-(−)-asperpentyn (Figure 6A), consistent with on the basis of UV absorption, mass spectra, and retention time identical to those of its enantiomeric standard, **12**, which was identified later using (2*S*,3*R*)-*α*-epoxyquinone (**10**) as substrate (Figure 7A). Furthermore, incubation of AtyC with **8** exclusively yielded **2** (Figure 6B, v and vi). These findings demonstrate that AtyC selectively reduces the C-1 keto groups of**6** and **8**, producing **7** and **2**, respectively, both bearing 1*R*-hydroxyl group. After establishing the 1*R*-hydroxyl functionality in **7**, AtyC subsequently catalyzes a second ketoreduction at C-4 with *R*-selectivity, yielding **9**.

**Figure 7:**
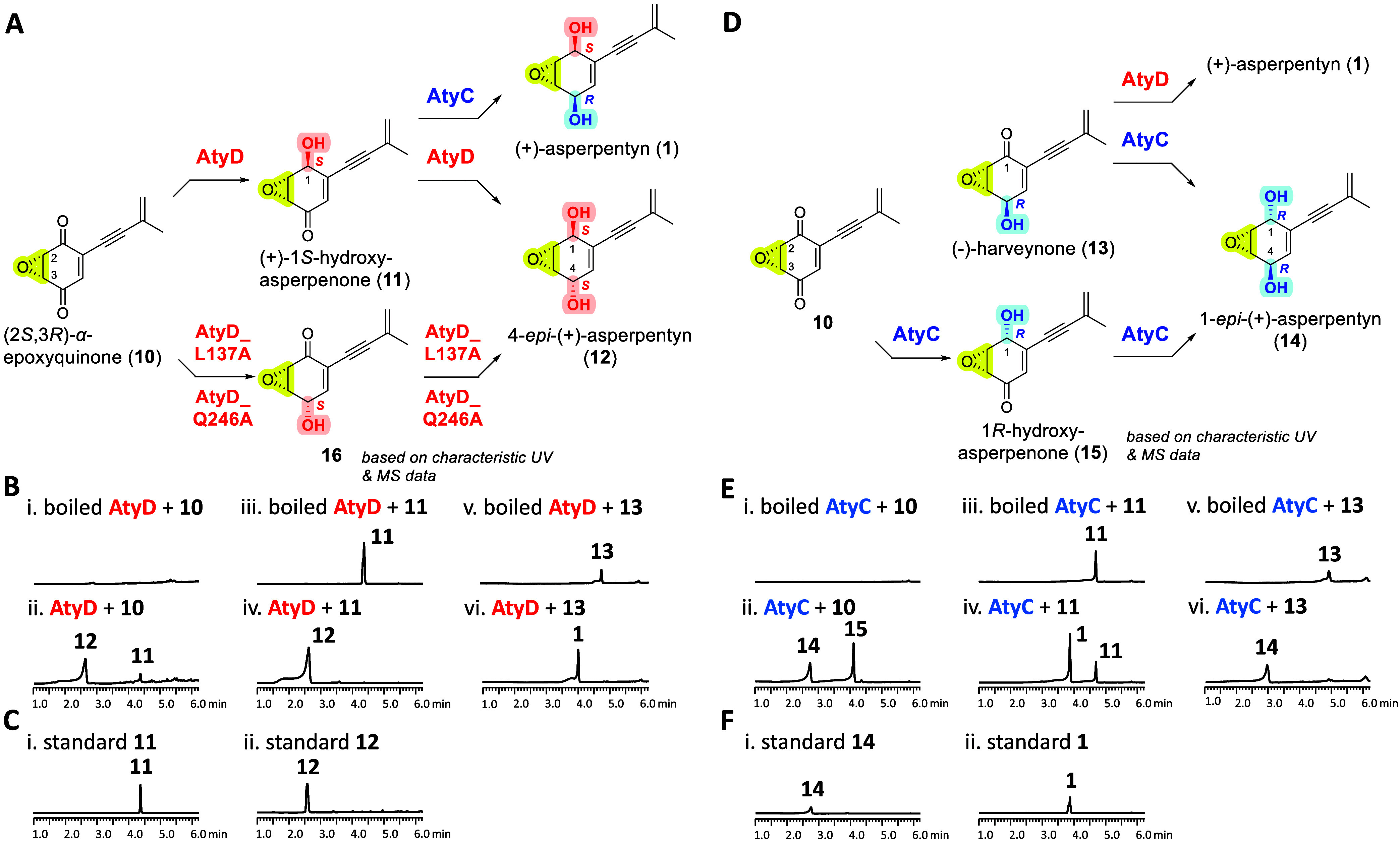
(A) Functional verification of AtyC and AtyD using **10** as substrate. LC-DAD-MS analyses (λ = 257 nm) of *in vitro* reactions: (B) AtyD with NADPH and substrates **10**–**11** and **13**; (C) synthetic standards **11** and **12**; (D) AtyC with NADPH and substrates **10**–**11** and **13**; and (E) synthetic standards **1** and **14**.

Collectively, these results illustrate opposite enantioselectivities of SDR AtyC (*R*-selective) and ketoreductase AtyD (*S*-selective). Additionally, while AtyD exhibits strict regioselectivity at C-4, AtyC catalyzes reductions at both C-1 and C-4, preferentially reducing the C-1 keto group (Figure 6A).

### Kinetic Analysis of AtyC and AtyD with **6**–**8**

To verify the preferred biosynthetic route from **6** to **2**, we performed steady-state kinetic studies with **6**–**8** using AtyC and AtyD (Table 1). For substrate **6**, the catalytic efficiency (*k*_cat_/*K*_m_) of AtyD was approximately 305-fold greater than AtyC, indicating a strong preference for converting **6** to **8** via AtyD. In the sequential conversion from **6** to **8** to **2**, the notably higher catalytic efficiency of AtyC with **8** (1.97 μM^–1^s^–1^) compared to AtyD with **6** (3.66 × 10^–3^ μM^–1^s^–1^) suggests the second step (**8** to **2**) proceeds rapidly. Conversely, with the minor intermediate **7** as a substrate, AtyD showed a 36-fold higher catalytic efficiency compared to AtyC, emphasizing that even this minor divergent route predominantly leads back to the production of **2** via AtyD. Thus, kinetic evidence supports a preferred biosynthetic sequence from **6** to **2** via intermediate **8**.

**Table 1:**
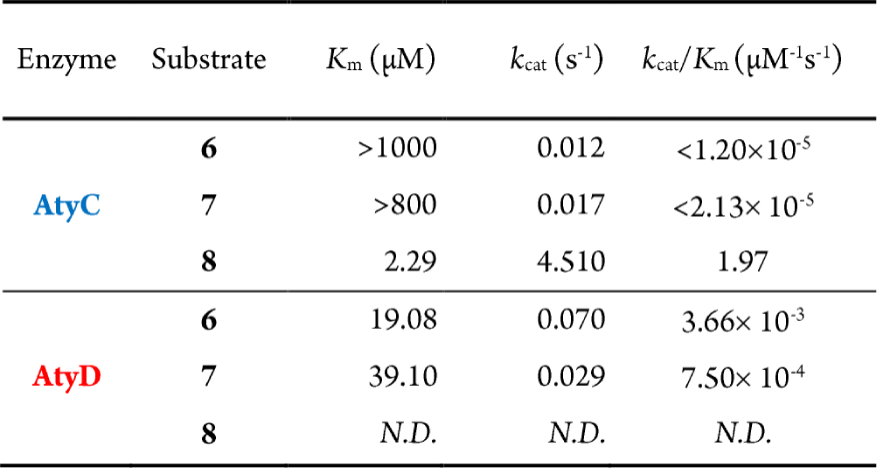
Steady-State Kinetic Parameters of **6**–**8** in Reactions Catalyzed by AtyC and AtyD^a^

a *N.D.*, not detected.

### Enzymatic Conversion of (2*S*,3*R*)-α-Epoxyquinone (**10**) by AtyD and AtyC

To investigate if (+)-asperpentyn (**1**) could be produced from **10**, and verify the enzymatic activities of AtyD and AtyC on **10**, chemically synthesized **10** was used as substrate for *in vitro* investigation.

#### Sequential C-1 and C-4 Ketoreduction of **10** by AtyD

Incubation of **10** with AtyD and NADPH yielded compounds **11** (*m*/*z* 191.0 [M + H]^+^) and **12** (*m*/*z* 175.1 [M + H–H_2_O]^+^) (Figure 7B, i and ii). Compound **12**, isolated from a larger-scale reaction, showed a characteristic ^1^H NMR signal for H-5 (δ 5.70, t, *J* = 2.2 Hz), differing from **1** (δ 5.88, dd, *J* = 4.9, 1.8 Hz), indicating a 4*S* configuration (Figure 7A, Table S5 and Figures S32–S36). Thus, **12** was designated as 4-*epi*-(+)-asperpentyn. Compound 11, matching the UV and mass spectral data and retention time of synthesized **7**, was determined to be its enantiomer. Therefore, **11** was chemically synthesized (Supporting Information), named as (+)-1*S*-hydroxy-asperpenone and used for *in vitro* reactions. Subsequent incubation of **11** with AtyD produced **12** (Figure 7B, iii and iv). Time-course assays confirmed intermediate **11** formation during conversion of **10** to **12** (Figure S9A), demonstrating sequential four-electron reduction by AtyD at C-1 ketone (to give 1*S*–OH) followed by C-4 ketone (to give 4*S*–OH).

#### Sequential C-1 and C-4 Ketoreduction of **10** by AtyC

Incubation of **10** with AtyC and NADPH produced compounds **15** (*m*/*z* 191.1 [M + H]^+^) and **14** (*m*/*z* 175.1 [M+H–H_2_O]^+^) (Figure 7E, i and ii). Compound **14** exhibited identical mass to **1** and **12** but distinct retention times (*R*_t_ 2.6, 2.8, and 3.9 min for **12**, **14** and **1**, respectively), indicating **14** is a diastereoisomer of **1** and **12**. Compound **14** was characterized as a new compound and named 1-*epi*-(+)-asperpentyn, by comparison to the physical data of synthesized **14** (Supporting Information). Compound **15** had a UV λ_max_ (306 nm) matching **11** (C-1 hydroxyl), distinct from **8** (C-4 hydroxyl; UV λ_max_ 219, 282 nm), and exhibited different retention from **11**, indicating an opposite stereochemistry (1*R*). Thus, compound **15** is proposed as a new intermediate, named 1*R*-hydroxy-asperpenone. Time-course analysis to monitor the reaction of AtyC with **10** indicated that there was a transient accumulation of **15,** accompanied by a concomitant increase in **14**, supporting **15** as an intermediate (Figure S9B). Additionally, incubation of chemically synthesized (−)-harveynone (**13**) with AtyC yielded **14**, confirming C-1 ketoreduction activity of AtyC (Figure 7E, v and vi). These results collectively show AtyC performs stereoselective ketoreductions at C-1 and C-4, both with *R*-selectivity, when acting on α-epoxyquinoids.

### One-Pot Reaction of AtyD and AtyC with (2*S*,3*R*)-α-Epoxyquinone (10) Gives 4-*Epi*-(+)-Asperpentyn (**12**)

To investigate if **1** could be produced from **10**, a combined enzymatic reaction using AtyD and AtyC was performed in the presence of NADPH. Surprisingly, this reaction primarily yielded **12**, along with minor amounts of **14**, rather than the expected product **1** (Figure 8A). These results indicated that the catalytic properties of AtyD and AtyC are significantly influenced by the epoxide configuration. Specifically, AtyD effectively catalyzed ketoreductions at both C-1 and C-4 of **10**, predominantly forming **12**, which competed with the regioselective C-4 (*S*)-ketoreduction by AtyC that can lead to **1** (Figure 7A). Additionally, AtyC preferentially catalyzed C-1 reduction of **10** to yield **15**, rather than initiating reduction at the C-4 position. Thus, the competing reactions between AtyD and AtyC for **10** were found to yield **12** (the major product) and **14** rather than **1**. Additionally, individual enzyme assays confirmed AtyD’s *S*-selectivity through conversion of **13** (4*R*–OH) into **1**, and AtyC’s *R*-selectivity via conversion of **11** (1*S*–OH) into **1** (Figure 7B, v and vi; 7E, iii and iv). Collectively, these findings confirm that the formation of **1** from **10** represents a very minor biosynthetic route.

**Figure 8:**
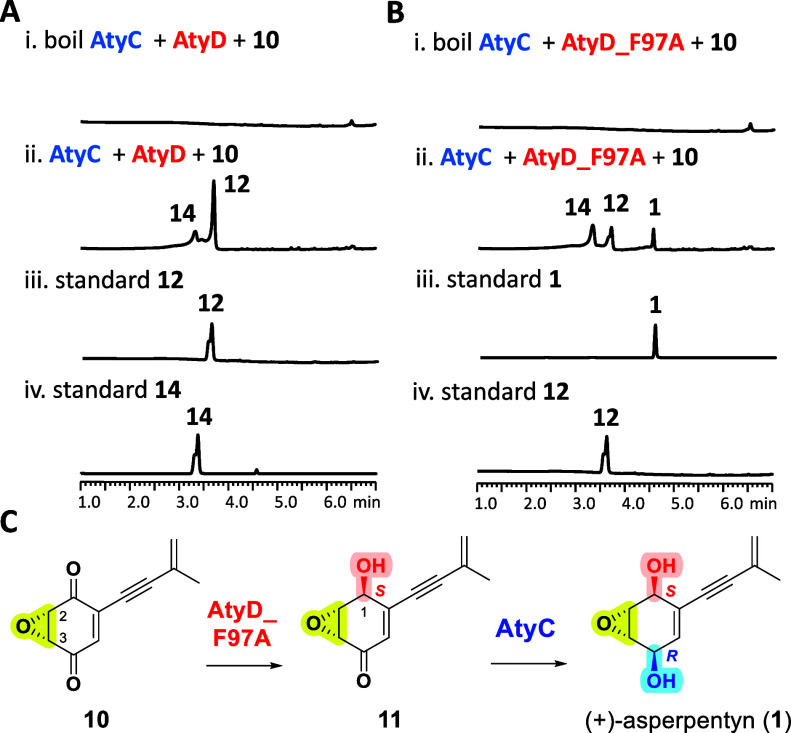
LC-DAD-MS analyses (λ = 257 nm) of *in vitro* reactions of (A) AtyC and AtyD with NADPH and **10**, and (B) AtyC and AtyD_F97A (ratio of 1:2) with NADPH and **10**; (C) one-pot reaction of AtyD_F97A and AtyC with **10** leading to the biosynthesis of **1**.

### Regioselectivity of AtyD Directed by β-Epoxide Controls Biosynthesis of (−)-Asperpentyn (**2**)

Our results demonstrate that the regioselectivity of AtyD is strongly influenced by the configuration of the epoxide, particularly the β-epoxide configuration. Specifically, AtyD selectively reduces the C-4 keto group of (2*R*,3*S*)-β-epoxyquinone (**6**) to yield **8** with strict *S*-selectivity and does not reduce the remaining C-1 keto group (Figure 10A). This regioselective property is essential for enabling subsequent reduction by AtyC at the C-1 position, which occurs with *R*-selectivity, leading directly to **2** characterized by its distinct 1*R*,4*S*-dihydroxyl stereochemistry. In contrast, AtyC alone exhibits broader activity, capable of reducing both the C-1 and C-4 keto groups, regardless of the α- or β-epoxide stereochemistry. Hence, the regioselectivity of AtyD, precisely controlled by β-epoxide configuration, critically defines the specific biosynthetic route toward **2**.

### Identification of Key Catalytic Residues and Mechanistic Insights into AtyD Function

To elucidate the catalytic mechanism and stereochemical control of AtyD, we employed a combination of protein modeling, molecular docking, and mutagenesis experiments. Structural modeling of AtyD by AlphaFold3 ^20^ and COFACTOR^21^-generated NADPH complexes identified three catalytic residues, Ser135, Tyr178, and Lys182, analogous to the aldehyde reductases from the red yeast (Figure S10).^22^

#### Key Residues Controlling AtyD Function on β-Epoxyquinoids

Molecular docking of **6** revealed positioning of Ser135 and Tyr178 near the C-4 keto group, with Lys182 suitably located for proton relay and NADPH positioned opposite for hydride transfer, supporting regioselective 4*S*-hydroxyl formation (Figures 10C and S11A). Additional active-site residues, Gln246 (near the C-1 keto group), Leu137/Asn208 (near the epoxide), and Phe97 (above the enyne group), were identified. Docking intermediates **7** and **8** exhibited similar binding orientations, which explains the selective inactivity of AtyD toward the C-1 keto group of **8,** due to the positioning of the 1-hydroxyl group in proximity to Y178 and NADPH, rather than the 1-keto group (Figure S11).

Mutagenesis validated these predictions: the catalytic triad triple mutant AtyD_S135C/Y178F/K182A completely abolished activity toward **6**, while single mutagenesis of S135C, Y178F and K182A attenuated **6** production (Figure S13). Residues L137 and Q246 influenced substrate binding, as indicated by reduced product formation in single mutants (L137A or Q246A), double (L137A/Q246A) and triple (L137A/Q246A/N208A) mutants. Notably, mutation F97A altered regioselectivity, generating novel product **17** (1-*epi*-(−)-asperpentyn, 1*S*,4*S*-diol configuration), an enantiomer of **14** (Figure 9B). Docking suggested an expanded active-site cavity in the F97A mutant, allowing altered substrate orientation, facilitating sequential reduction of both C-4 and C-1 keto groups in β-epoxide-containing compounds (Figures 10E and S14).

**Figure 9:**
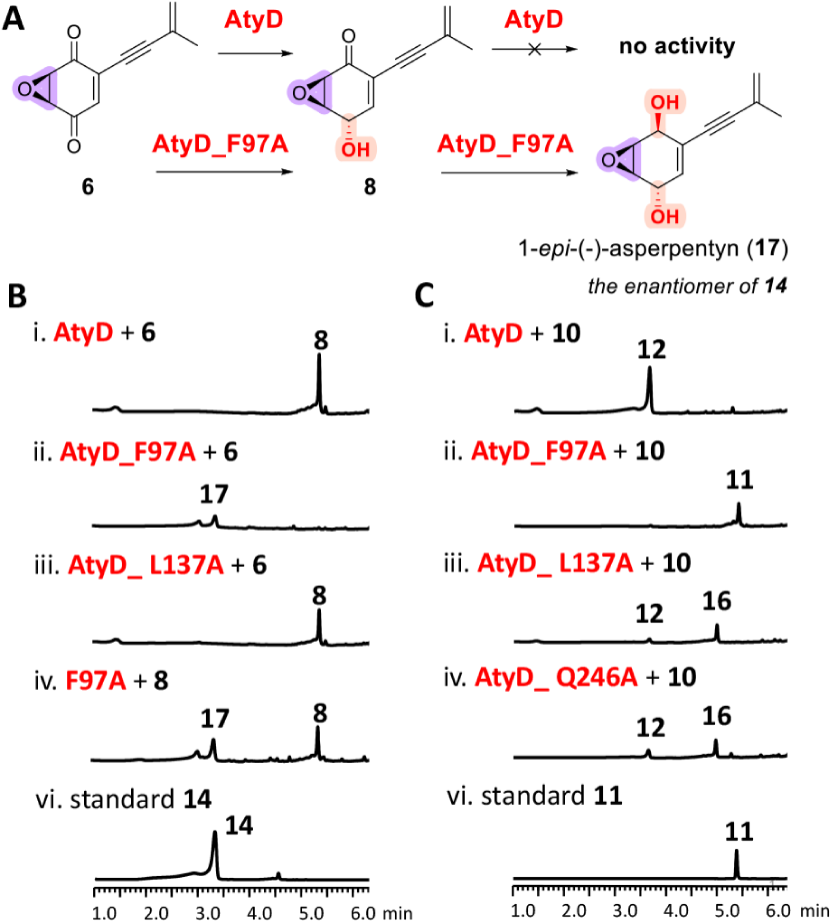
(A) Functional verification of AtyD mutants. LC-DAD-MS analyses (λ = 257 nm) of *in vitro* reactions of AtyD wild type and mutants with NADPH and (B) substrate **6** or (C) **10**.

**Figure 10:**
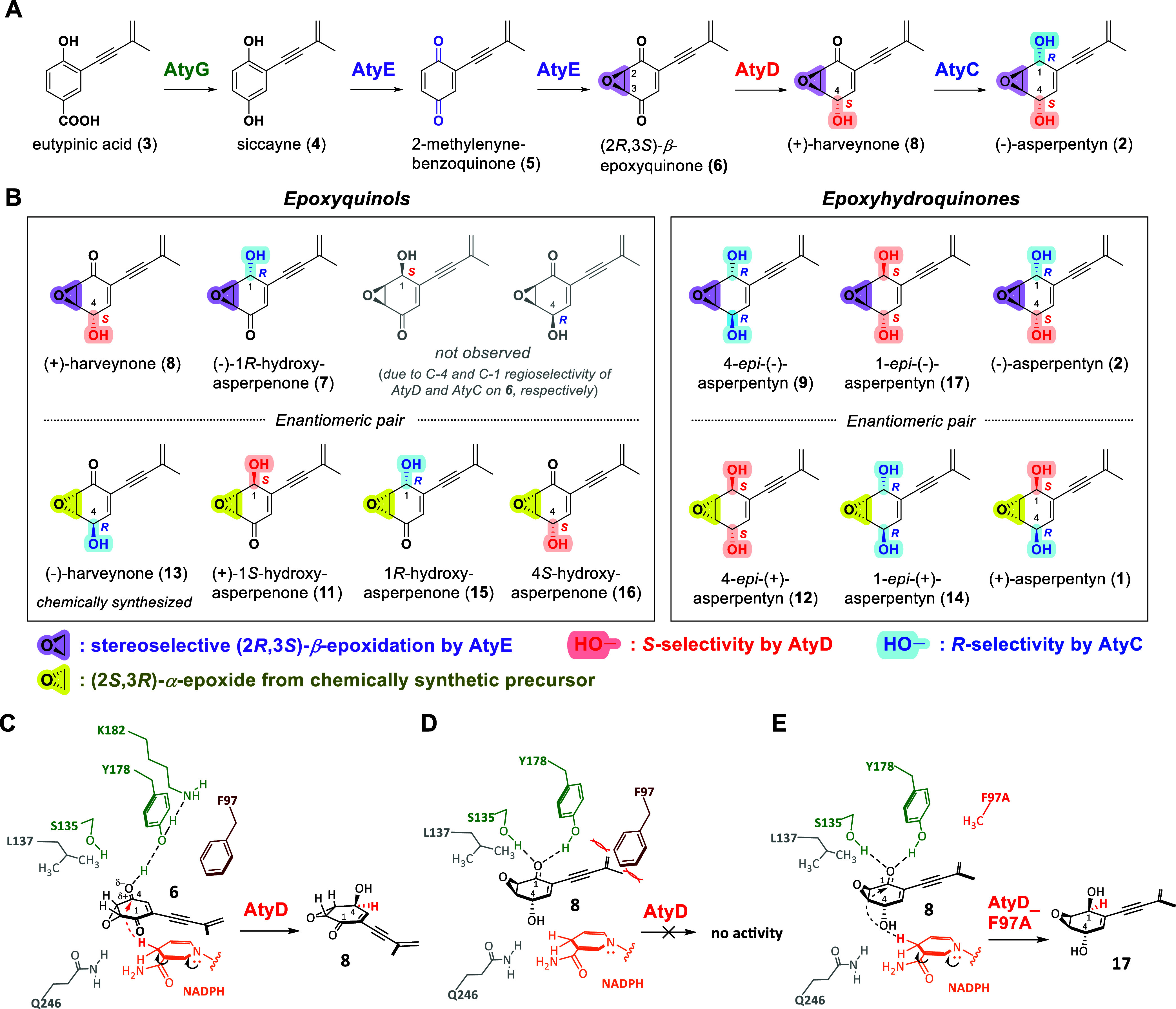
(A) The biosynthetic pathway of (−)-asperpentyn (**2**). (B) Structures of the enantiomeric pair of epoxyquinols and epoxyhydroquinones characterized in this study. (C–D) Proposed catalytic mechanisms of wild-type AtyD with substrates **6** and **8**, respectively. (E) AtyD_F97A mutant with **8**. The wild-type AtyD cannot accommodate **8** for C-1 ketoreduction due to steric hindrance caused by residue Phe97 interacting with the 1,3-enyne moiety of **8**. In contrast, the AtyD_F97A mutant permits substrate flipping, enabling the conversion of **6** into **8**, followed by subsequent *S*-selective C-1 ketoreduction to produce **17**.

#### Key Residues Controlling AtyD Function on α-Epoxyquinoids

Docking studies of α-epoxyquinoids **10** and (−)-harveynone (**13**) revealed a distinct binding mode from β-epoxyquinoid substrates (**6**–**8**), positioning the C-1 keto group near Tyr178 and NADPH, facilitating (*S*)-selective ketoreduction at C-1 to form **11** and **1**, respectively (Figure S15AB,). In this orientation, the epoxide faced away from Leu137 and toward Asn208, while the enyne group shifted near Phe97 and the C-4 keto positioned near Gln246. For **11**, docking showed that the epoxide group flipped toward Leu137, allowing the proximity of the C-4 keto group to Tyr178 and NADPH, which enabled the subsequent reduction to **12** (Figure S15C). Mutagenesis studies supported these structural insights. The catalytic residues triple mutant AtyD_S135C/Y178F/K182A abolished product formation from **10**, while the mutation N208A reduced product formation (Figure S16). Interestingly, the F97A mutant accumulated intermediate **11**, demonstrating slower subsequent reduction to **12 (** Figure 9C, ii). Time-course assays confirmed **11** accumulation before final conversion to **12** (Figure S17), indicating F97A mutation favored initial C-1 ketoreduction. Notably, the L137A and Q246A mutations significantly impacted regioselectivity. Single mutants (L137A or Q246A) accumulated a distinct intermediate, **16** (*m*/*z* 191.1 [M + H]^+^) together with **12 (** Figure 9C, iii-v). Compound **16** was characterized by identical UV λ_max_ (306 nm) but differing retention time compared to **13**, indicating altered stereochemistry at C-4 (4*R*–OH). Time-course analysis of AtyD_L137A confirmed that **16** is an intermediate in the formation of **12** from **10** (Figure S18). Interestingly, the double (L137A/Q246A and L137A/N208A) and triple (L137A/N208A/Q246A) mutants displayed a slower reaction rate to generate **16** only, while the double mutant N208A/Q246A produced a mixture of **16** and **12** (Figure S16). Docking comparisons indicated that the L137A mutation enlarged the active-site cavity, while the Q246A mutation disrupted hydrogen bonding with the C-4 keto group (Figure S19D,F). These structural changes likely permitted a flipped orientation of the epoxide, positioning the C-4 keto group in proximity to Tyr178 and NADPH, thereby facilitating its reduction to **16**. These findings establish Leu137 and Gln246 as critical residues governing substrate binding and regioselectivity toward α-epoxide substrates, with their mutation redirecting reduction from C-1 to C-4.

### Biosynthesis of (+)-Asperpentyn (1) from (2*S*,3*R*)-α-Epoxyquinone (**10**)

We observed that incubation of **10** with the AtyD_F97A mutant attenuated the activity of AtyD and led to the accumulation of **11** (Figure 9C). Subsequent enzymatic assays revealed that AtyC efficiently converted **11** to **1** (Figure 7E, iii and (iv). Based on these findings, a one-pot reaction combining the AtyD_F97A mutant and AtyC (at a 1:2 ratio) with **10** successfully yielded **1** (Figure 8B,C). These results not only demonstrate that the absolute stereochemistry of epoxyquinoid natural products can be controlled through site-directed mutation of catalytic residues in biosynthetic enzymes but also highlight how the presence of different enzyme homologues in nature can contribute to the stereochemical diversity observed in epoxyquinoid natural product biosynthesis.

## Discussion

In this study, we comprehensively identified the enzymatic pathway converting **3** to the structurally intricate **2**. Previous studies on enzymatic epoxidation described bacterial dioxygenases (DHAE I from LL-C10037 and DHAE II from MPP 3051) that catalyze opposite facial epoxidation on 2,5-dihydroxyacetanilide.^23^ Similarly, the basidiomycete mushroom cytochrome P450 monooxygenase PanH is known to epoxidize prenylhydroquinones and derivatives.^24^ In Ascomycota fungi, cupin-domain proteins AtD^25^ and PatJ^26^ (COG3837 superfamily) are involved in epoxyquinone formation in terreic acid and patulin biosynthesis. However, AtyE, characterized here, encodes a distinct cupin domain (COG0662, mannose-6-phosphate isomerase type), sharing low sequence identity (approximately 30%, and with 70% alignment coverage) with AtD and PatJ. Remarkably, AtyE performs stereospecific and regioselective (2*R*,3*S*)-β-epoxyquinone formation, differing from previously reported α-epoxide-forming enzymes.

The epoxycyclohexenol core of asperpentyn exhibits significant stereochemical diversity, as observed in other epoxycyclohexenol natural products. For instance, biscognienyne B^19^ and trichoxide^7b^ feature *trans*-diol (both *R*-configurations), whereas iso-A82775C^7a,15^ and eutypoxide B^27^ possess *cis*-diols with *R* and *S* configurations. Such stereochemical variability, including differing epoxide configurations, underscores extensive synthetic interest.^28^ Notably, characterization of the enzymes AtyC (*R*-selective ketoreductase) and AtyD (*S*-selective ketoreductase) suggests multiple stereoselective reductases govern the biosynthesis of epoxyhydroquinones. Indeed, the gene cluster (*iac*) of iso-A82775C encodes two AtyC homologues (IacC, XP_007830811, and IacC-1, XP_007834716) and one AtyD homologue (XP_007834717.1),^7a,15^ while the eutypoxide B cluster from *Eutypa lata* UCREL1 encodes AtyC (EMR66150.1) and AtyD (EMR66145.1) homologues (Figures S20 and S21).^15,29^ Detailed characterization of these homologous enzymes can significantly advance our understanding of stereoselectivity and regioselectivity, facilitating chemoenzymatic strategies for epoxyquinoid synthesis.

Additionally, this study expanded chemoenzymatic versatility, generating four novel epoxyhydroquinone (class III) derivatives (Figure 10B). AtyC catalyzed successive *R*-selective reductions on epoxyquinones **6** and **10**, yielding **9** (β-epoxide, 1*R*,4*R*-diol) and **14** (α-epoxide, 1*R*,4*R*-diol), respectively. Similarly, AtyD converted **10** into **12** (α-epoxide, 1*S*,4*S*-diol), whereas the AtyD-F97A mutant produced **17** (β-epoxide, 1*S*,4*S*-diol) from **6**. Notably, these novel products represent pairs of enantiomers (**9**/**12** and **14**/**17**), highlighting the biocatalytic potential of newly identified stereoselective enzymes for combinatorial biosynthesis and chemoenzymatic synthesis applications.

## Conclusions

We have elucidated the complete biosynthetic pathway for **2**, identifying key enzymatic roles of the FAD-binding monooxygenase AtyG, the cupin domain-containing protein AtyE, and the ketoreductases AtyD and AtyC. Specifically, AtyE catalyzes oxidation and stereoselective (2*R*,3*S*)-epoxidation of the hydroquinone moiety, converting **4** into (2*R*,3*S*)-β-epoxyquinone (**6**). Subsequent ketoreductions by AtyD and AtyC establish *S*- and *R*-configurations at C-4 and C-1, respectively. Notably, the regioselectivity of AtyD is critically dependent on substrate epoxide configuration; with **6**, AtyD exclusively reduces the C-4 keto group to produce intermediate **8**. Conversely, with (2*S*,3*R*)-α-epoxyquinone (**10**), AtyD reduces both C-1 and C-4, forming **12**. Kinetic analyses confirmed that the preferred biosynthetic pathway involves sequential conversion of **6** to **8** by AtyD, and subsequently **8** to **2** by AtyC. Structural and mutagenesis studies revealed the critical role of residue F97 in governing the regioselectivity of AtyD toward β-epoxyquinoid substrates. Substitutions such as L137A or Q246A notably shifted the reduction site from C-1 to C-4 in α-epoxyquinoid substrates. Notably, enzymatic synthesis using AtyD_F97A in combination with AtyC successfully converted **10** to **1**. In addition to elucidating key catalytic features, our study also led to the generation of several novel epoxyquinol (Class II) and epoxyhydroquinone (Class III) compounds. These findings not only provide mechanistic insights into epoxyquinoid biosynthesis but also expand the biocatalytic toolkit for the chemoenzymatic synthesis and structural diversification of complex epoxyquinoid natural products.

## Supplementary Material



## Notes

Experimental details and spectroscopic data (PDF)
