# Supplementary material for: Epoxide Stereochemistry Controls Regioselective Ketoreduction in Epoxyquinoid Biosynthesis
Source: J Am Chem Soc. 2025 Jul 29;147(32):29582–91. doi: 10.1021/jacs.5c10778 (PMC12356543; doi:10.1021/jacs.5c10778)
Supplement: Supplementary file 1 [file ja5c10778_si_001.pdf]

Supporting Information

Epoxide Stereochemistry Controls Regioselective Ketoreduction in Epoxyquinoid Biosynthesis

Szu-Yu Wang,[‡§#] Kuei-Wei Chiu,[‖#] Ke-Li Lin,[‡¶#] Hsin-Yu Wei,[‡§] Yu-Rong Chen,[‡] Zhijay Tu,[‡] Yi-Tzu Lin,[‡] Chun-Hung Lin,[‡§¶]* Rong-Jie Chein,[‖]* and Hsiao-Ching Lin[‡§]*

[‡]Institute of Biological Chemistry, Academia Sinica, Taipei 115, Taiwan R.O.C.

[§]Institute of Biochemical Sciences, National Taiwan University, Taipei 106, Taiwan R.O.C.

[‖]Institute of Chemistry, Academia Sinica, Taipei 115, Taiwan R.O.C.

[¶]Department of Chemistry, National Taiwan University, Taipei 106, Taiwan R.O.C.

[#]These authors contributed equally to this work.

Table of contents

1. Experimental Procedures 1
1.1 Strain sources and growth conditions 1
1.2 Molecular biology experiments 1
1.3 Plasmid cloning and construction 1
1.3.1 Construction of Plasmids for Heterologous Expression in A. oryzae NSAR1 1
1.3.2 Construction of pMAL-MBP-AtyG, pColdI-AtyE, pColdI-AtyC, pColdI-AtyD plasmids for expression in *E. coli* 2
1.3.3 Site-directed mutagenesis of AtyD 2
1.4 General Chemical analysis 2
1.5 Metabolites isolation from *Aspergillus oryzae* expressing *aty*HBIGECD 3
1.6 Overexpression and purification of MBP-tagged AtyG from *E. coli* 3
1.7 Overexpression and purification of His-tagged AtyE, AtyC and AtyD from *E. coli* 4
1.8 Overexpression and purification of His6-tagged AtyD mutants from *E. Coli* 4
1.9 *In vitro* assay of AtyG 5
1.10 2-methylenyne-benzoquinone (**5**) stability test 5
1.11 *In vitro* assay of AtyE 5
1.12 *In vitro* assay of AtyC 6
1.13 *In vitro* assay of AtyD 6
1.14 One pot reaction of AtyE, AtyC and AtyD 6
1.15 *In vitro* assay of AtyD mutants with compound **10** 7
1.16 One pot reaction of AtyC, AtyD and AtyC, AtyD_F97A with compound **10** 7
1.17 *In vitro* assay of AtyD mutants with compound **6** 7
1.18 *In vitro* assay of AtyD_F97A mutants with compound **8** 7
1.19 Time-course *in vitro* assay of the AtyD_F97A mutant with compound **10** 8
1.20 Time-course *in vitro* assay of the AtyD_L137A mutant with compound **10** 8
1.21 Protein structure prediction of AtyD 8
1.22 Molecular docking studies 8
1.23 pH-dependent enzyme activity assay 9
1.24 Kinetic assays of AtyC and AtyD 9
1.25 General information of chemical synthesis 10
1.26 Chemical synthesis of (−)-harveynone (**13**), (+)-asperpentyn (**1**), 1-*epi*-(+)-asperpentyn (**14**), (2*S*,3*R*)-α-epoxyquinone (**10**) 11
1.27 Chemical synthesis of siccayne (**4**) 19

1.28 Chemical synthesis of 2-methylenyne-benzoquinone (**5**) ... 20
1.29 Chemical synthesis of (−)-1*R*-hydroxy-asperpenone (**7**) ... 21
2. Supplementary Tables ... 30
Table S1. PCR primers used in this study. ... 30
Table S2. ^{1}H NMR (500 Hz) and ^{13}C NMR (125 Hz) spectroscopic data of (−)-asperpentyn (**2**) in DMSO-d_6. ... 32
Table S3. ^{1}H NMR (500 Hz) and ^{13}C NMR (125 Hz) spectroscopic data of eutypinic acid (3) in $CDCl_3$. ... 33
Table S4. ^{1}H NMR (500 Hz) and ^{13}C NMR (125 Hz) spectroscopic data of siccayne (**4**) in $CDCl_3$. 34
Table S5. ^{1}H NMR (500 Hz), ^{13}C NMR (125 Hz) and 2D NMR spectroscopic data of 4-*epi*-(+)-asperpentyn (**12**) in DMSO-d_6 ... 35
3. Supplementary Figures ... 38
Figure S1. Chiral HPLC analysis of asperpentyn. ... 38
Figure S2. SDS-PAGE of purified AtyG, AtyE, AtyC and AtyD proteins. ... 39
Figure S3. Chiral HPLC analysis of (−)-asperpentyn produced from *in vitro* enzymatic assays... 40
Figure S4. Protein sequence alignment of AtyE and its homologs. ... 41
Figure S5. GC-EI-MS chromatograms of *in vitro* assays with the cupin protein AtyE. ... 42
Figure S6. LC-DAD-MS analysis of *in vitro* enzymatic reactions of AtyE, AtyC, and AtyD. ... 43
Figure S7. LC-DAD-MS analysis of the stability of 2-methylenyne-benzoquinone (**5**) in different solvent environments. ... 44
Figure S8. Steady-state kinetic analysis of AtyC and AtyD. ... 45
Figure S9. LC-DAD-MS analysis of time-course *in vitro* assays of AtyD and AtyC with (2*S*,3*R*)-α-epoxyquinone (**10**). ... 46
Figure S10. AlphaFold3 structural model of AtyD and sequence alignment with homologous enzymes. ... 48
Figure S11. Molecular modeling and docking analysis of AtyD with **6**–**8**. ... 49
Figure S12. SDS-PAGE analysis of purified AtyD wild-type and mutants (protein size: 39.4 kDa). ... 50
Figure S13. Functional verification of AtyD mutants using substrate **6**. ... 51
Figure S14. Structural comparison of substrate binding pockets between AtyD and AtyD_F97A mutant with **8**. ... 52
Figure S15. Molecular modeling and docking analysis of AtyD with **10**, **11** and **13**. ... 53
Figure S16. Functional verification of AtyD mutants with **10**. ... 54
Figure S17. LC-DAD-MS chromatograms of the time-course reaction of AtyD_F97A with NADPH and substrate **10** (UV detection at λ = 257 nm). ... 55
Figure S18. LC-DAD-MS chromatograms of the time-course reaction of AtyD_L137A with

NADPH and substrate **10** (UV detection at λ = 257 nm). 56
Figure S19. Molecular modeling and docking analysis of AtyD_L137A, AtyD_Q246A, AtyD_L137A/Q246A, and AtyD_N208A/L137A with substrates **10** or **16**. 58
Figure S20. Examples of BGCs encoding epoxyquinoid natural products that contain AtyC or AtyD homologs. 59
Figure S21. Sequence alignment of AtyC with homologous proteins. 60
Figure S22. ^{1}H NMR spectrum ($CDCl_3$, 500 MHz) and ^{13}C NMR ($CDCl_3$, 125 MHz) of (+)-asperpentyn (**1**). 61
Figure S23. ^{1}H NMR spectrum (DMSO-d_6, 500 MHz) and ^{13}C NMR (DMSO-d_6, 125 MHz) of (-)-asperpentyn (**2**). 62
Figure S24. ^{1}H NMR spectrum ($CDCl_3$, 500 MHz) and ^{13}C NMR ($CDCl_3$, 125 MHz) of eutypinic acid (**3**). 63
Figure S25. ^{1}H NMR spectrum ($CDCl_3$, 500 MHz) and ^{13}C NMR ($CDCl_3$, 125 MHz) of siccayne (**4**). 64
Figure S26. ^{1}H NMR spectrum ($CDCl_3$, 500 MHz) and ^{13}C NMR ($CDCl_3$,125 MHz) of 2-methylenyne-benzoquinone (**5**). 65
Figure S27. ^{1}H NMR spectrum ($CDCl_3$, 500 MHz) of (2*R*,3*S*)-β-epoxyquinone (**6**). 66
Figure S28. ^{1}H NMR spectrum ($CDCl_3$, 500 MHz) and ^{13}C NMR ($CDCl_3$, 125 MHz) of (-)-1*R*-hydroxy-asperpenone (**7**). 67
Figure S29. ^{1}H NMR spectrum ($CDCl_3$, 500 MHz) of (+)-harveynone (**8**). 68
Figure S30. ^{1}H NMR spectrum ($CDCl_3$, 500 MHz) and ^{13}C NMR ($CDCl_3$, 125 MHz) of (2*S*,3*R*)-α-epoxyquinone (**10**). 69
Figure S31. ^{1}H NMR spectrum ($CDCl_3$, 500 MHz) of (+)-1*S*-hydroxy-asperpenone (**11**)............. 70
Figure S32. ^{1}H NMR spectrum (DMSO-d$_6$, 500 MHz) and ^{13}C NMR (DMSO-d$_6$, 125 MHz) of 4-*epi*-(+)-asperpentyn (**12**). 71
Figure S33. HSQC spectrum (DMSO-d_6, 500 MHz) of 4-*epi*-(+)-asperpentyn (**12**). 72
Figure S34. COSY spectrum (DMSO-d_6, 500 MHz) of 4-*epi*-(+)-asperpentyn (**12**)...................... 73
Figure S35. HMBC spectrum (DMSO-d_6, 500 MHz) of 4-*epi*-(+)-asperpentyn (**12**). 74
Figure S36. NOESY spectrum (DMSO-d_6, 500 MHz) of 4-*epi*-(+)-asperpentyn (**12**). 75
Figure S37. ^{1}H NMR spectrum ($CDCl_3$, 500 MHz) and ^{13}C NMR ($CDCl_3$, 125 MHz) of (-)-harveynone (**13**). 76
Figure S38. ^{1}H NMR spectrum ($CDCl_3$, 500 MHz) and ^{13}C NMR ($CDCl_3$, 125 MHz) of 1-*epi*-(+)-asperpenyn (**14**). 77
Figure S39. UV and MS spectra of **1**–**6**. 78
Figure S40. UV and MS spectra of **7**–**12**. 79
Figure S41. UV and MS spectra of **13**–**17**. 80

Figure S42. (+)-HRMS spectra of 4-*epi*-(+)-asperpentyn (**12**). 81
4. Supplementary references 82

1. Experimental Procedures

1.1 Strain sources and growth conditions

Aspergillus sp. PSU-RSPG185 strain was cultivated on Potato Dextrose Broth (PDB, HIMEDIA®) medium or PDA (PDB with 1.5% agar, HIMEDIA®) at 28°C and stored as 33% glycerol stock at -80°C.

Aspergillus oryzae NSAR1 was cultivated on DPY medium (2% glucose, 1% hipolypeptone, 0.5% KH_2PO_4, 0.05% $MgSO_4$, 0.5% yeast extract, 0.01% adenine) or DPY rich medium (replace 2% glucose into 2% dextrin) which was used for large scale compound isolation at 30°C, shaken at 120 rpm.

1.2 Molecular biology experiments

Polymerase chain reaction (PCR) was carried out by Q5® High Fidelity DNA polymerase sets (New England Biolabs). PCR products mixed with loading dye were separated on a 0.8% agarose gel in 1× TAE buffer (40 mM Tris, 40 mM acetate, 1 mM EDTA, pH 8.0). DNA bands were visualized and imaged using a Gel Doc™ EZ Imager (Bio-Rad). Target fragments were then purified, either directly from the gel or from the PCR mix, using the Zymoclean™ Gel DNA Recovery Kit (Zymo Research). DNA fragments were assembled by NEBuilder® HiFi DNA Assembly Master Mix (New England Biolabs). The assembled or ligated DNA products were transformed into DH10β (Invitrogen).

1.3 Plasmid cloning and construction

1.3.1 Construction of Plasmids for Heterologous Expression in *A. oryzae* NSAR1

The *atyH* and *atyB* fragments were amplified by PCR from genomic DNA of *Aspergillus sp.* PSU-RSPG185, while the intron-less *atyI*, *atyG*, *atyE*, *atyC*, and *atyD* coding sequences were obtained from its cDNA. To construct *atyHBGI*-pAdeA-M plasmid which harboring *atyH*, *atyB*, *atyI* and *atyG*, the pAdeA-M vector (with the *adeA* selection marker) was digested with NotI, PacI, AscI and XhoI. The four PCR fragments were then assembled into the linearized backbone using the NEBuilder® HiFi DNA Assembly Kit. To construct *atyECD*-pTAex3-M plasmid which harboring *atyE*, *atyC* and *atyD,* the pTAex3-M vector containing *argB* selection marker was digested with restriction enzymes NotI, PacI and XhoI. The three PCR fragments were then assembled into the linearized

backbone using the NEBuilder® HiFi DNA Assembly Kit.

1.3.2 Construction of pMAL-MBP-AtyG, pColdI-AtyE, pColdI-AtyC, pColdI-AtyD plasmids for expression in *E. coli*

Intron-less coding sequences of *atyG*, *atyE*, *atyC*, and *atyD* were amplified from *Aspergillus* sp. PSU-RSPG185 cDNA. To generate an MBP-tagged enzyme, the intron-less *atyG* fragment was assembled into the pMAL-MBP vector using NEBuilder® HiFi DNA Assembly. Similarly, the intron-less *atyE*, *atyC*, and *atyD* fragments were cloned into NdeI/SalI-digested pColdI by NEBuilder® HiFi DNA Assembly, yielding His_6-tagged pColdI-AtyE, pColdI-AtyC, and pColdI-AtyD constructs.

1.3.3 Site-directed mutagenesis of AtyD

Point mutations were introduced into *atyD* expression plasmids by site-directed mutagenesis using KOD Hot Start DNA Polymerase[1]. Mutation-specific primer pairs (Table S1) were designed to have matched melting temperatures and minimal secondary structure. PCR was carried out under the following cycling conditions: initial denaturation at 95 °C for 5 min; 30 cycles of 95 °C for 30 s, 60–65 °C for 45 s, and 68 °C for 1 min per kb; and a final extension at 68 °C for 10 min. The reaction products were then treated with DpnI to degrade the methylated parental plasmid template, and the mutagenized DNA was transformed into *E. coli* DH5α competent cells.

1.4 General Chemical analysis

The analytes were separated and detected on a Shimadzu LCMS-2020 system fitted with a Kinetex® Polar C18 column (2.1 × 100 mm, 2.6 µm, 100 Å) using electrospray ionization in both positive and negative modes. Chromatographic separation employed a linear gradient elution at 0.5 mL/min, ramping from 5% to 95% acetonitrile in water (each solvent containing 0.5% formic acid) over 10 minutes, followed by a 4-minute column wash at 95% acetonitrile/water. ^{1}H and ^{13}C NMR spectra were recorded on a Bruker Avance™ 500 MHz spectrometer equipped with an UltraShield™ Plus magnet and a 5 mm cryoprobe at the High Field NMR Center, Academia Sinica.

GC–MS analyses were carried out on an Agilent 7890B gas chromatograph coupled to a 5977B mass selective detector, with sample introduction by a PAL RSI 85 autosampler. Separation was achieved on a Cyclosil-B capillary column (30 m × 0.25 mm i.d., 0.25 µm film) using helium carrier gas at 1 mL/min. Samples were injected in splitless mode at 250 °C. The oven temperature program was as follows: initial hold at 80 °C for

2 min; ramp to 200 °C at 15 °C/min; final hold at 200 °C for 3 min. Electron impact ionization was performed at 70 eV.

1.5 Metabolites isolation from *A. oryzae* expressing *atyHBIGECD*

The *A. oryzae* transformant expressing the *atyHBIGECD* cluster was revived from glycerol stock and seed-cultured in 3 mL DPY medium at 30 °C and 120 rpm for 4 days. This seed culture was then used to inoculate 2 L of DPY, which was incubated under the same conditions for an additional 5 days. After fermentation, mycelia were harvested by vacuum filtration and extracted three times with 500 mL acetone. The combined filtrate was partitioned against an equal volume of ethyl acetate (3×500 mL), and the organic layer was concentrated under reduced pressure to yield a crude extract. This extract was fractionated by reversed-phase MPLC on a RediSep Gold® C18 column (50 g). Fraction 4, containing (–)-asperpentyn (**2**), was purified by semi-preparative HPLC on a Luna® C18 column (250 × 46 mm, 5 µm, 100 Å) using an isocratic 30% acetonitrile/70% water (0.05% formic acid) method over 30 min at 2.0 mL/min, to give 1 mg of **2**. A subsequent semi-preparative HPLC run on the same column with a 27–54% acetonitrile gradient (0.05% formic acid) over 50 min at 2.5 mL/min afforded 7 mg of eutypinic acid (**3**) and 2 mg of siccayne (**4**).

1.6 Overexpression and purification of MBP-tagged AtyG from *E. coli*

The plasmid encoding N-terminal MBP-tagged *atyG* was transformed into *E. coli* BL21(DE3). A single colony was used to inoculate 10 mL LB (35 µg/mL ampicillin), which was grown overnight at 37 °C and 250 rpm. This seed culture was then diluted into 1 L LB (35 µg/mL ampicillin) and incubated at 37 °C until the OD_{600} reached 0.4–0.6. Expression was induced with 0.1 mM IPTG, and incubation continued at 16 °C and 250 rpm for 18–20 hours. Cells were harvested by centrifugation (3,750 × g, 4 °C, 10 min) and resuspended in 50 mL MBP binding buffer (200 mM NaCl, 1 mM EDTA, 20 mM Tris–HCl, pH 7.4). The suspension was lysed by two passes through a high-pressure homogenizer (NanoLyzer N-2) at 18,000 psi. The lysate was clarified by centrifugation (20,000 rpm, 4 °C, 30 min) and filtered through a 0.22 µm membrane. The cleared lysate was applied to a 5 mL MBPTrap HP column (Cytiva) on an ÄKTA™ system. After washing with binding buffer to remove non-specific proteins, MBP-AtyG was eluted with binding buffer supplemented with 10 mM maltose. Finally, the eluted protein was concentrated

and buffer-exchanged into 50 mM Tris–HCl, 100 mM NaCl, 10% glycerol, pH 8.0 using an Amicon® Ultra-15 centrifugal filter unit.

1.7 Overexpression and purification of His-tagged AtyE, AtyC and AtyD from *E. coli*

The recombinant proteins AtyE, AtyC or AtyD encoded by pColdI-AtyE, pColdI-AtyD or pCold-AtyC, respectively, was expressed with N-terminal His-tag and transformed into *E. coli* BL21 (DE3) after DNA sequencing. The seed culture of BL21 was cultured in 10 mL LB broth with 35 µg/mL ampicillin at 37°C, shaken at 250 rpm overnight, and inoculated into 1 L LB broth with ampicillin. Until OD_{600} reached 0.4–0.6, isopropylthio-β-D-galactoside (IPTG, 0.1 mM as final concentration) was added into 1 L LB broth and then incubated for 18–20 hours at 16°C. The cell pellets were concentrated by centrifugation at 3750 rpm at 4°C for 10 minutes and resuspended in 40 mL lysis buffer (10 mM imidazole, 20 mM Tris-Base, 500 mM NaCl, pH 7.9), then used cell disruptor (NanoLyzer N-2 High-Pressure Homogenizer) with 18 kpsi to homogenize cell twice. The lysate was centrifugated at 20,000 rpm at 4°C for 30 minutes and filtrated by a 0.22 µm filter. The supernatant of lysate was incubated with an appropriate volume of Ni–NTA agarose resin at 4 °C with gentle stirring overnight. The resin–protein slurry was packed into a gravity-flow column and washed with buffer containing 10 mM imidazole (20 mM Tris–Base, 500 mM NaCl, pH 7.9) to remove non-specific binders. Bound protein was then step-eluted with buffers containing 20 mM, 50 mM, and 250 mM imidazole, respectively. Elution fractions were analyzed by SDS-PAGE to identify those containing the target protein. Finally, pooled fractions were concentrated and buffer-exchanged into 50 mM Tris–HCl, 100 mM NaCl, 10% glycerol, pH 8.0 using an Amicon® Ultra-15 centrifugal filter unit.

1.8 Overexpression and purification of His6-tagged AtyD mutants from *E. Coli*

The *E. coli BL21*(DE3) harboring pCold-AtyD mutant plasmid was inoculated to 1 L LB liquid medium with 100 µg/mL ampicillin. The cells were incubated at 37 °C with shaking at 170 rpm to an OD600 value of 0.6–0.8. Next, 375 µM isopropylthio-β-D-galactoside (IPTG) was added to induce protein expression, followed by incubation at 15 °C with shaking at 170 rpm for 20 hours. The bacteria were harvested by centrifugation (6,000 × g at 4 °C for 30 minutes) and the pellet was resuspended in 40 mL lysis buffer (250 mM NaCl, 20 mM Tris-HCl, 5 % glycerol, pH 8.0) and lysed twice with a cell disruptor

(NanoLyzer N2) at 18 kpsi. The bacteria lysate was centrifuged (20,000 × g for 30 minutes) at 4 °C to remove cellular debris and inclusion body.

The recombinant proteins were purified by gravity-flow column chromatography with 2.0 mL Ni Sepharose 6 Fast Flow histidine-tagged protein purification resin (Cytiva). Briefly, all soluble proteins were passed through a 0.2 µm filter first and then applied to the column. Ten to twenty column volumes of washing buffer (250 mM NaCl, 20 mM Tris-HCl, 10 % glycerol, 10 mM to 50 mM imidazole, pH 8.0) was used to remove the non-binding proteins. Then, the his-tagged proteins were eluted with ten column volumes of elution buffer (washing buffer with 300 mM imidazole). The purified proteins were concentrated to 1.0-2.0 mL using an Amicon Ultra-15 Centrifugal Filter Unit and were analyzed by SDS-PAGE. Then, the purified proteins were quantified using a Nanodrop, and stored at –80°C until further use.

1.9 *In vitro* assay of AtyG

In vitro assays (50 µL) contained 100 mM phosphate buffer (pH 7.5), 1 mM NADPH, 250 µM substrate [eutypinic acid (**3**) or 4-hydroxy-3-prenylbenzoic acid (**3a**)], and 10 µM purified AtyG. Reactions were incubated at room temperature for 2 hours, with heat-inactivated enzyme as a negative control. After incubation, each reaction was quenched and extracted three times with 50 µL ethyl acetate. The combined organic layers were evaporated to dryness, re-dissolved in methanol, and analyzed by LC–DAD–MS.

1.10 2-methylenyne-benzoquinone (5) stability test

To evaluate the stability of 2-methylenyne-benzoquinone (**5**), a stock solution was prepared using tip to dip a little sample and dissolved in DMSO. The 1 µL of the DMSO stock solution was directly diluted with 99 µL of methanol or 90% acetonitrile. The prepared samples were then subjected with 10 µL to LC-DAD-MS analysis.

1.11 *In vitro* assay of AtyE

In vitro assay of AtyE was carried out with a 200 µL reaction, containing 100 mM phosphate buffer (pH 7.5), 1 mM NADPH, 10 µM divalent metal pool ($ZnSO_4 \cdot 7H_2O$, $NiSO_4 \cdot 6H_2O$, $CuSO_4 \cdot 5H_2O$, $FeSO_4 \cdot 7H_2O$, $CaCl_2$, $MnCl_2$, $MgCl_2$), 500 µM siccayne (**4**) or 1 mM 2-methylenyne-benzoquinone (**5**) as substrate, and 10 µM purified AtyE. The negative control was treated with boiling enzyme. After 15 min of incubation at room temperature, the reaction mixture was quenched by adding 200 µL ethyl acetate and

extracted three times. The ethyl acetate extracts was evaporated and dissolved in methanol followed by GC-MS analysis.

1.12 *In vitro* assay of AtyC

The composition in the assay contained 100 mM phosphate buffer (pH 7.5), 1 mM NADPH, 1 mM (2*R*,3*S*)-β-epoxyquinone (**6**), 1 mM (2*S*,3*R*)-α-epoxyquinone (**10**), 250 µM (-)-1*R*-hydroxy-asperpenone (**7**), 250 µM (+)-harveynone (**8**), 250 µM (+)-1*S*-hydroxy-asperpenone (**11**), 250 µM (-)-harveynone (**13**) as substrate, and 10 µM purified AtyC. The negative control was treated with boiling enzyme. After 15 minutes incubation at room temperature, the reaction was quenched and extracted with 50 µL ethyl acetate three times. For the time course analysis, the reaction was incubation at room temperature for 2.5, 5, 7.5, 10 and 12.5 minutes, and quenched and extracted with ethyl acetate. The mixture was evaporated and dissolved in methanol followed by analysis with LC-DAD-MS.

1.13 *In vitro* assay of AtyD

In vitro assay of AtyD was carried out with a 50 µL reaction, containing 100 mM phosphate buffer (pH 7.5), 1 mM NADPH, 1 mM (2*R*,3*S*)-β-epoxyquinone (**6**), 1 mM (2*S*,3*R*)-α-epoxyquinone (**10**), 250 µM (-)-1*R*-hydroxy-asperpenone (**7**), 250 µM (+)-harveynone (**8**), 250 µM (+)-1*S*-hydroxy-asperpenone (**11**), 250 µM (-)-harveynone (**13**) as substrate, and 10 µM purified AtyD. The negative control was treated with boiling enzyme. After 15 minutes of incubation at room temperature, the reaction mixture was quenched by adding 50 µL ethyl acetate and extracted three times. For the time course analysis, the reaction was incubation at room temperature for 2.5, 5, 7.5, 10 and 12.5 minutes, and quenched and extracted with ethyl acetate. The mixture was evaporated and dissolved in methanol followed by analysis with LC-DAD-MS.

1.14 One pot reaction of AtyE, AtyC and AtyD

The one-pot reaction contained 100 mM phosphate buffer (pH 7.5), 1 mM NADPH, 10 µM divalent metal pool ($ZnSO_4·7H_2O$, $NiSO_4·6H_2O$, $CuSO_4·5H_2O$, $CoCl_2·5H_2O$, $FeSO_4·7H_2O$, $CaCl_2$, $MnCl_2$, $MgCl_2$), 250 µM siccayne (**4**) or 2-methylenyne-benzoquinone (**5**) as substrate, and 10 µM purified AtyE, AtyC and AtyD. The negative control was treated with boiling enzymes. After 1 hour of incubation at room temperature, the reaction mixture was quenched by adding 50 µL ethyl acetate and extracted three times.

The ethyl acetate layer was evaporated and dissolved in methanol followed by analysis with LC-DAD-MS.

1.15 *In vitro* assay of AtyD mutants with compound 10

The enzymatic reactions of wild-type AtyD and its mutants were performed in a 25 µL reaction mixture containing 10 µM enzyme, 1 mM (2*S*,3*R*)-α-epoxyquinone (**10**), 1 mM NADPH and 100 mM phosphate buffer (pH 7.5). The reactions were incubated at room temperature for 5 min. The reaction mixture was quenched by adding 50 µL ethyl acetate and extracted three times. The ethyl acetate layer was collected and dried using a speed vacuum concentrator. The dried samples were dissolved in 20 µL methanol followed by analysis with LC-DAD-MS.

1.16 One pot reaction of AtyC, AtyD and AtyC, AtyD_F97A with compound 10

The one-pot assay was carried out with a 50 µL reaction, containing 100 mM phosphate buffer (pH 7.5), 1 mM NADPH, 1 mM (2*S*,3*R*)-α-epoxyquinone (**10**) as substrate, and 30 µM purified AtyC, 10 µM AtyD, or 10 µM AtyD_F97A. The negative control was treated with boiling enzymes. After 15 min of incubation at room temperature, the reaction mixture was quenched by adding 50 µL ethyl acetate and extracted three times. The ethyl acetate layer was evaporated and dissolved in methanol followed by analysis with LC-DAD-MS.

1.17 *In vitro* assay of AtyD mutants with compound 6

The enzymatic reactions of wild-type AtyD and its mutants were performed in a 12.5 µL reaction mixture containing 10 µM enzyme, 1 mM (2*R*,3*S*)-β-epoxyquinone (**6**), 1 mM NADPH and 100 mM phosphate buffer (pH 7.5). The reactions were incubated at room temperature for 2.5 min. The reaction mixture was quenched by adding 30 µL ethyl acetate and extracted three times. The ethyl acetate layer was collected and dried using a speed vacuum concentrator. The dried samples were dissolved in 20 µL methanol followed by analysis with LC-DAD-MS.

1.18 *In vitro* assay of AtyD_F97A mutants with compound 8

The enzymatic reactions of AtyD_F97A was performed in a 12.5 µL reaction mixture containing 10 µM enzyme, 1 mM (+)-harveynone (**8**), 1 mM NADPH and 100 mM phosphate buffer (pH 7.5). The reactions were incubated at room temperature for 10

min. The reaction mixture was quenched by adding 30 µL ethyl acetate and extracted three times. The ethyl acetate layer was collected and dried using a speed vacuum concentrator. The dried samples were dissolved in 20 µL methanol followed by analysis with LC-DAD-MS.

1.19 Time-course *in vitro* assay of the AtyD_F97A mutant with compound 10

The enzymatic reactions of the AtyD_F97A mutant were performed in a 25 µL reaction mixture containing 10 µM enzyme, 1 mM (2*S*,3*R*)-α-epoxyquinone (**10**), 1 mM NADPH and 100 mM phosphate buffer (pH 7.5). A boiled enzyme was used as a negative control. The reactions were incubated at room temperature for 2.5, 5, 10, 15, 30, 60 and 90 min, followed by extraction with 50 µL ethyl acetate for two times. The supernatant was collected and dried using a speed vacuum concentrator. The dried samples were dissolved in 20 µL methanol followed by analysis with LC-DAD-MS.

1.20 Time-course *in vitro* assay of the AtyD_L137A mutant with compound 10

The enzymatic reactions of AtyD_L137A mutant were performed in a 25 µL reaction mixture containing 10 µM enzyme, 1 mM (2*S*,3*R*)-α-epoxyquinone (**10**), 1 mM NADPH and 100 mM phosphate buffer (pH 7.5). A boiled enzyme was used as a negative control. The reactions were incubated at room temperature for 2.5, 5, 10, 15, 30, 60 and 90 min, followed by extraction with 50 µL ethyl acetate for two times. The ethyl acetate layer was collected and dried using a speed vacuum concentrator. The dried samples were dissolved in 20 µL methanol (MeOH) followed by analysis with LC-DAD-MS.

1.21 Protein structure prediction of AtyD

The predicted structure of AtyD was generated by AlphaFold3[2] which was selected based on the prediction with the highest confidence (rank_001; ipTM = 0.97; pTM = 0.95). Structural alignment by T-Coffee (multiple sequence alignment server) revealed that the predicted structure shares similarity to other known aldehyde reductase structures in Protein Data Bank such as SsCR (PDB ID: 1Y1P), Gre2 (PDB ID: 4PVD), and KpADH (PDB ID: 5Z2X) with identity of 22%, 21%, and 20%, respectively[3].

1.22 Molecular docking studies

Substrates **6**, **7**, **8**, **10**, **11** and **16** were generated using Chem3D 20.1.1 (PerkinElmer Chemoffice), and their energy were minimized to a minimum RMS gradient of 0.100 with

Molecular Mechanics (MM2). The above ligand substrates were manually docked into the active site of the AtyD protein model by Molecular Operating Environment (MOE) 2009.10. The complex structure with the best combination of low binding energy and favorable orientation was selected. The protein structures were analyzed and the figures were prepared by PyMOL.

1.23 pH-dependent enzyme activity assay

To determine the pH dependence of AtyC and AtyD, enzyme activity was measured across a pH range of 2.0 to 9.0. A Britton–Robinson buffer system (composed of 20 mM citric acid, 20 mM sodium phosphate, and 20 mM boric acid) was employed to cover the full pH range. The pH of each buffer was adjusted using 12 N NaOH. Reactions were performed at 28 °C in a total volume of 100 µL containing 100 µM NADPH, 200 µM substrate and an appropriate concentration of purified enzyme. Compound **6** was used as the substrate for AtyD, and compound **8** was used for AtyC. Enzyme activity was monitored by measuring the decrease in NADPH fluorescence (excitation at 360 nm, emission at 460 nm), and the initial velocity at each pH was normalized to the highest activity. The bar charts were generated by using GraphPad Prism (version 6). All measurements were performed in at least triplicate.

1.24 Kinetic assays of AtyC and AtyD

The kinetic parameters of AtyC and AtyD were determined by monitoring the consumption of NADPH through a decrease in fluorescence (excitation at 360 nm, emission at 460 nm), using a spectrophotometer (Microplate Reader M1000 Pro, Tecan). Reactions were carried out in black 96-well plates (Nunc 96F Nontreated Black Microwell, Thermo Scientific) with a total volume of 100 µL per well. The reaction mixture contained the substrate, 100 µM NADPH, 100 mM MES buffer (pH 5.5), and enzyme. The optimal enzyme concentration was determined by monitoring the initial reaction rate under the conditions: 100 µM NADPH, 200 µM substrate, and a serial dilution of the enzyme (0–10 µM) in 100 mM MES buffer (pH 5.5). The enzyme concentration that resulted in a linear increase in fluorescence signal during the initial phase of the reaction was selected for subsequent kinetic measurements.

For kinetic assays, the enzyme was pre-incubated with NADPH (100 µM) in the assay buffer at 28 °C for 5 min, followed by the addition of serial dilutions of the

substrate (0–500 μM). Fluorescence was recorded every 10 seconds over a 30-minute period. Kinetic constants were calculated using nonlinear regression (Michaelis–Menten equation) in GraphPad Prism (version 6). All measurements were performed in at least triplicate.

1.25 General information of chemical synthesis

All reactions using air-/moisture-sensitive reagents were performed in a flamed-dried apparatus, under an atmosphere of dry nitrogen, and standard syringe-septa techniques were followed. Solvents were freshly dried and purified by conventional methods before use. The progress of all the reactions was monitored by TLC using TLC glass plates precoated with silica gel 60 F254 (Merck). The visualization of TLC was done with UV, $KMnO_4$ stain, and ammonium molybdate stain. Column chromatography was performed on silica gel Geduran Si 60 (Merck). Optical rotation values were measured with a Jasco P-2000 polarimeter. FTIR spectra were recorded with a Thermo Nicolet iS-5 FT-IR spectrophotometer. ^{1}H and ^{13}C NMR spectra were recorded with Bruker AV-III 400 MHz, AV-400 MHz, and AV-500 MHz spectrometers, and chemical shifts were measured in δ (ppm) with residue solvent peaks as internal standards ($CDCl_3$, δ 7.26 ppm in ^{1}H NMR, δ 77.00 ppm in ^{13}C NMR). Coupling constants (*J*) are reported in hertz, and splitting abbreviations used were as follows: s, singlet; d, doublet; t, triplet; m, multiplet. HR FAB and HR EI mass spectra were recorded on a JMS-700 double-focusing mass spectrometer (JEOL, Tokyo, Japan) with a resolution of 8000 (3000) (5% valley definition). Melting points were recorded on a Buchi M-565 apparatus. The enantioselectivity of UV active products was determined by Agilent HPLC using Daicel chiral columns (Chiralpak AS, Chiralcel OJ). Single-crystal X-ray diffraction was measured in Bruker D8 VENTURE.

1.26 Chemical synthesis of (−)-harveynone (13)[4], (+)-asperpentyn (1), 1-*epi*-(+)-asperpentyn (14), (2*S*,3*R*)-α-epoxyquinone (10)

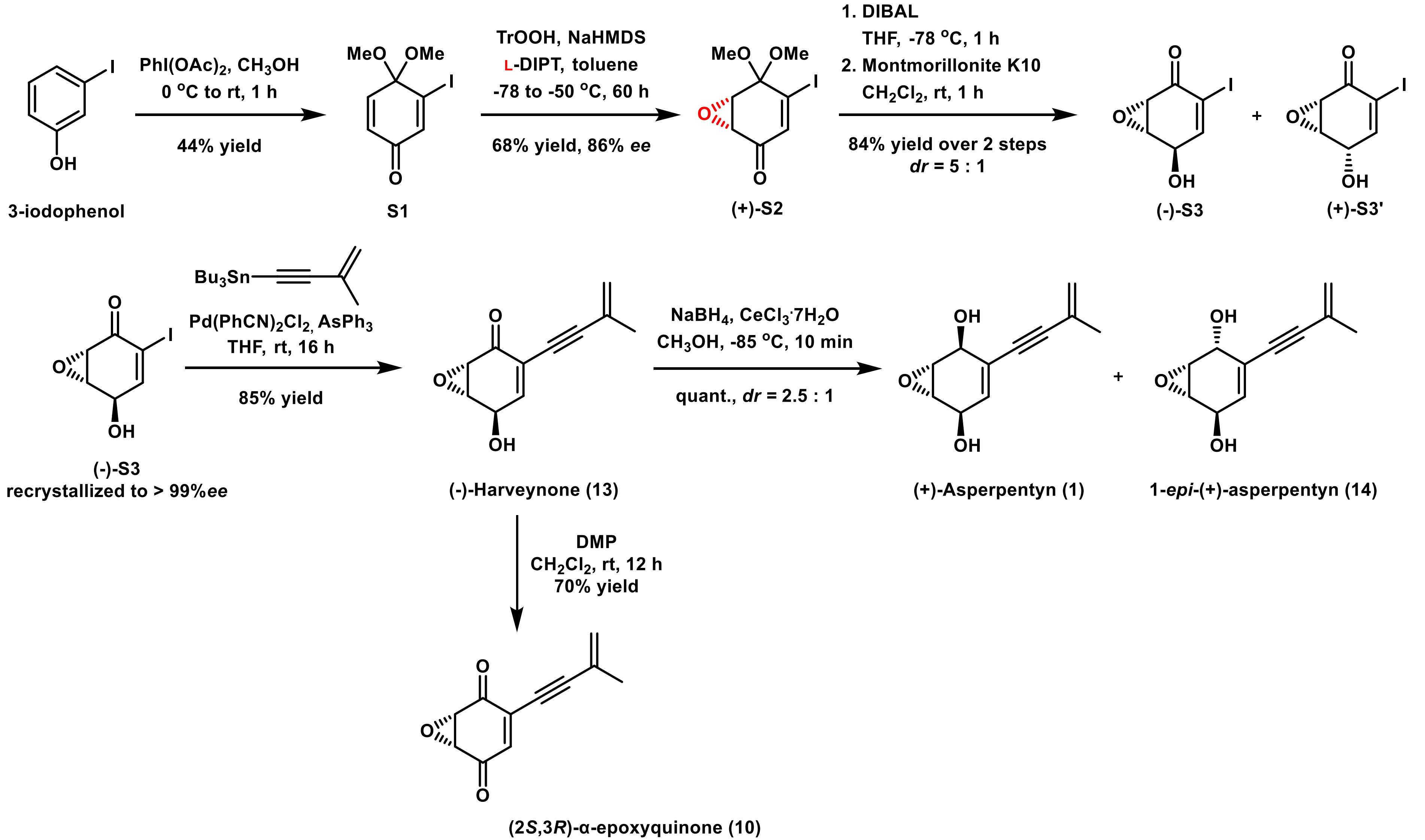

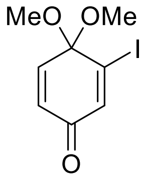

S1

To a stirred solution of 3-iodophenol (1.6 g, 7.3 mmol, 1.0 equiv.) in freshly distilled methanol (36 mL, 0.2 M to 3-iodophenol) at 0°C was added $PhI(OAc)_2$ (5.5 g, 17.1 mmol, 2.3 equiv.) in one portion. The ice bath was removed and the resultant was stirred at room temperature for 1 hour. After completion, the reaction mixture was passed through a plug of silica gel and eluted with EtOAc (30 mL). The filtrate was concentrated in vacuo, and the crude material was purified by flash column chromatography on silica gel (Et_2O/*n*-hexane, 3/7) to afford compound **S1** (900 mg, 44% yield) as an orange semi-solid. **^{1}H NMR (500 MHz, $CDCl_3$):** δ = 7.22 (d, *J* = 2.0 Hz, 1H), 6.96 (d, *J* = 10.2 Hz, 1H), 6.52 (dd, *J* = 10.4, 2.1 Hz, 1H), 3.26 (s, 6H). **^{13}C NMR (101 MHz, $CDCl_3$):** δ = 181.6, 143.4, 142.9, 132.0, 129.3, 94.5, 51.2.

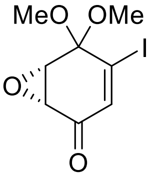

(+)-S2

To a freshly prepared solution of trityl hydroperoxide in toluene[5] (5.0 mL, 3.6 mmol, 5.0 equiv., ca. 0.72M) in a schlenk tube at -78°C was added NaHMDS (1.6 mL, 3.2 mmol, 4.5 equiv., 2.0 M solution in THF) dropwise. The transparent yellow solution was stirred at -78°C for 30 min. L-DIPT (184 mg, 0.79 mmol, 1.1 equiv.) in toluene (0.9 mL) was added dropwise at -78°C and then stirred at this temperature for 1 hour. **S1** (200 mg, 0.71 mmol, 1.0 equiv.) in toluene (1.7 mL) was added dropwise and the orange reaction mixture was warmed to -50°C and stirred at this temperature for 60 hours. The reaction was quenched with water (10 mL) and extracted with EtOAc (10 mL× 3). The combined organic layers were washed with brine, dried over anhydrous Na_2SO_4, filtered, and concentrated in vacuo. The crude material was purified by flash column chromatography on silica gel (EtOAc/*n*-hexane, 1/9) to afford compound **(+)-S2** (144 mg, 68% yield) as a yellowish oil. **^{1}H NMR (500 MHz, $CDCl_3$):** δ = 6.95 (d, *J* = 1.9 Hz, 1H), 3.88 (d, *J* = 4.0 Hz, 1H), 3.61 (s, 3H), 3.54 (dd, *J* = 4.0, 2.0 Hz, 1H), 3.45 (s, 3H). **^{13}C NMR (126 MHz, $CDCl_3$):** δ

= 189.4, 139.6, 124.7, 94.8, 52.4, 52.0, 51.4, 51.2. **FTIR (neat):** 3052, 2941, 2836, 1692, 1680, 1595, 1459, 1275, 1129, 1060 cm^{-1}. **HRMS (FAB) m/z:** $[M\text{-}OMe]^+$ Calcd for $C_7H_6IO_3$ 264.9356; Found $[M\text{-}OMe]^+$ 264.9360. ***ee***: 85%, determined by HPLC analysis (Chiralpak AS column, flow rate = 1.0 mL/min, 10% IPA/*n*-hexane, abs = 254.8 nm), $t_{r(minor)}$ = 18.04min, $t_{r(major)}$ = 24.56 min. $[\alpha]_D^{26}$ = +154.4 (c 1.0, $CHCl_3$, 85% *ee*).

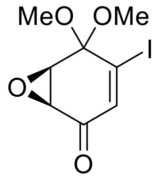

(-)-S2

The reaction was performed using trityl hydroperoxide solution (7.5 mL, 5.4 mmol, 5.0 equiv.), NaHMDS (2.4 mL, 4.8 mmol, 4.5 equiv.), D-DIPT (276 mg, 1.18 mmol, 1.1 equiv.) in toluene (1.5 mL) and compound **S1** (300 mg, 1.07 mmol, 1.0 equiv.) in toluene (2.5 mL). Compound **(−)-S2** was obtained (218 mg, 67% yield) as a pale-yellow oil. ***ee*:** 76%, determined by HPLC analysis (Chiralpak AS column, flow rate = 1.0 mL/min, 10% IPA/*n*-hexane, abs = 254.8 nm), $t_{r(major)}$ = 15.88min, $t_{r(minor)}$ = 22.36min. $[\alpha]_D^{26}$ = -125.9 (c 0.66, $CHCl_3$, 76% *ee*).

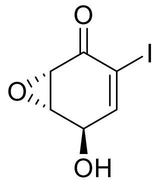

(-)-S3

To a stirred solution of compound **(+)-S2** (353 mg, 1.22 mmol, 1.0 equiv., 80% *ee*) in anhydrous THF (12 mL, 0.1 M to compound **(+)-S2**) at -78°C was added DIBAL (1.8 mL, 1.83 mmol, 1.5 equiv., 1.0 M solution in toluene) dropwise within 15 minutes. The reaction was stirred at this temperature for another 45 minutes and then quenched with saturated $NH_4Cl_{(aq)}$ (1.0 mL). The mixture was diluted by EtOAc (10 mL), warmed back to room temperature, and passed through a short celite plug to filter the gelatinous substance. The organic layer was separated and the aqueous layer was extracted by EtOAc (10 mL×3). The combined organic layers were washed with brine, dried over anhydrous Na_2SO_4, filtered, and concentrated in vacuo to give the crude material as a pale-yellow solid which was directly used without further purification.

The crude from the previous step was dissolved in CH_2Cl_2 (46 mL, 0.026 M to **(+)-S2**). Montmorillonite K10 clay (1.22 g, 1 g/ 1 mmol of **(+)-S2**) was added at room temperature and the reaction was stirred for 1 hour. After completion, the clay was filtered by a short celite plug and the filtrate was concentrated in vacuo. The crude material was purified by flash column chromatography on silica gel (EtOAc/*n*-hexane, 3/7) to afford compound **(−)-S3** (212 mg, 70% yield) as a white solid, and its epimer compound **(+)-S3’** (41 mg, 14% yield) as a pale-brown solid. Compound **(−)-S3** (212 mg, 86% *ee*) was dissolved in $CHCl_3$ (6.7 mL) at refluxing temperature. After cooling down and kept at room temperature overnight, enantiopure compound **(−)-S3** (118 mg, >99% *ee*) was obtained as a pale-yellow crystal. Single crystal of compound **(−)-S3** was grown in CH_2Cl_2/*n*-hexane, and the absolute configuration was ascertained by X-ray diffraction analysis (CCDC-2207553). **^{1}H NMR (400 MHz, $CDCl_3$):** δ = 7.46 (dd, *J* = 5.1, 2.4 Hz, 1H), 4.65 (m, 1H), 3.84 (m, 1H), 3.66 (dd, *J* = 3.5, 1.0 Hz, 1H), 2.06 (d, *J* = 8.6 Hz, 1H). **^{13}C NMR (101 MHz, $CDCl_3$):** δ = 187.9, 152.1, 102.9, 65.6, 57.6, 51.7. ***ee*:** >99%, determined by HPLC analysis (Chiralpak AS column, flow rate = 1.0 mL/min, 10% IPA/*n*-hexane, abs = 254.8 nm), $t_{r(minor)}$ = 13.45 min, $t_{r(major)}$ = 15.64 min. $[\alpha]_D^{23} =$ −93.5 (c 1.0, acetone, >99% *ee*).

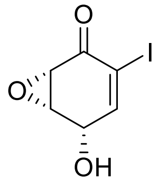

(+)-S3'

^{1}H NMR (400 MHz, $CDCl_3$): δ =7.28 (t, *J* = 2.6 Hz, 1H), 4.67 (dt, *J* = 10.9, 2.8 Hz, 1H), 3.90 (m, 1H), 3.67 (d, *J* = 3.9 Hz, 1H), 2.33 (d, *J* = 11.0 Hz, 1H). **^{13}C NMR (101 MHz, $CDCl_3$):** δ = 187.4, 153.4, 98.1. 67.4, 54.4, 51.3. **FTIR (neat):** 3358, 3042, 2918, 1686, 1596, 1331, 1317, 1236, 1059 cm^{-1}. **HRMS (EI) m/z:** $[M]^+$ Calcd for $C_6H_5IO_3$ 251.9283; Found $[M]^+$ 251.9283. $[\alpha]_D^{23} =$ +1.6 (c 1.0, acetone, 80% *ee*).

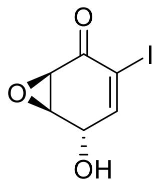

(+)-S3

The reaction was performed using compound **(-)-S2** (210 mg, 0.72 mmol, 1.0 equiv., 76% *ee*), DIBAL (1.08 mL, 1.08 mmol, 1.5 equiv.) and THF (7.2 mL); Montmorillonite K10 clay (724 mg) and CH_2Cl_2 (27 mL). Compound **(+)-S3** was obtained (122 mg, 67% yield) as a pale-brown solid. After recrystallization, enantiopure compound **(+)-S3** (68 mg) was obtained as a pale-yellow crystal. ***ee*:** >99%, determined by HPLC analysis (Chiralpak AS column, flow rate = 1.0 mL/min, 10% IPA/*n*-hexane, abs = 254.8 nm), $t_{r(major)}$ = 12.23 min, $t_{r(minor)}$ = 14.33 min. $[\alpha]_D^{25}$ = +92.4 (c 1.0, acetone, >99% *ee*).

Chemical synthesis of (−)-harveynone (13)

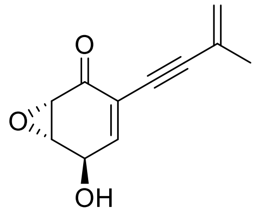

(-)-harveynone (**13**)

A flame-dried pressure tube charged with enantiopure compound **(−)-S3** (45 mg, 0.18 mmol, 1.0 equiv.), $Pd(PhCN)_2Cl_2$ (3.4 mg, 0.009 mmol, 5 mol%) and $AsPh_3$ (5.5 mg, 0.018 mmol, 10 mol%) was added a solution of tributyl(3-methylbut-3-en-1-yn-1-yl)stannane[6] (158 mg, 0.45 mmol, 2.5 equiv.) in degassed THF (4.5 mL, freeze-pump-thaw cycles). The reaction mixture was further degassed 3 times (freeze-pump-thaw cycle). After warming back to room temperature, the reaction was sealed under nitrogen and stirred for 16 hours. After completion, most of the solvent was removed under vacuo. The concentrated material was loaded on a short plug of silica gel and eluted with *n*-hexane (10 mL) and EtOAc (10 mL), respectively. The EtOAc eluent was concentrated, loaded on a short plug of silica gel, and eluted with *n*-hexane (10 mL) and then EtOAc (10 mL) sequentially again. The EtOAc layer was concentrated and purified by flash column chromatography on silica gel (EtOAc/*n*-hexane, 1/2) to afford (−)-harveynone (29 mg, 85% yield) as a pale brown solid. **^{1}H NMR (400 MHZ, $CDCl_3$):** δ = 6.84 (dd, *J* = 5.5, 2.5 Hz, 1H), 5.43 (s, 1H), 5.34 (s, 1H), 4.75 (dd, *J* = 8.4, 5.2 Hz, 1H), 3.82 (m, 1H), 3.56 (d, *J* = 3.6 Hz, 1H), 2.46 (d, *J* = 8.8 Hz, 1H), 1.93 (s, 3H). **^{13}C NMR (101 MHZ, $CDCl_3$):** δ = 190.8, 145.4, 25.9, 124.0, 123.0, 95.9, 81.2, 63.3, 57.4, 53.4, 23.0. **FTIR (neat):**

3447, 3097, 2976, 2956, 2921, 2205, 1933, 1691, 1616, 1434, 1375, 1238, 1114, 1071, 1040, 902, 848 cm^{-1}. **HRMS (FAB) m/z:** $[M+H]^+$ Calcd for $C_{11}H_{11}O_3$ 191.0708; Found $[M+H]^+$ 191.0707. $[\alpha]_D^{27} = -189.2$ (c 0.93, $CHCl_3$).

Chemical synthesis of (+)-harveynone (8)

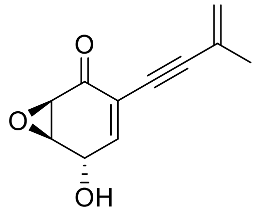

(+)-harveynone (**8**)

The reaction was performed using enantiopure compound **(+)-S3** (20 mg, 0.08 mmol, 1.0 equiv.), $Pd(PhCN)_2Cl_2$ (1.5 mg, 0.004 mmol, 5 mol%), $AsPh_3$ (2.4 mg, 0.008 mmol, 10 mol%), stannane (70 mg, 0.20 mmol, 2.5 equiv.) and degassed THF (2 mL). (+)-harveynone was obtained (12.5 mg, 83% yield) as a pale brown solid. **^{1}H NMR (400 MHZ, $CDCl_3$):** δ = 6.84 (dd, *J* = 5.2, 2.4 Hz, 1H), 5.43 (s, 1H), 5.35 (s, 1H), 4.75 (s, br), 3.83 (m, 1H), 3.56 (d, *J* = 3.6 Hz, 1H), 2.56 (d, *J* = 8.4 Hz, 1H), 1.94 (s, 3H). The ^{13}C NMR spectrum is identical to that of its enantiomer, (−)-harveynone (**13**). $[\alpha]_D^{28} = +202.1$ (c 0.33, $CHCl_3$).

Chemical synthesis of (+)-asperpentyn (1)

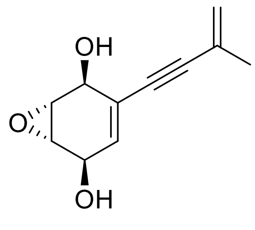

(+)-asperpentyn (**1**)

(-)-Harveynone (11 mg, 0.058 mmol, 1.0 equiv.), $CeCl_3{\cdot}7H_2O$ (43 mg, 0.116 mmol, 2.0 equiv.) were dissolved in methanol (0.6 mL) and the solution was cooled to -85°C (acetone/ liquid nitrogen bath). $NaBH_4$ (4.4 mg, 0.116 mmol, 2.0 equiv.) in methanol (0.4 mL) was added dropwise. The reaction was stirred at -85°C for 10 minutes and then quenched with saturated $NaHCO_{3(aq)}$ (1 mL). The aqueous layer was extracted with EtOAc (10 mL×3). The combined organic layers were washed with brine, dried over anhydrous

Na_2SO_4, filtered, and concentrated in vacuo. The crude material was purified by flash column chromatography on silica gel (EtOAc/*n*-hexane, 3/2) to afford (+)-asperpentyn (7.8 mg, 71% yield) as a colorless oil and *epi*-asperpentyn (3.2 mg, 29% yield) as a white solid. **^{1}H NMR (400 MHZ, $CDCl_3$):** δ = 6.07 (dd, *J* = 5.3, 1.7 Hz, 1H), 5.36 (m, 1H), 5.34 (m, 1H), 4.52 (m, 1H), 4.49 (d, *J* = 1.0 Hz, 1H), 3.41 (m, 1H), 3.34 (m, 1H), 3.50-3.10 (br, 1H), 1.91 (s, 1H). **^{13}C NMR (101 MHZ, $CDCl_3$):** δ = 131.3, 126.2, 123.2, 122.3, 92.2, 86.8, 65.6, 62.7, 52.2, 51.5, 23.2. **FTIR (neat):** 3355, 3006, 2920, 2199, 1636, 1608, 1454, 1433, 1374, 1300, 1024, 902, 838 cm^{-1}. **HRMS (EI) m/z:** $[M]^+$ Calcd for $C_{11}H_{12}O_3$ 192.0786; Found $[M]^+$ 192.0782. $[\alpha]_D^{30} =$ +20.8 (c 0.5, acetone).

Chemical synthesis of 1-*epi*-(+)-asperpentyn (14)

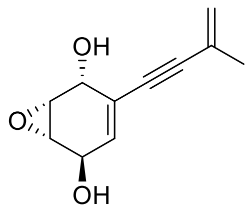

1-*epi*-(+)-asperpentyn (**14**)

^{1}H NMR (400 MHZ, $CDCl_3$): δ = 6.04 (m, 1H), 5.36 (s, 1H), 5.30 (m, 1H), 4.54 (t, *J* = 6.0 Hz, 1H), 4.49 (d, *J* = 7.8 Hz, 1H), 3.60 (t, *J* = 3.4 Hz, 1H), 3.50 (m, 1H), 2.23 (d, *J* = 8.1 Hz, 1H), 1.92 (s, 3H); 1.65 (d, *J* = 7.7 Hz, 1H). **^{13}C NMR (101 MHZ, $CDCl_3$):** δ = 131.3, 126.1, 123.5, 123.2, 93.2, 85.0, 65.2, 63.0, 55.2, 53.5, 23.2. **FTIR (neat):** 3383, 3006, 2918, 2849, 2199, 1634, 1608, 1434, 1374, 1302, 1029, 893, 845cm^{-1}. **HRMS (EI) m/z:** $[M]^+$ Calcd for $C_{11}H_{12}O_3$ 192.0786; Found $[M]^+$ 192.0787. $[\alpha]_D^{28} =$ +15.2 (c 0.5, acetone).

Chemical synthesis of (2*S*,3*R*)-α-epoxyquinone (10)

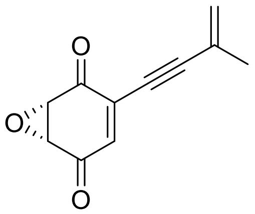

(2*S*,3*R*)-α-epoxyquinone (**10**)

To a solution of (−)-harveynone (29 mg, 0.15 mmol, 1.0 equiv.) dissolved in CH_2Cl_2 (1.5 mL, 0.1 M to (-)-harveynone) in a vial was added Dess-Martin periodinane (193 mg, 0.45 mmol, 3.0 equiv.) in one portion. The vial was sealed and the reaction mixture was stirred vigorously at room temperature for 12 hours. After completion, half of the

solvent was removed under vacuo, then the residue was purified by flash column chromatography on silica gel (EtOAc/*n*-hexane, 1/4) to afford **10** (20.1 mg, 70% yield) as a yellow oil. **^{1}H NMR (500 MHz, $CDCl_3$)** δ = 6.68 (d, *J* = 2.3 Hz, 1H), 5.55 (s, 1H), 5.48 (s, 1H), 3.89 (d, *J* = 3.8 Hz, 1H), 3.85 – 3.80 (dd, *J* = 3.8, 2.3 Hz, 1H), 1.97 (s, 3H). **^{13}C NMR (126 MHz, $CDCl_3$):** δ = 190.6, 188.2, 136.3, 132.6, 126.4, 125.6, 105.3, 81.2, 54.4, 53.9, 22.6. **FTIR (neat):** 3053, 2957, 2922, 2850, 2194, 1713, 1682, 1574, 1273 cm^{-1}. **HRMS (EI) m/z:** $[M]^+$ Calcd for $C_{11}H_8O_3$ 188.0473; Found $[M]^+$ 188.0476. $[\alpha]_D^{30}$ = +103.7 (c 0.22, $CHCl_3$).

Chemical synthesis of (2*R*,3*S*)-β-epoxyquinone (6)

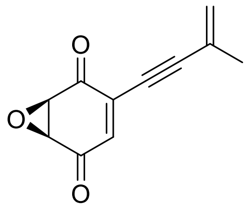

(2*R*,3*S*)-β-epoxyquinone **(6)**

The reaction was performed using (+)-harveynone (9.8 mg, 0.052 mmol, 1.0 equiv.), Dess-Martin periodinane (66 mg, 0.15 mmol, 3.0 equiv.) and CH_2Cl_2 (0.5 mL, 0.1 M to (+)-harveynone). Compound **6** was obtained (8.6 mg, 89% yield) as a yellow oil. **^{1}H NMR (400 MHz, $CDCl_3$)** δ = 6.68 (d, *J* = 2.4 Hz, 1H), 5.55 (s, 1H), 5.48 (s, 1H), 3.89 (d, *J* = 3.6 Hz, 1H), 3.83 – 3.81 (dd, *J* = 4.0, 2.4 Hz, 1H), 1.97 (s, 3H). The ^{13}C NMR spectrum is identical to that of its enantiomer, (2*S*,3*R*)-α-epoxyquinone (**10**). $[\alpha]_D^{26}$ = −109.5 (c 0.2, $CHCl_3$).

1.27 Chemical synthesis of siccayne (4)

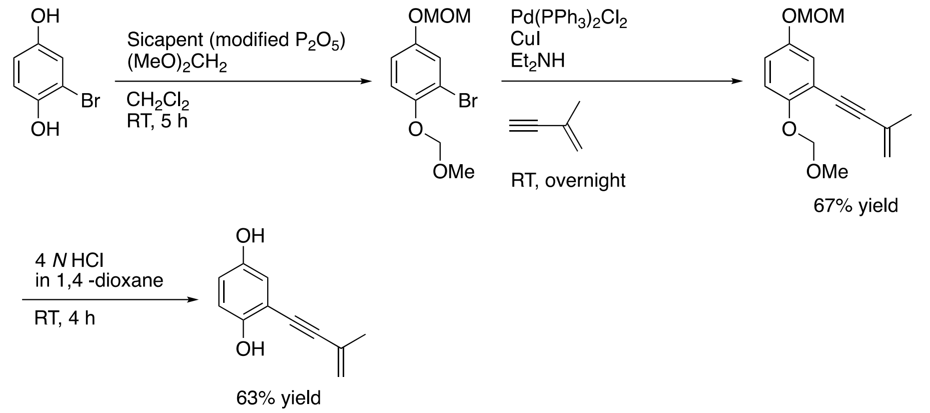

To the suspending solution of 2-bromobenzene-1,4-diol (200 mg, 1.05 mmol), Merck Sicapent® (800 mg) in dry CH_2Cl_2 (0.1 M, 11 mL) was slowly injected the $(MeO)_2CH_2$ (20 equiv., 1.9 mL) with stirring at room temperature under nitrogen atmosphere. During which, the color of suspending solid slowly turned to purple from original white. 5 hours later, the TLC tracing showed there was no starting material left and the reaction mixture was filtered and the filtrate was washed with saturated $NaHCO_{3(aq)}$. The combined organic layer was dried over $MgSO_4$, filtered and concentrated *in vacuo* to give the crude in an analytical purity, which will be used for next step directly without further purification.

To above MOM-protected compound, $Pd(PPh_3)_2Cl_2$ (1 mol%, 7.4 mg) and CuI (1 mol%, 2.0 mg) in a flame-dried RB flask was sequentially added the Et_2NH (0.15 M, 7.1 mL) and 2-methyl-1-buten-3-yne (3.0 equiv., 0.3 mL) with stirring at room temperature under nitrogen atmosphere overnight. At which, the reaction mixture was passed through a short pad of Celite 545 and the filtrate was extracted with EtOAc and water. The combined organic layer was sequentially washed with 1 *N* $HCl_{(aq)}$, saturated $NaHCO_{3(aq)}$ and brine, which was dried over $MgSO_4$, filtered and concentrated under reduced pressure to give the dryness residue. The resulting residue was purified on the Büchi Pure C-850 Flash-Prep automated purification machine with the disposable silica gel cartridge (EtOAc/*n*-hexanes), monitored by ELSD/UV scan detectors to give the Sonogashira coupled compound (186 mg, 67% yield over 2 steps) as a reddish oil. The Sonogashira coupling compound (186 mg, 0.71 mmol) was treated with the commercial reagent of 4 *N* HCl in 1,4-dioxane (0.1 M, 7.1 mL) with stirring at room temperature under nitrogen atmosphere for 4 hours. At which, 10 mL of ether was added with stirring and the resulting precipitates were collected and washed with a mixture of hexanes and ether (1/1, v/v). The washed compounds were kept under high vacuum for few hours to give the desired siccayne (78 mg, 63% yield) as a pale yellow solid.

R_f = 0.32 (EtOAc/Hexanes, 1/2, v/v); **^{1}H NMR (500 MHz, $CDCl_3$):** δ = 6.83–6.81 (m, 2H), 6.75 (dd, *J* = 8.7, 2.9 Hz, 2H), 5.43 (s, 1H), 5.40 (s, 1H), 5.36 (s, 1H), 4.65 (s, 1H), 2.00 (s,

3H); **^{13}C NMR (125 MHz, $CDCl_3$):** δ = 151.1, 148.9, 126.3, 123.2, 118.2, 117.6, 115.7, 110.2, 97.9, 82.1, 23.6. **HRMS (ESI-TOF):** ***m/z*** calcd for $C_{11}H_8O_2$ $[M+H]^+$ 175.0759; found 175.0753 .

1.28 Chemical synthesis of 2-methylenyne-benzoquinone (5)

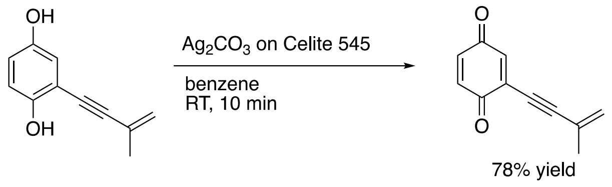

The Ag_2CO_3 (63 mg, 0.22mmol) on Celite 545 was prepared in advance: to an equal amount of Ag_2CO_3 and Celite 545 mixture in RB flask was added the dried acetonitrile, the resulting mixture was allowed to stir at room temperature overnight. To a mixture of the siccayne (20 mg, 0.1 mmol), Ag_2CO_3 (63 mg, 0.22 mmol) on Celite 545 was slowly injected the benzene (0.57 mL) with stirring at room temperature under nitrogen atmosphere. After being stirred for 10 minutes, the reaction mixture was filtered through the 33 mm diameter of Milipore Millex-LCR Hydrophilic PTFE 0.45 um micro-disc. The filtrate was concentrated under reduced pressure to give the crude, which was chromatographically purified on the Büchi Pure C-850 Flash-Prep automated purification machine with the disposable silica cartridge (EtOAc/Hexanes), monitored by ELSD/UV scan detectors to afford the desired product (15.6 mg, 78%) as a yellowish oil.

R_f = 0.60 (EtOAc/Hexanes, 1/2, v/v); **^{1}H NMR (500 MHz, $CDCl_3$):** δ = 6.88 (s, 1H, Ar-H), 6.79–6.79 (m, 2H, Ar-H), 5.57 (s, 1H, alkenyl H), 5.48 (t, *J* = 1.5 Hz, 1H, germinal alkenyl H), 2.00 (s, 3H, CH_3); **^{13}C NMR (101 MHZ, $CDCl_3$):** δ = 186.5, 183.0, 137.0, 136.4, 136.2, 132.4, 125.9, 125.8, 105.2, 81.2, 22.7. **FTIR (neat):** 3061, 2957, 2923, 2853, 2196, 1672, 1649, 1575, 1281, 1084, 902 cm^{-1}. **HRMS (ESI-TOF):** ***m/z*** calcd for $C_{11}H_8O_2$ $[M+Na]^+$ 195.0417; found 195.0363.

1.29 Chemical synthesis of (−)-1*R*-hydroxy-asperpenone (7)

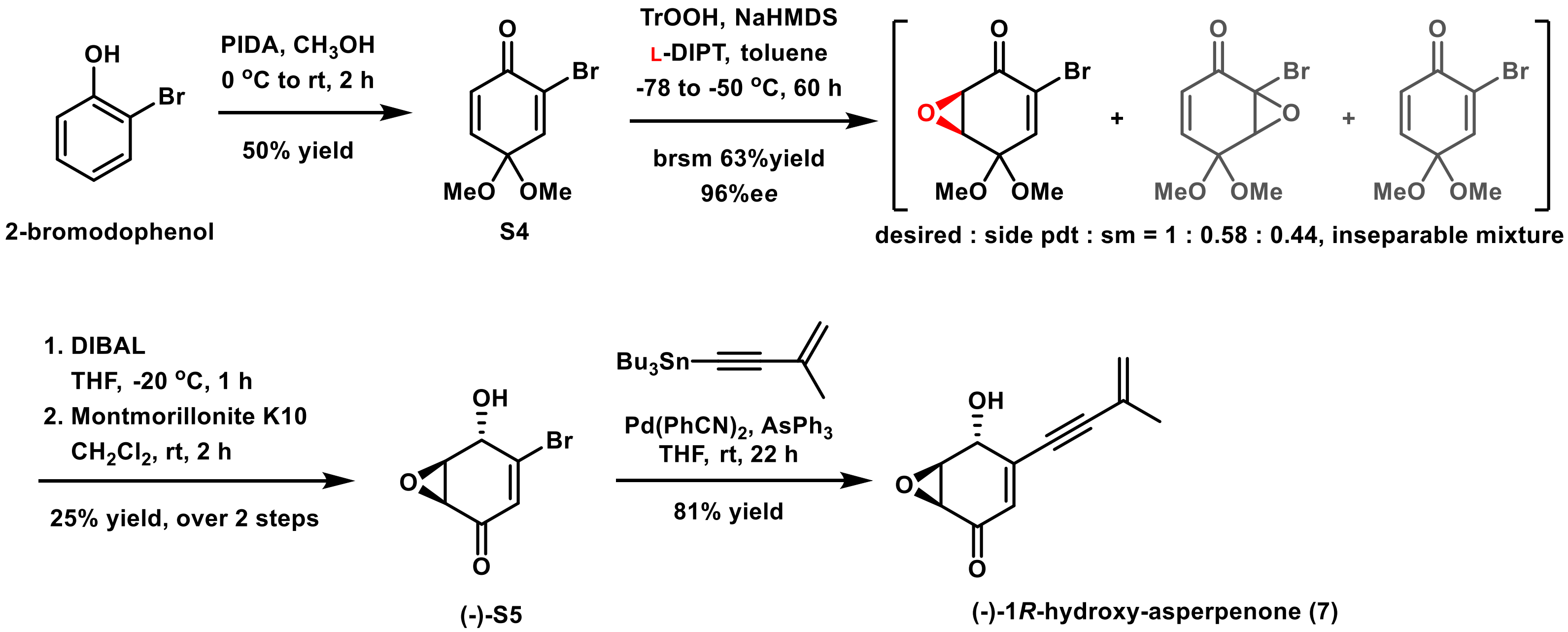

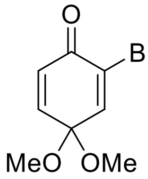

S4

To a stirred solution of 2-bromophenol (1.0 g, 5.8 mmol, 1.0 equiv.) in anhydrous methanol (23 mL, 0.25 M to 2-bromophenol) at 0°C was added $PhI(OAc)_2$ (4.3 g, 13.3 mmol, 2.3 equiv.) in one portion. The ice bath was removed and the resultant was stirred at room temperature for 2 hours. After completion, sat. $NaHCO_{3(aq)}$ was added at 0°C until the gas emission stopped. The resulting mixture was extracted with Et_2O (30 mL×3) and water. The combined organic layers were washed with brine, dried over anhydrous Na_2SO_4, filtered, and concentrated in vacuo. The crude material was purified by flash column chromatography on silica gel (Et_2O/*n*-pentane, 1/4) to afford compound **S4** (682 mg, 50% yield) as a yellow oil. **^{1}H NMR (400 MHz, $CDCl_3$):** δ = 7.29 (d, *J* = 3.0 Hz, 1H), 6.85 (dd, *J* = 10.1, 3.0 Hz, 1H), 6.39 (d, *J* = 10.1 Hz, 1H), 3.39 (s, 6H). **^{13}C NMR (101 MHz, $CDCl_3$):** δ = 177.8, 144.0, 143.6, 128.3, 126.0, 94.4, 50.6.[7]

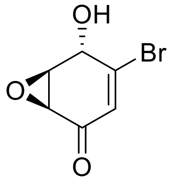

(-)-S5

To a freshly prepared solution of trityl hydroperoxide in toluene (4.9 mL, 5.1 mmol, 6.0 equiv., ca. 1.05M) in a schlenk tube at -78°C was added NaHMDS (2.4 mL, 4.7 mmol, 5.5 equiv., 2.0 M solution in THF) dropwise. The transparent yellow solution was stirred at -78°C for 30 minutes. L-DIPT (223 mg, 0.95 mmol, 1.1 equiv.) in toluene (1.1 mL) was added dropwise at -78°C and then stirred at this temperature for further 1 hour. Compound **S4** (200 mg, 0.86 mmol, 1.0 equiv.) in toluene (2.0 mL) was added dropwise and the green reaction mixture was warmed to -50°C and stirred at this temperature for 60 hours. The reaction was quenched with water (10 mL) and extracted with EtOAc (10 mL× 3). The combined organic layers were washed with brine, dried over anhydrous Na_2SO_4, filtered, and concentrated *in vacuo*. The crude material was purified by flash column chromatography on silica gel (EtOAc/*n*-hexane, 1/9) to afford a mixture of products (144 mg, 63% yield brsm) as a yellowish oil.

To a stirred solution mixture from the previous step (118 mg, total 0.48 mmol, 1.0

equiv., containing ca. 59 mg of the desired compound) in anhydrous THF (4.8 mL, 0.1 M) at -20°C was added DIBAL (0.72 mL, 0.72 mmol, 1.5 equiv., 1.0 M solution in toluene) dropwise within 15 minutes. The reaction was stirred at this temperature for further 45 minutes and then quenched with saturated $NH_4Cl_{(aq)}$ (1.0 mL). The mixture was diluted by EtOAc (10 mL), warmed back to room temperature, and passed through a short celite plug to filter the gelatinous substance. The organic layer was separated and the aqueous layer was extracted by EtOAc (10 mL×3). The combined organic layers were washed with brine, dried over anhydrous Na_2SO_4, filtered, and concentrated in vacuo to give the crude material as a yellowish oil which was directly used without further purification.

The crude from the previous step was dissolved in CH_2Cl_2 (18 mL, 0.026 M). Montmorillonite K10 clay (480 mg) was added at room temperature and the reaction was stirred for 2 hours. After completion, the clay was filtered by a short celite plug and the filtrate was concentrated *in vacuo*. The crude material was purified by flash column chromatography on silica gel (EtOAc/*n*-hexane, 1/3) to afford compound **(−)-S5** (12 mg, 25% yield) as a yellowish oil. **^{1}H NMR (400 MHz, $CDCl_3$):** δ = 6.45 (d, *J* = 1.6 Hz, 1H), 4.83 (s, 1H), 3.87 (dd, *J* = 3.5, 1.6 Hz, 1H), 3.54 – 3.48 (m, 1H), 2.98 (br, 1H). **^{13}C NMR (101 MHz, $CDCl_3$):** δ = 190.3, 145.7, 129.9, 69.9, 56.9, 52.3. ***ee*:** 96%, determined by HPLC analysis (Chiralpak AS column, flow rate = 1.0 mL/min, 10% IPA/*n*-hexane, abs = 254.8 nm), $t_{r(major)}$ = 13.50 min, $t_{r(minor)}$ = 19.53 min. $[\alpha]_D^{30}$ = −198.1 (c 1.0, CH_3OH, 96% *ee*).[8]

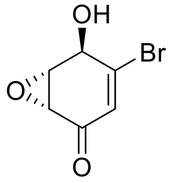

(+)-S5

The reaction was performed using trityl hydroperoxide in toluene (4.9 mL, 3.4 mmol, 4.0 equiv., ca. 0.70M), NaHMDS (1.5 mL, 3.0 mmol, 3.5 equiv., 2.0 M solution in THF), L-DIPT (223 mg, 0.95 mmol, 1.1 equiv.) in toluene (1.1 mL) and compound **S4** (200 mg, 0.86 mmol, 1.0 equiv.) in toluene (2.0 mL); The product mixture from step 1 (65 mg, total 0.26 mmol, 1.0 equiv., containing ca. 37 mg of the desired compound), DIBAL (0.4 mL, 0.40 mmol, 1.5 equiv., 1.0 M solution in toluene), anhydrous THF (2.6 mL, 0.1 M); Montmorillonite K10 clay (260 mg), CH_2Cl_2 (10 mL, 0.026M). Compound **(+)-S5** was obtained (10 mg, 33% yield) as a colorless oil. ***ee*:** 97%, determined by HPLC analysis (Chiralpak AS column, flow rate = 1.0 mL/min, 10% IPA/*n*-hexane, abs = 254.8 nm), $t_{r(minor)}$ = 13.70 min, $t_{r(major)}$ = 19.39 min. $[\alpha]_D^{28}$ = +200.3 (c 0.83, CH_3OH, 97% *ee*).[8]

Chemical synthesis of (−)-1*R*-hydroxy-asperpenone (7)

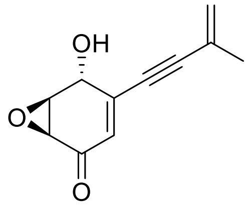

(-)-1*R*-hydroxy-asperpenone (**7**)

A flame-dried pressure tube charged with $Pd(PhCN)_2Cl_2$ (1.2 mg, 0.003 mmol, 5 mol%) and $AsPh_3$ (1.8 mg, 0.006 mmol, 10 mol%) was added a solution of compound **(−)-S5** (12 mg, 0.058 mmol, 1.0 equiv.) and tributyl(3-methylbut-3-en-1-yn-1-yl)stannane (52 mg, 0.145 mmol, 2.5 equiv.) in degassed THF (1.5 mL, freeze-pump-thaw cycles, 0.04 M to compound **(−)-S5**). The reaction mixture was further degassed 3 times (freeze-pump-thaw cycle). After warmed back to room temperature, the reaction was sealed under nitrogen and stirred for 22 hours. After completion, most of the solvent was removed under vacuo. The concentrated material was loaded on a short plug of silica gel and eluted with *n*-hexane (10 mL) and EtOAc (10 mL), respectively. The EtOAc eluent was concentrated, loaded on a short plug of silica gel, and eluted with n-hexane (10 mL) and then EtOAc (10 mL) sequentially again. The EtOAc layer was concentrated and purified by flash column chromatography on silica gel (EtOAc/*n*-hexane, 1/3) to afford compound **7** (9 mg, 81% yield) as a yellow oil. **^{1}H NMR (400 MHZ, $CDCl_3$):** δ = 6.15 (d, *J* = 1.6 Hz, 1H), 5.48 (s, 1H), 5.44 (s, 1H), 4.68 (d, *J* = 5.2 Hz, 1H), 3.80 (m, 1H), 3.47 (m, 1H), 2.57 (d, *J* = 6.0 Hz, 1H), 1.95 (s, 3H). **^{13}C NMR (101 MHZ, $CDCl_3$):** δ = 192.6, 138.8, 129.4, 125.81, 125.77, 101.8, 85.6, 66.3, 56.5, 52.5, 23.0. **FTIR (neat):** 3417, 2956, 2923, 2853, 2193, 1668, 1612, 1590, 1455, 1434, 1347, 1303, 1262, 1054, 1020, 919, 891, 861 cm^{-1}. **HRMS (EI) m/z:** $[M]^+$ Calcd for $C_{11}H_{10}O_3$ 190.0630; Found $[M]^+$ 190.0630. $[\alpha]_D^{26}$ = −327.4 (c 0.75, $CHCl_3$, 96% *ee*).

Chemical synthesis of (+)-1*S*-hydroxy-asperpenone (11)

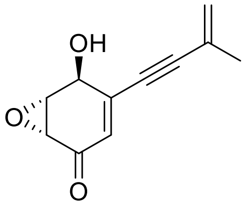

(+)-1*S*-hydroxy-asperpenone (**11**)

The reaction was performed using compound **(+)-S5** (9 mg, 0.044 mmol, 1.0 equiv.), $Pd(PhCN)_2Cl_2$ (0.8 mg, 0.002 mmol, 5 mol%), $AsPh_3$ (1.2 mg, 0.004 mmol, 10 mol%), tributyl(3-methylbut-3-en-1-yn-1-yl)stannane (42 mg, 0.11 mmol, 2.5 equiv.) and degassed THF (1.1 mL). Compound **11** was obtained (6.5 mg, 78% yield) as a yellow oil. **^{1}H NMR (400 MHZ, $CDCl_3$):** δ = 6.15 (d, *J* = 1.6 Hz, 1H), 5.48 (s, 1H), 5.44 (s, 1H), 4.68 (d, *J* = 5.2 Hz, 1H), 3.80 (m, 1H), 3.47 (m, 1H), 2.61 (d, *J* = 6.0 Hz, 1H), 1.95 (s, 3H). The ^{13}C NMR spectrum is identical to that of its enantiomer, (−)-1*R*-hydroxy-asperpenone (**7**). $[\alpha]_D^{23} =$ +345.9 (c 0.4, $CHCl_3$, 97% *ee*).

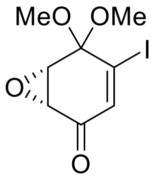

(+)-S2

Chiralpak AS column, flow rate = 1.0 mL/min, 10% IPA/*n*-hexane, abs = 254.8 nm

$t_{r(minor)}$ = 18.04 min, $t_{r(major)}$ = 24.56 min; **(+)-S2** with 85% *ee*

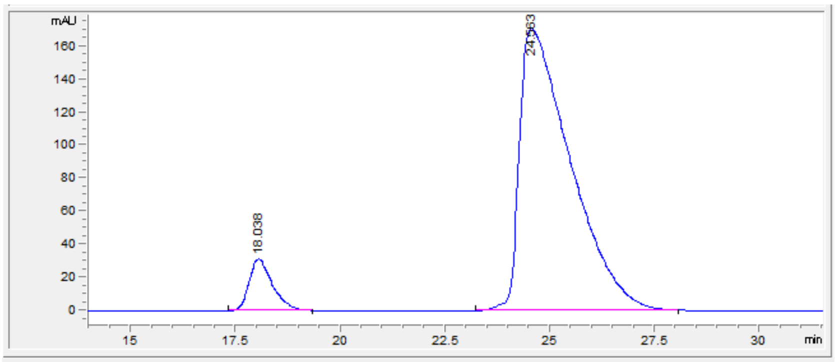

#	Time	Area	Height	Width	Area%	Symmetry
1	18.038	1206.2	31.1	0.5806	7.700	0.633
2	24.563	14458.9	170.8	1.2397	92.300	0.297

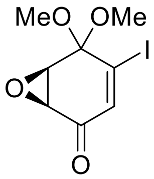

(-)-S2

Chiralpak AS column, flow rate = 1.0 mL/min, 10% IPA/*n*-hexane, abs = 254.8 nm

$t_{r(major)}$ = 15.88 min, $t_{r(minor)}$ = 22.36min; **(−)-S2** with 76% *ee*

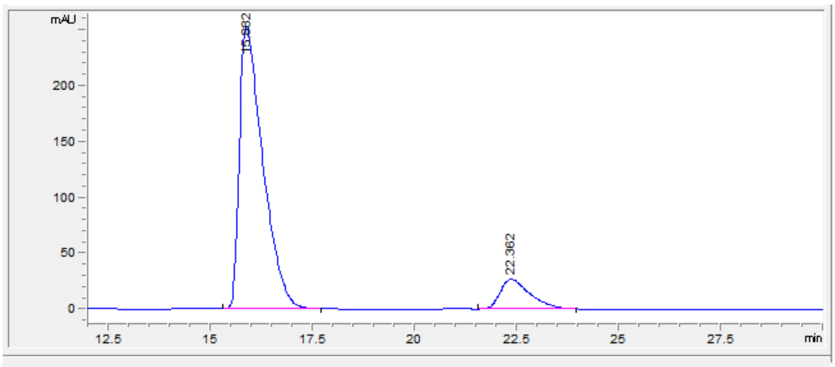

#	Time	Area	Height	Width	Area%	Symmetry
1	15.882	10109.6	252.4	0.5974	88.259	0.398
2	22.362	1344.8	26.5	0.7507	11.741	0.553

HPLC chromatogram of compound **(+)-S2** and **(−)-S2.**

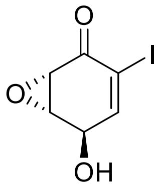

(-)-S3

Chiralpak AS column, flow rate = 1.0 mL/min, 10% IPA/*n*-hexane, abs = 254.8 nm)

$t_{r(minor)}$ = 13.80 min, $t_{r(major)}$ = 15.64 min; **(−)-S3** with >99% *ee*

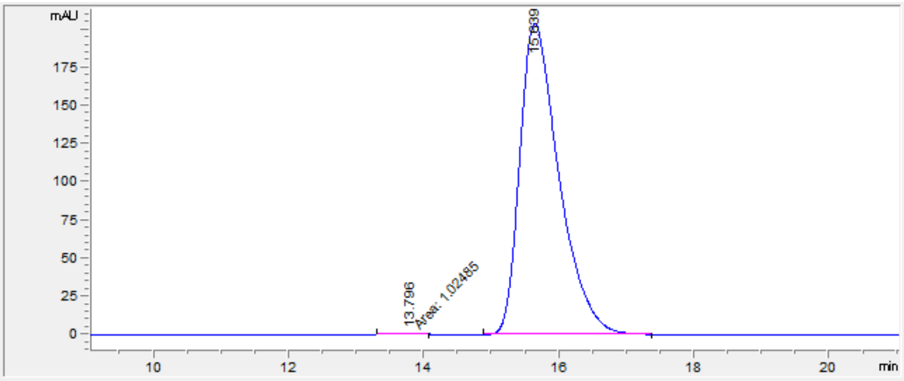

#	Time	Area	Height	Width	Area%	Symmetry
1	13.796	1	1.1E-1	0.1491	0.012	1.231
2	15.639	8221.8	203.7	0.6088	99.988	0.586

HPLC chromatogram of recrystallized **(−)-S3**

Chiralpak AS column, flow rate = 1.0 mL/min, 10% IPA/*n*-hexane, abs = 254.8 nm)

$t_{r(minor)}$ = 13.45 min, $t_{r(major)}$ = 15.88 min; **(-)-S3** with 54% *ee*

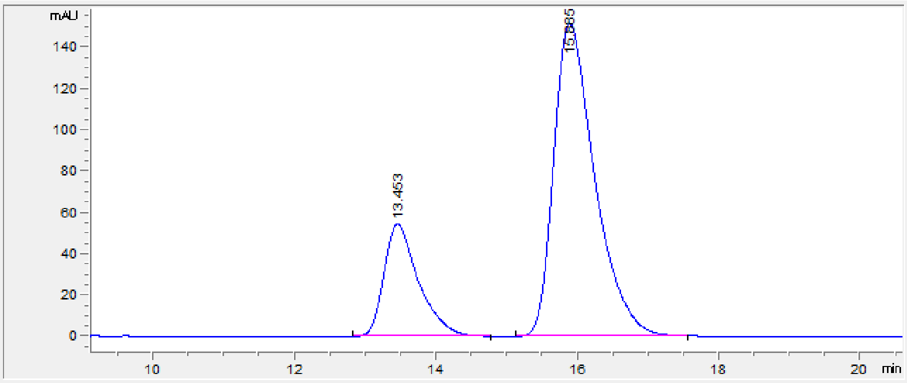

#	Time	Area	Height	Width	Area%	Symmetry
1	13.453	1849.7	54.4	0.5069	23.183	0.613
2	15.885	6129.2	151.2	0.6089	76.817	0.601

HPLC chromatogram of the mother liquor of **(−)-S3.**

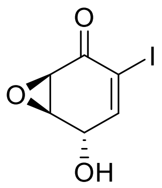

(+)-S3

Chiralpak AS column, flow rate = 1.0 mL/min, 10% IPA/*n*-hexane, abs = 254.8 nm

$t_{r(major)}$ = 12.35 min, $t_{r(minor)}$ = 14.64 min; **(+)-S3** with > 99% *ee*

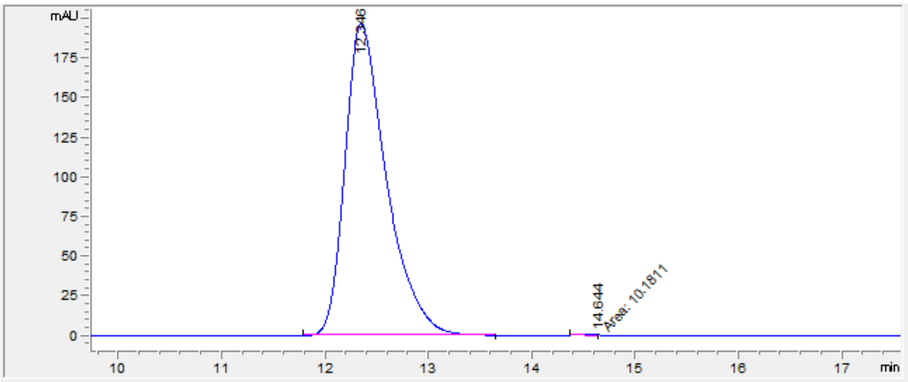

#	Time	Area	Height	Width	Area%	Symmetry
1	12.346	5502.7	196.5	0.4187	99.815	0.612
2	14.644	10.2	1.3	0.1261	0.185	0.181

HPLC chromatogram of recrystallized **(+)-S3**

Chiralpak AS column, flow rate = 1.0 mL/min, 10% IPA/*n*-hexane, abs = 254.8 nm)

$t_{r(major)}$ = 12.23 min, $t_{r(minor)}$ = 14.34 min; **(+)-S3** with 51% *ee*

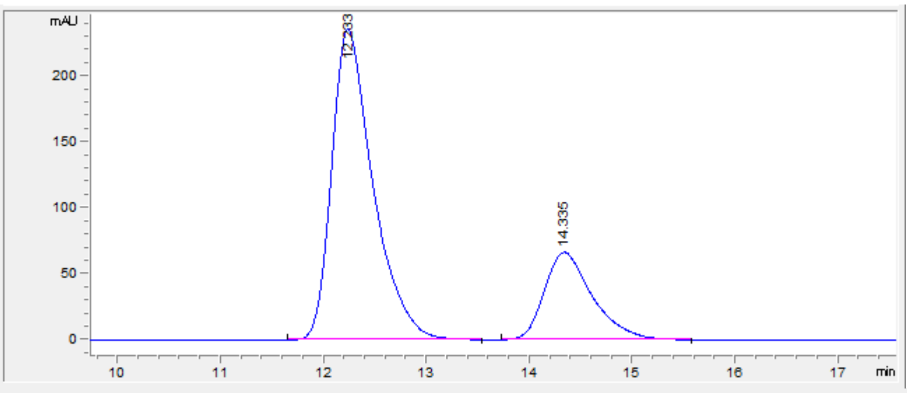

#	Time	Area	Height	Width	Area%	Symmetry
1	12.233	6578	235.7	0.4156	75.470	0.607
2	14.335	2138.1	66.2	0.483	24.530	0.653

HPLC chromatogram of the mother liquor of **(+)-S3**

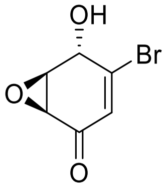

(-)-S5

Chiralpak AS column, flow rate = 1.0 mL/min, 10% IPA/*n*-hexane, abs = 254.8 nm

$t_{r(major)}$ = 13.50 min, $t_{r(minor)}$ = 19.53 min; **(−)-S5** with 96% *ee*

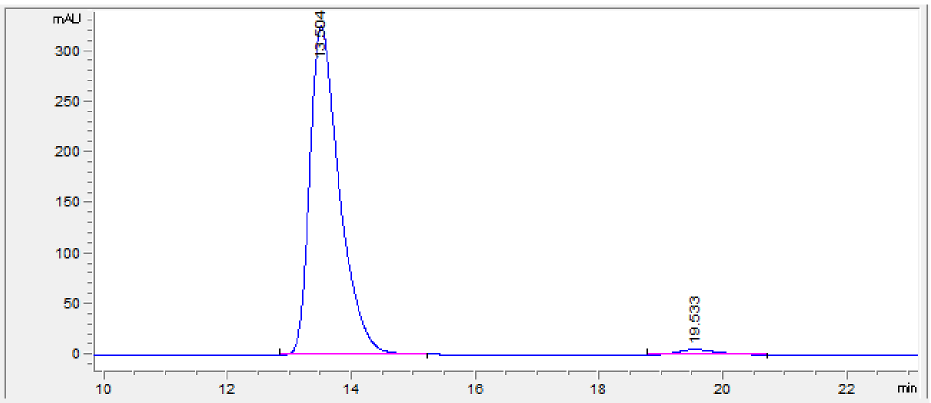

#	Time	Area	Height	Width	Area%	Symmetry
1	13.504	10742.3	324.5	0.4963	97.853	0.582
2	19.533	235.7	5.2	0.6497	2.147	0.707

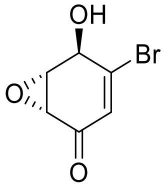

(+)-S5

Chiralpak AS column, flow rate = 1.0 mL/min, 10% IPA/*n*-hexane, abs = 254.8 nm)

$t_{r(minor)}$ = 13.70 min, $t_{r(major)}$ = 19.39 min; with 97% *ee*

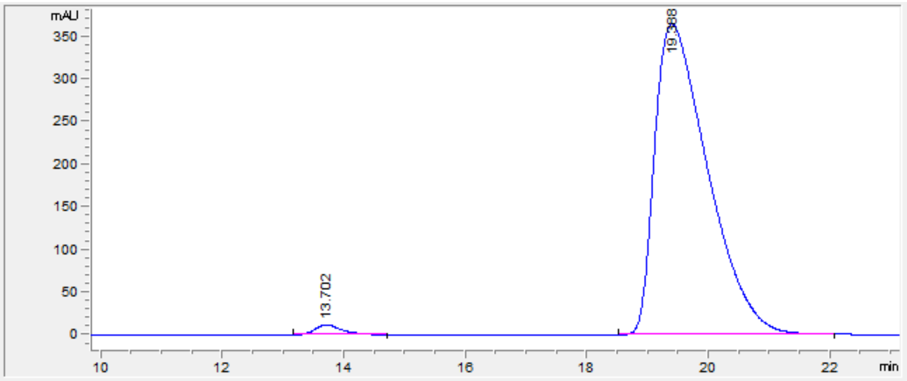

#	Time	Area	Height	Width	Area%	Symmetry
1	13.702	362.5	11.6	0.4633	1.615	0.666
2	19.388	22084.3	364.5	0.9268	98.385	0.447

HPLC chromatogram of **(−)-S5 and (+)-S5**

2. Supplementary Tables

Table S1. PCR primers used in this study.

Primer name	Sequence (5’→3’)
**s51_HBGI-pAdeA-M**	
PRs51-H-NotI-PamyB-F	TGAACAATAAACCCCACAGCAAGCTCCGGCGGCCGCATGCATCCTCCCACCCGCATGTTC
PRs51-H-NotI-TamyB-R	TCCACCCTTCACGAGCTACTACAGATCGCGGCCGCCTATACGTGATTCCCATTCGTAGTC
PRs51-B-PacI-Padh-F	CTTTCTTTCAACACAAGATCCCAAAGTCAAATTAATTAAATGGACCCCATCCAGTACAAC
PRs51-B-PacI-Tadh-R	TTCTATGCGTTATGAACATGTTCCCTTTAATTAATCACAACACATACCCATACTTCTTCG
PRs51-I-AscI-Peno-F	ACTGACCAATTCCGCAGCTCGTCAAAGGCGCGCCATGTTCCCCATCGACAGTATATACTC
PRs51-I-AscI-Teno-R	TGGTAGACGTCATATAATCATACGGTTCATGGCGCGCCTCATTTCCCCCCGAAGGTCGAG
PRs51-G-XhoI-PgpdA-F	CCAGCTGCTCTTTTCTTTTCTCTTTCTTTTCCCCTCGAGATGGCAGAGACGTTTGACCCC
PRs51-G-XhoI-TgpdA-R	AAAGCACATTCACATTACCCTTGAGCTCGTTCCTCGAGCTAAATCCGACTCAAACCCCCC
**s51_CDE-pTAex3-M**	
PRs51-E-NotI-PamyB-F	CTGAACAATAAACCCCACAGCAAGCTCCGGCGGCCGCATGGCTTCGGCGTGGCAACTCTG
PRs51-E-NotI-TamyB-R	TCTCCACCCTTCACGAGCTACTACAGATCGCGGCCGCTCACAACGCCTTGGTGTACTCGG
PRs51-D-PacI-Padh-F	CCTTTCTTTCAACACAAGATCCCAAAGTCAAATTAATTAAATGTCCCTCCCCACGTCCGC
PRs51-D-PacI-Tadh-R	ATTCTATGCGTTATGAACATGTTCCCTTTAATTAACTAGTGAGGAAGAAGGGACAGATCC
PRs51-C-XhoI-PgpdA-F	AGCTGCTCTTTTCTTTTCTCTTTCTTTTCCCCTCGAGATGACTGTTTACGTGATCACCGG
PRs51-C-XhoI-TgpdA-R	AGCACATTCACATTACCCTTGAGCTCGTTCCTCGAGTTACACCCACTGCTTGTTCCCCAG
**pMAL_MBP-AtyG**	
pMAL_atyG_F	CGGGATCGAGGGAAGGATTTCACATATGGCAGAGACGTTTGACCCC
pMAL_atyG_R	GAGCCTTTCGTTTTATTTGAAGCTTATctaAATCCGACTCAAACCCCC
**pColdI-His-AtyE**	
pColdI-AtyC-F	CATCATCATCATATCGAAGGTAGGCATATGATGGCTTCGGCGTGGCAACTCTGG
pColdI-AtyC-R	CCTATCTAGACTGCAGGTCGACCTACAACGCCTTGGTGTACTCGGGG
**pColdI-His-AtyC**	
pColdI-AtyC-F	CATCATCATCATATCGAAGGTAGGCATATGATGACTGTTTACGTGATCACCGG
pColdI-AtyC-R	CCTATCTAGACTGCAGGTCGACCTACACCCACTGCTTGTTCCCCA
**pColdI-His-AtyD**	
pColdI-AtyD-F	CATCATCATCATATCGAAGGTAGGCATATGATGTCCCTCCCCACGTCCGC
pColdI-AtyD-R	CCTATCTAGACTGCAGGTCGACCTAGTGAGGAAGAAGGGACAGATCCTCC
Primer name	Sequence (5'→3')
**pCold-AtyD-F97A**	

pCold-AtyD-F97A-F	GTC GCA GCG GTC GCG ACC CTC GAC CCG AAC
pCold-AtyD-F97A-R	GTT CGG GTC GAG GGT CGC GAC CGC TGC GAC
**pCold-AtyD-S135C**	
pCold-AtyD-S135C-F	GTG AAG CGC TTC GTG CTG ACC TCG TGC TCG CTG GCC GCC GTG CTG C
pCold-AtyD-S135C-R	GCA GCA CGG CGG CCA GCG AGC ACG AGG TCA GCA CGA AGC GCT TCA C
**pCold-AtyD-L137A**	
pCold-AtyD-L137A-F	CTG ACC TCG TCG TCG GCG GCC GCC GTG CTG CCC CAG
pCold-AtyD-L137A-R	CTG GGG CAG CAC GGC GGC CGC CGA CGA CGA GGT CAG
**pCold-AtyD-Y178F**	
pCold-AtyD-Y178F-F	GAG CCT TGC CCG TCT TTG CCG CCA GCA AGA C
pCold-AtyD-Y178F-R	GTC TTG CTG GCG GCA AAG ACG GGC AAG GCT C
**pCold-AtyD-K182A**	
pCold-AtyD-K182A-F	CCT TGC CCG TCT ACG CCG CCA GCG CGA CCC TGT CCG AGA AGG CGG CGT G
pCold-AtyD-K182A-R	CAC GCC GCC TTC TCG GAC AGG GTC GCG CTG GCG GCG TAG ACG GGC AAG G
**pCold-AtyD-N208A**	
pCold-AtyD-N208A-F	CAA TGC GGT CCT GCC CGC GAT GAA CTT TGG CGC CAG
pCold-AtyD-N208A-R	CTG GCG CCA AAG TTC ATC GCG GGC AGG ACC GCA TTG
**pCold-AtyD-Q246A**	
pCold-AtyD-Q246A-F	CTT GGG CGG GAT TAC CGC TGC ATA TTT TAT CGA CGT GCA AG
pCold-AtyD-Q246A-R	CTT GCA CGT CGA TAA AAT ATG CAG CGG TAA TCC CGC CCA AG

Table S2. ^1H NMR (500 Hz) and ^{13}C NMR (125 Hz) spectroscopic data of (−)-asperpentyn (**2**) in DMSO-d_6.

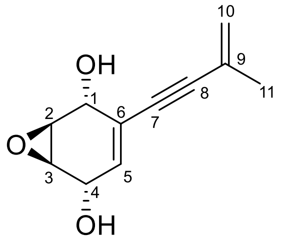

(-)-asperpentyn (**2**)

No.	δ_H (mult, J in Hz)	δ_C (type)
1	4.14 (m)	63.8 (CH)
2	3.20 (m)	53.0 (CH)
3	3.17 (m)	51.9 (CH)
4	4.27 (m)	61.2 (CH)
5	5.88 (dd, 4.9, 1.8)	132.7 (CH)
6		121.0 (C)
7		88.9 (C)
8		89.8 (C)
9		126.2 (C)
10	5.34 (m)	122.5 (CH_2)
	5.29 (m)	23.6 (CH_3)
11	1.87 (s)	23.1 (CH_3)

Table S3. ^{1}H NMR (500 Hz) and ^{13}C NMR (125 Hz) spectroscopic data of eutypinic acid (**3**) in $CDCl_3$.

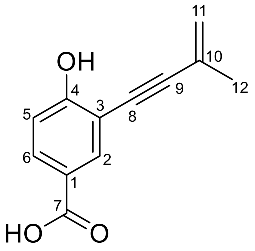

eutypinic acid (**3**)

No.	δ_H (mult, *J* in Hz)	δ_C (type)
1		121.9 (C)
2	8.15 (d, 2.1)	134.5 (CH)
3		110.1 (C)
4		160.5 (C)
5	7.03 (d, 8.6)	114.8 (CH)
6	8.01 (dd, 8.5, 2.0)	132.7 (CH)
7		170.3 (C)
8		80.7 (C)
9		98.5 (C)
10		125.8 (C)
11	5.43-5.42 (m)	123.7 (CH_2)
	5.50 (s)	
12	2.05 (s)	23.4 (CH_3)

Table S4. ^{1}H NMR (500 Hz) and ^{13}C NMR (125 Hz) spectroscopic data of siccayne (**4**) in $CDCl_3$

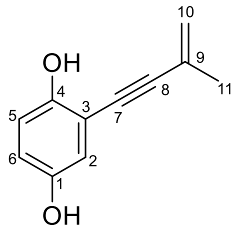

siccayne (**4**)

No.	δ_H (mult, *J* in Hz)	δ_C (type)
1		148.9 (C)
2	6.79 (d, 3.6)	117.6 (CH)
3		110.2 (C)
4		151.1 (C)
5	6.80 (d, 9.1)	115.7 (CH)
6	6.72 (dd, 8.7, 3.1)	118.2 (CH)
7		82.1 (C)
8		97.9 (C)
9		126.3 (C)
10	5.33, 5.34 (m)	123.2 (CH_2)
11	1.99 (dd, 1.7, 1.3)	23.6 (CH_3)
-OH	4.34 (s), 5.42 (m)	

Table S5. ^{1}H NMR (500 Hz), ^{13}C NMR (125 Hz) and 2D NMR spectroscopic data of 4-*epi*-(+)-asperpentyn (**12**) in DMSO-d_6

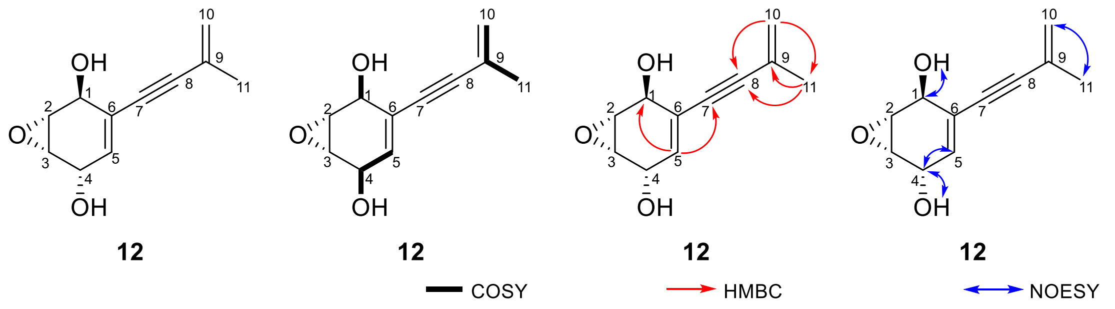

No.	δ_H (mult, J in Hz)	δ_C (type)	HMBC (δ_C)	COSY (δ_H)	NOESY (δ_H)
1	4.12 (d, 7.8)	64.6 (CH)		5.57	5.57
2	3.24 (br.d, 1,9)	53.7 (CH)		3.25	
3	3.25 (br.d, 1.8)	53.2 (CH)		3.24	
4	4.47 (m)	63.7 (CH)		5.41, 5.70	5.41, 5.70
5	5.70 (t, 2.2)	135.4 (CH)	64.6, 88.9	3.25, 4.47	4.47
6		120.5 (C)			
7		88.9 (C)*			
8		88.9 (C)*			
9		126.2 (C)			
10	5.32 (m), 5.28 (m)	122.4 (CH_2)	23.2, 88.9	1.87	1.87
11	1.87 (s)	23.2 (CH_3)	88.9, 122.4, 126.2	5.32, 5.28	5.32, 5.28
1-OH	5.57 (d, 7.7)			4.12	4.12
4-OH	5.41 (d, 6.9)			4.47	4.47

*The signals in ^{13}C spectrum were overlapped.

Crystal data and structure refinement for (–)-S3 (i18744)*.

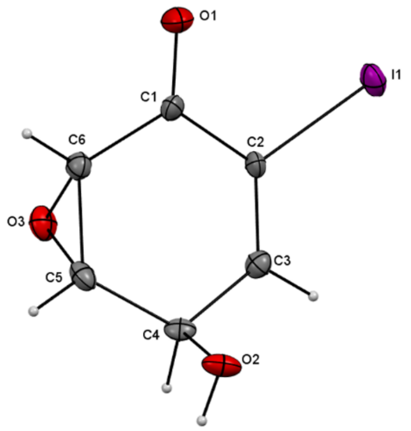

Identification code	i18744	
Empirical formula	C6 H5 I O3	
Formula weight	252.00	
Temperature	100.0(2) K	
Wavelength	0.71073 Å	
Crystal system	Monoclinic	
Space group	P 21	
Unit cell dimensions	a = 4.33810(10) Å	a= 90°.
	b = 11.4728(3) Å	b= 95.2270(10)°.
	c = 7.1816(2) Å	g = 90°.
Volume	355.943(16) $Å^3$	
Z	2	
Density (calculated)	2.351 mg/m^3	
Absorption coefficient	4.439 mm^{-1}	
F(000)	236	
Crystal size	0.212 x 0.174 x 0.132 mm^3	
Theta range for data collection	2.848 to 30.536°.	
Index ranges	-5<=h<=6, -16<=k<=16, -10<=l<=10	
Reflections collected	15257	
Independent reflections	2165 [R(int) = 0.0530]	
Completeness to theta = 25.242°	99.9 %	
Absorption correction	Numerical	
Max. and min. transmission	1 and 0.8076	
Refinement method	Full-matrix least-squares on F^2	
Data / restraints / parameters	2165 / 1 / 95	
Goodness-of-fit on F^2	1.220	
Final R indices [I>2sigma(I)]	R1 = 0.0182, wR2 = 0.0458	

R indices (all data)	R1 = 0.0215, wR2 = 0.0471
Absolute structure parameter	0.040(11)
Extinction coefficient	n/a
Largest diff. peak and hole	0.971 and -1.465 e.Å^{-3}

*CCDC-2207553, displacement ellipsoids are drawn at the 50% probability level.

3. Supplementary Figures

A

Synthetic asperpentyn racemates

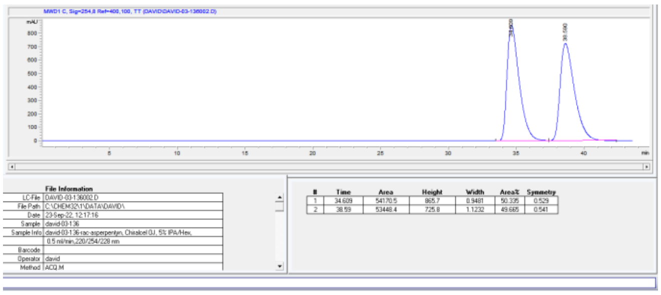

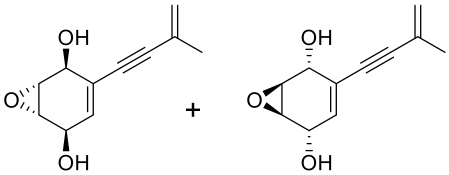

(+)-asperpentyn (**1**) (-)-asperpentyn (**2**)

Chemically synthesized (+)-asperpentyn

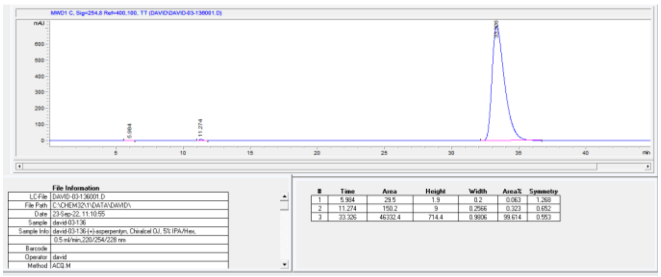

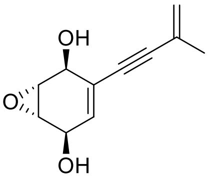

$[a]^{30}_{D}$ = +20.8, c =0.5, acetone

(+)-asperpentyn (**1**)

B

Chemically synthesized (+)-asperpentyn

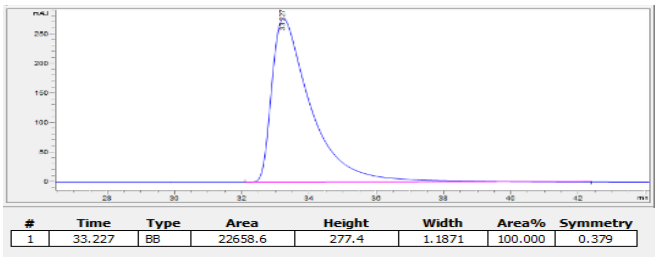

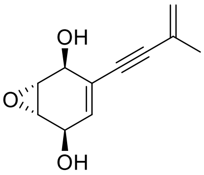

(+)-asperpentyn (**1**)

(–)-Asperpentyn isolated from *A. oryzae* expressing *atyHBIGECD*

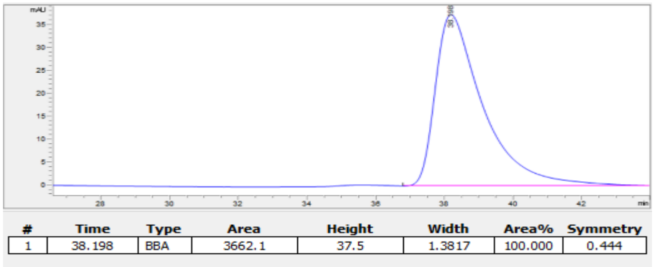

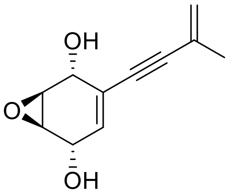

(-)-asperpentyn (**2**)

Figure S1. Chiral HPLC analysis of asperpentyn.

(A) HPLC chromatograms of chemically synthesized (+)-asperpentyn (**1**) and synthetic asperpentyn racemates, and (B) chemically synthesized **1** and (–)-asperpenyn (**2**) isolated from *A. oryzae* expressing *atyHBIGECD*. HPLC conditions: Chiralcel OJ column; mobile phase: 5% isopropanol in n-hexane; flow rate: 0.5 mL/min; detection at 254 nm (UV).

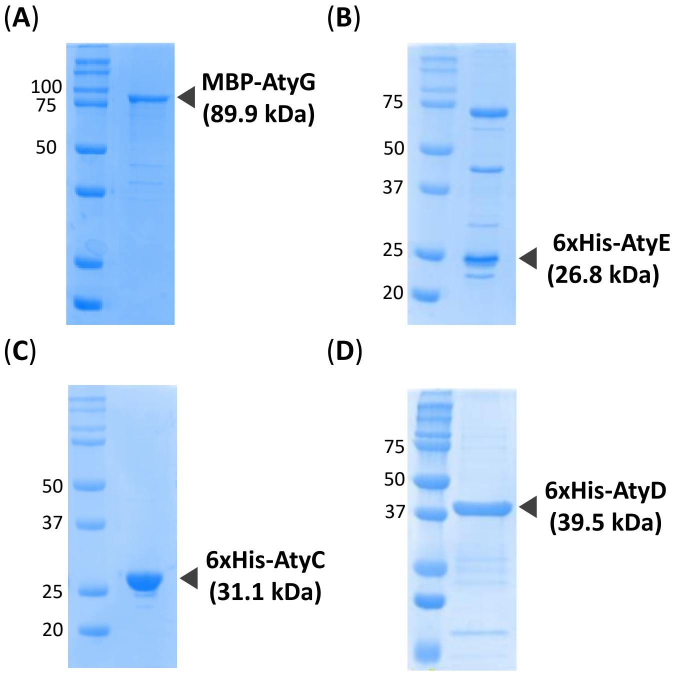

Figure S2. SDS-PAGE of purified AtyG, AtyE, AtyC and AtyD proteins**.**
(A) Purified AtyG with N-terminal maltose-binding protein (MBP) (89.9 kDa); (B) purified AtyE with N-terminal 6xHis tag (26.8 kDa); (C) purified AtyC with N-terminal 6xHis tag (31.1 kDa), (D) Purified AtyD with N-terminal 6xHis tag (39.5 kDa).

A

Chemically synthesized (+)-asperpentyn (**1**) spiked with (–)-asperpentyn (**2**) from AtyE/C/D with siccayne (**4**)

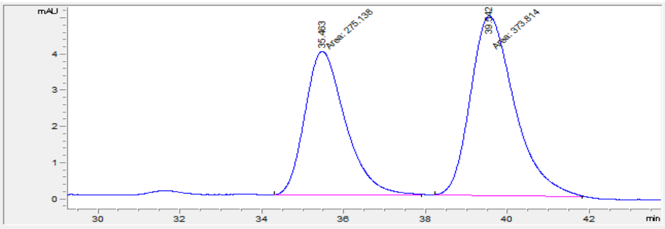

#	Time	Area	Height	Width	Area%	Symmetry
1	35.463	275.1	4	1.1512	42.397	0.723
2	39.542	373.8	5	1.2545	57.603	0.706

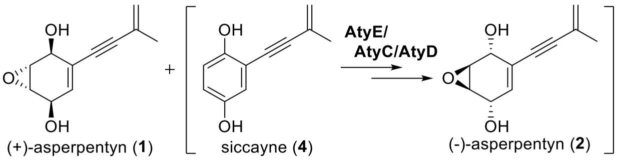

B

(–)-asperpentyn (**2**) from AtyE/C/D with siccayne (**4**)

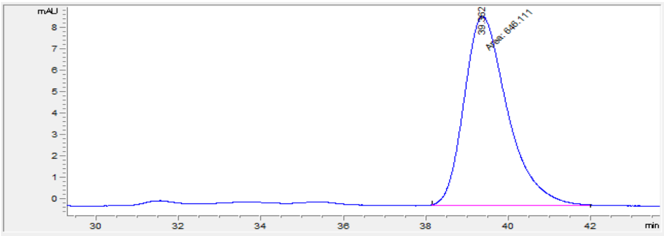

#	Time	Area	Height	Width	Area%	Symmetry
1	39.362	646.1	8.8	1.2172	100.000	0.704

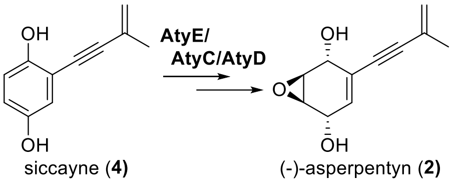

C

(–)-asperpentyn (**2**) produced from (2*R*,3*S*)-β-epoxyquinone (**6**) with AtyC/D

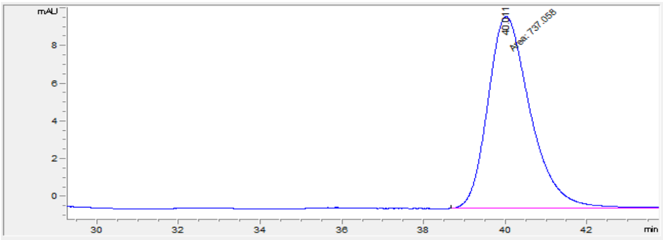

#	Time	Area	Height	Width	Area%	Symmetry
1	40.011	737.1	10.2	1.2077	100.000	0.716

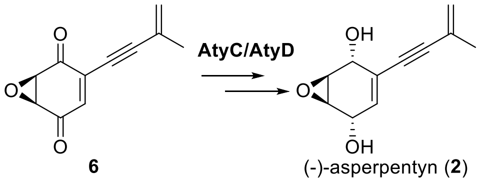

Figure S3. Chiral HPLC analysis of (–)-asperpentyn produced from *in vitro* enzymatic assays.

(A) Analysis of (+)-asperpentyn (**1**) spiked with (–)-asperpentyn (**2**), obtained from the reaction of siccayne (**4**) with AtyE, AtyC, and AtyD. (B) Analysis of **2** from the reaction of **4** with AtyE, AtyC, and AtyD. (C) Analysis of **2** produced from (2*S*,3*R*)-β-epoxyquinone (**6**) with AtyC and AtyD.

Conditions: Chiralcel OJ column; mobile phase: 5% isopropanol in n-hexane; flow rate: 0.5 mL/min; detection at 254 nm (UV).

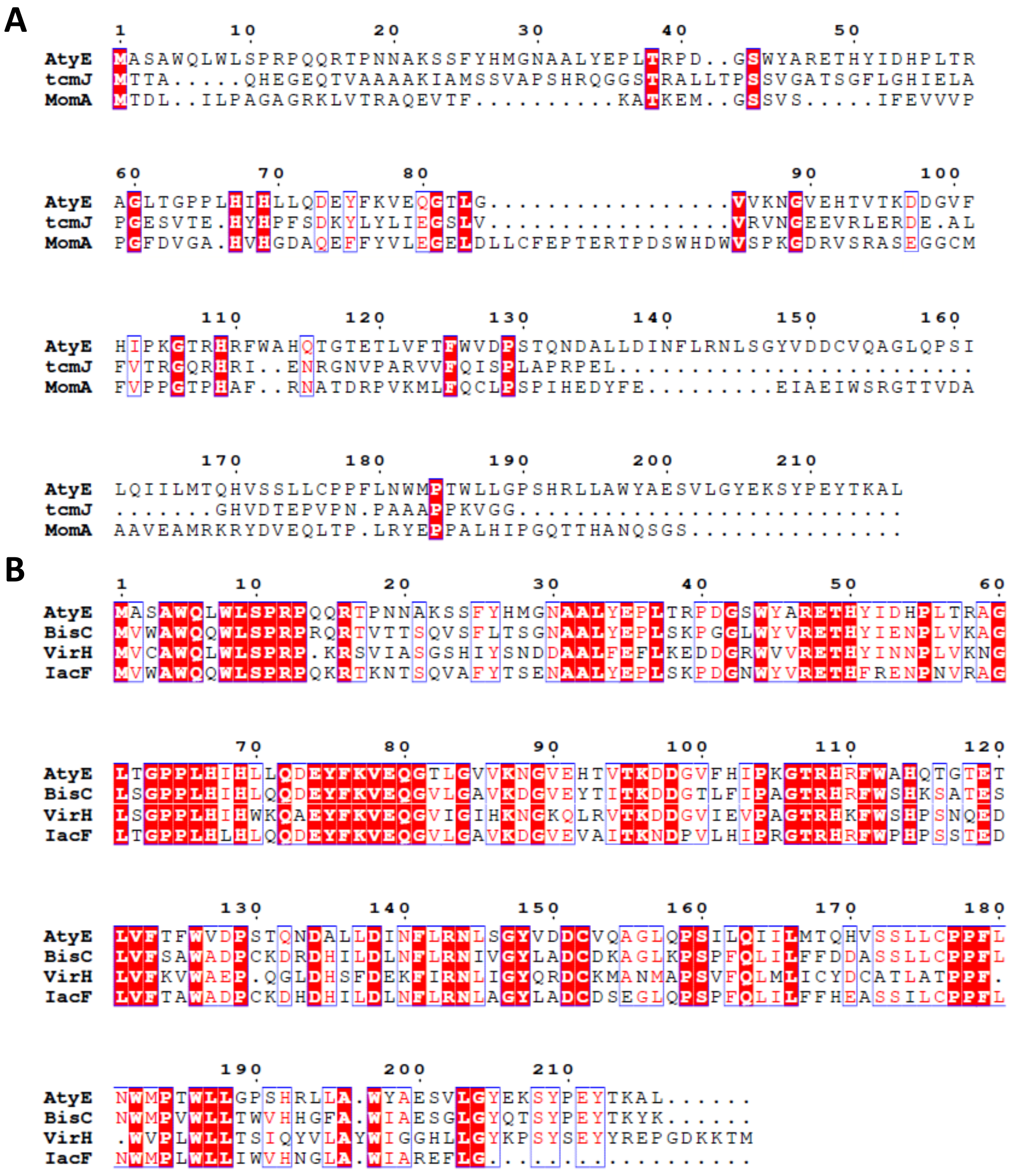

Figure S4. Protein sequence alignment of AtyE and its homologs.

(A) Alignment of AtyE with bacterial homologs TcmJ (accession no. P16558.2) and MomA (accession no. BAD89290.1). (B) Alignment of AtyE with fungal homologs BisC (accession no. QJQ82457.1), VirH (accession no. XP_013952631.1), and IacF (accession no. A0A1J0HSL4.1) from mushrooms.

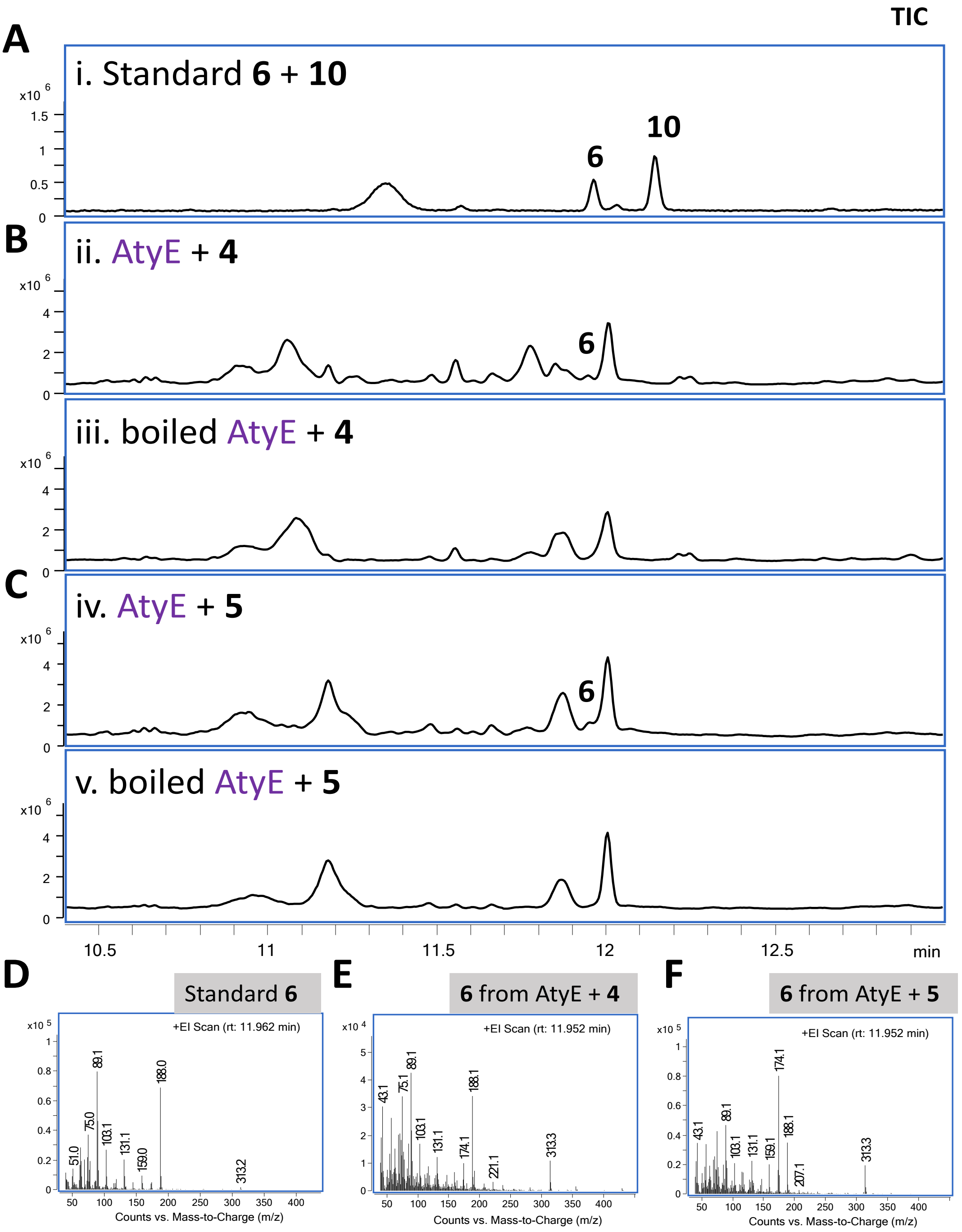

Figure S5. GC-EI-MS chromatograms of *in vitro* assays with AtyE.

Total ion chromatograms (TIC) of (A) synthetic standards **6** and **10**, (B) the *in vitro* assay of AtyE with substrate **4**, (C) the *in vitro* assay of AtyE with substrate **5**. (EI-MS spectra below) (D) Standard **6**; (E) Product **6** from the AtyE + **4** reaction; (F) Product **6** from the AtyE + **5** reaction.

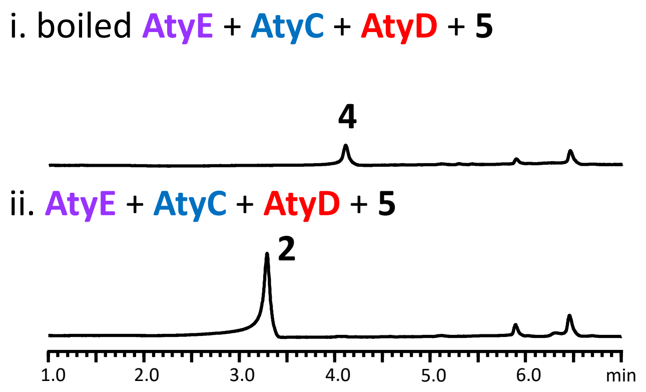

Figure S6. LC-DAD-MS analysis of *in vitro* enzymatic reactions of AtyE, AtyC, and AtyD. LC-DAD-MS chromatograms (UV detection at λ = 257 nm) of *in vitro* reactions containing AtyE, AtyC, and AtyD, in the presence of divalent metal ions, NADPH, and **5**.

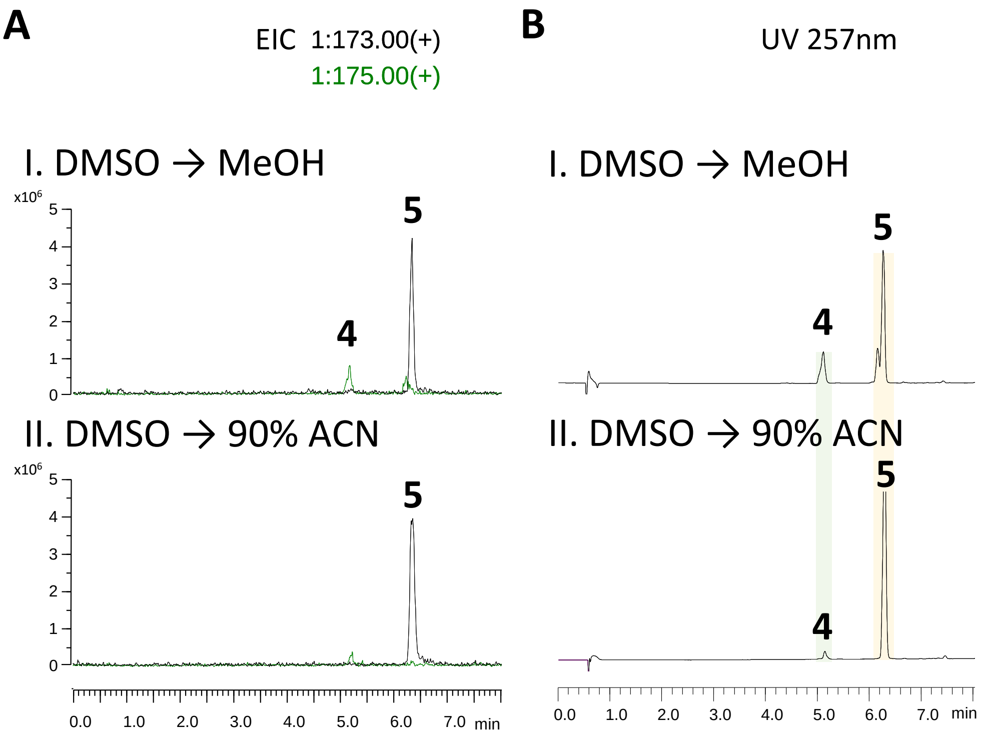

Figure S7. LC-DAD-MS analysis of the stability of 2-methylenyne-benzoquinone (**5**) in different solvent environments.

(A) Extracted ion chromatograms (EIC) and (B) UV chromatograms (λ = 257 nm) of standard **5** prepared in various solvents.

(i) Stock solution in DMSO diluted 100-fold into methanol.

(ii) Stock solution in DMSO diluted 100-fold into 90% acetonitrile (ACN).

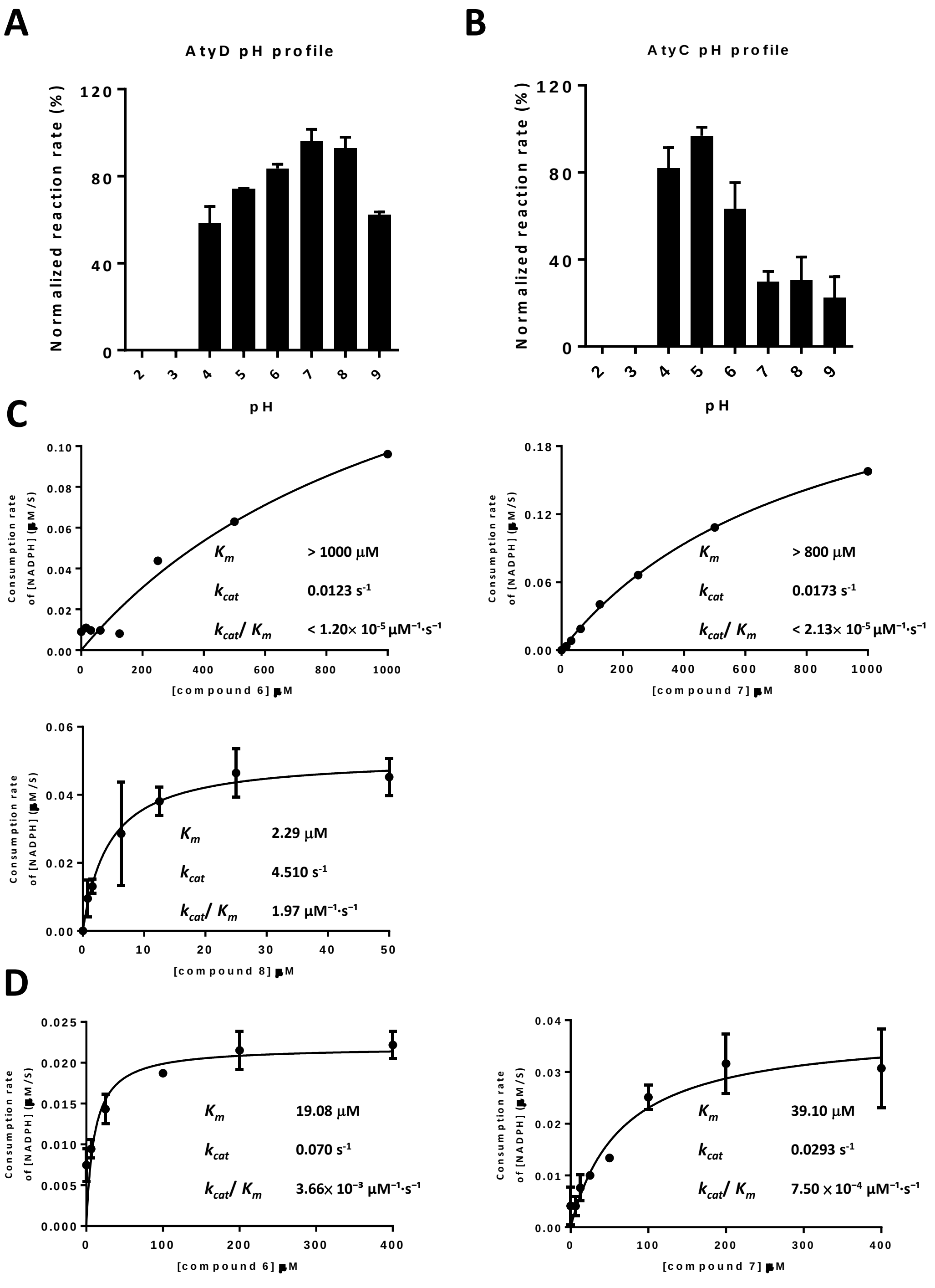

Figure S8. Steady-state kinetic analyses of AtyC and AtyD.

The pH dependence of enzymatic activity of (A) AtyD using compound **6** as the substrate. (B) AtyC using compound **8** as the substrate. The relative activity at each pH was normalized to the maximum activity, which was set as 100%. The bar charts were generated in GraphPad Prism (version 6). (C) Michaelis–Menten plots of AtyC with compounds **6**, **7**, or **8**. (D) Michaelis–Menten plots of AtyD with compound **6** or **7**. Kinetic parameters and plots were generated by GraphPad Prism (version 6).

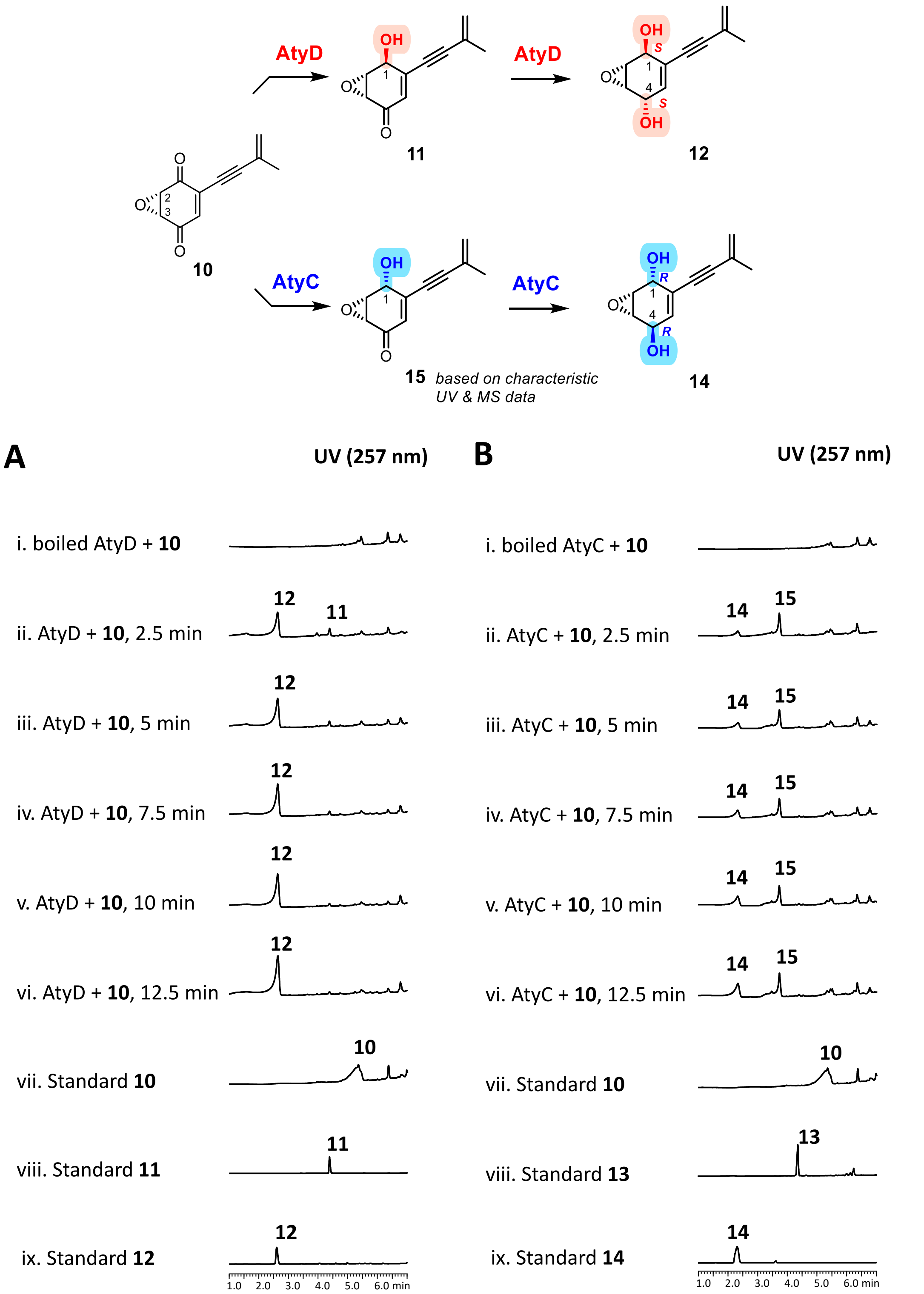

Figure S9. LC-DAD-MS analysis of time-course *in vitro* assays of AtyD and AtyC with (2*S*,3*R*)-α-epoxyquinone (**10**).

LC-DAD-MS chromatograms (UV detection at λ = 257 nm) showing time-dependent product formation in *in vitro* assays of (A) AtyD, and (B) AtyC, with NADPH and **10**.

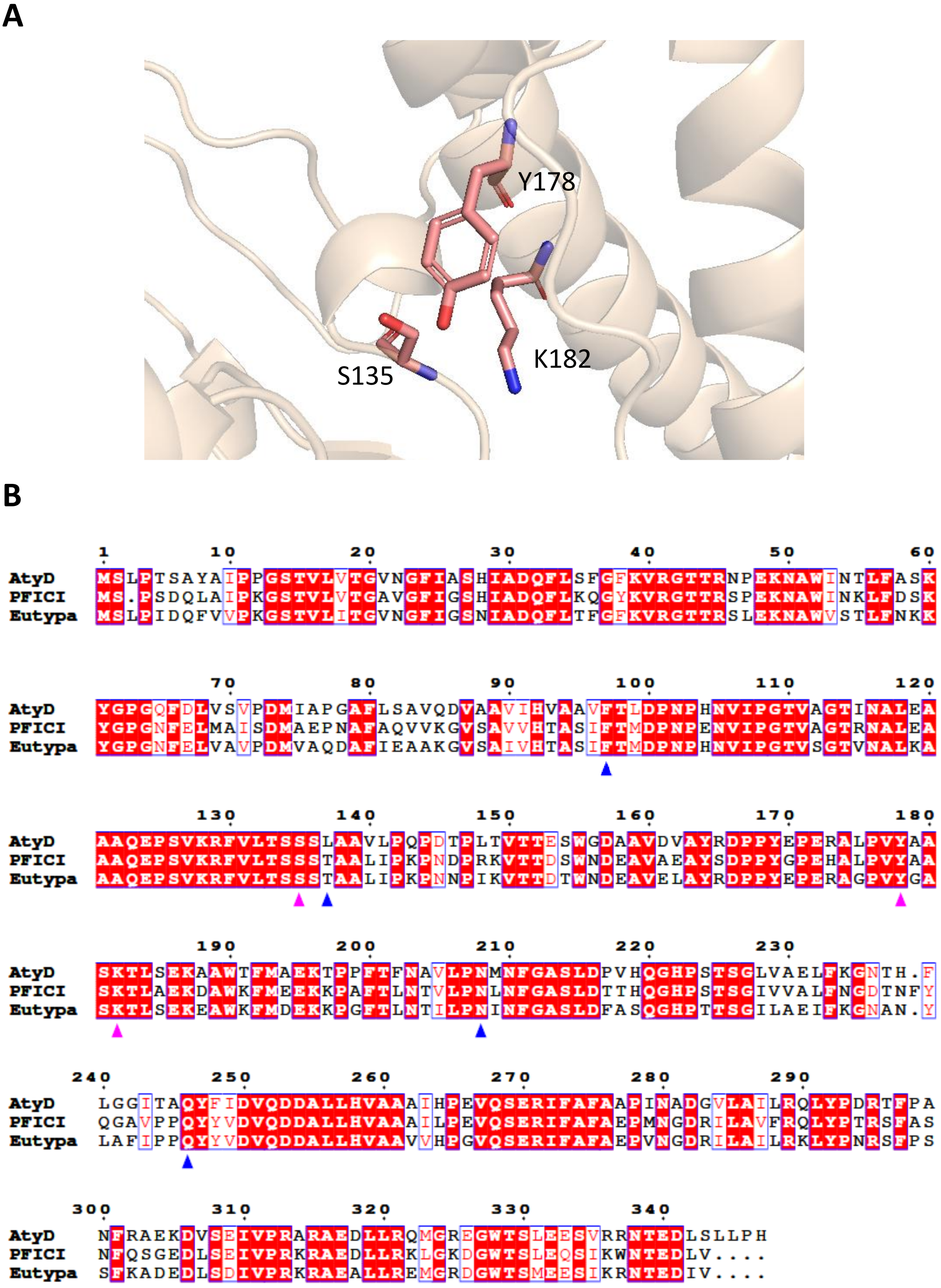
A
Y178
K182
S135
B
1 10 20 30 40 50 60
AtyD MSLPTSAYAIPPGSTVLVTGVNGFIASHIADQFLSFGFKVRGTTRNPEKNAWINTLFASK
PFICI MS.PSDQLAIPKGSTVLVTGAVGFIGSHIADQFLKQGYKVRGTTRSPEKNAWINKLFDSK
Eutypa MSLPIDQFVVPKGSTVLITGVNGFIGSNIADQFLTFGFKVRGTTRSLEKNAWVSTLFNKK
70 80 90 100 110 120
AtyD YGPGQFDLVSVPDMIAPGAFLSAVQDVAAVIHVAAVFTLDPNPHNVIPGTVAGTINALEA
PFICI YGPGNFELMAISDMAEPNAFAQVVKGVSAVVHTASIFTMDPNPENVIPGTVAGTRNALEA
Eutypa YGPGNFELVAVPDMVAQDAFIEAAKGVSAIVHTASIFTMDPNPHNVIPGTVSGTVNALKA
130 140 150 160 170 180
AtyD AAQEPSVKRFVLTSSSLAAVLPQPDTPLTVTTESWGDAAVDVAYRDPPYEPERALPVYAA
PFICI AAQEPSVKRFVLTSSSTAALIPKPNDPRKVTTDSWNDEAVAEAYSDPPYGPEHALPVYAA
Eutypa AAQEPSVKRFVLTSSSTAALIPKPNNPIKVTTDTWNDEAVELAYRDPPYEPERAGPVYGA
190 200 210 220 230
AtyD SKTLSEKAAWTFMAEKTPPFTFNAVLPNMNFGASLDPVHQGHPSTSGLVAELFKGNTH.F
PFICI SKTLAEKDAWKFMEEKKPAFTLNTVLPNLNFGASLDTTHQGHPSTSGIVVALFNGDTNFY
Eutypa SKTLSEKEAWKFMDEKKPGFTLNTILPNINFGASLDFASQGHPTTSGILAEIFKGNAN.Y
240 250 260 270 280 290
AtyD LGGITAQYFIDVQDDALLHVAAAIHPEVQSERIFAFAAPINADGVLAILRQLYPDRTFPA
PFICI QGAVPPQYYVDVQDDALLHVAAAILPEVQSERIFAFAEPMNGDRILAVFRQLYPTRSFAS
Eutypa LAFIPPQYYVDVQDDALLHVAAVVHPGVQSERIFAFAEPVNGDRILAILRKLYPNRSFPS
300 310 320 330 340
AtyD NFRAEKDVSEIVPRARAEDLLRQMGREGWTSLEESVRRNTEDLSLLPH
PFICI NFQSGEDLSEIVPRKRAEDLLRKLGKDGWTSLEQSIKWNTEDLV....
Eutypa SFKADEDLSDIVPRKRAEALLREMGRDGWTSMEESIKRNTEDIV....

(continued)

C

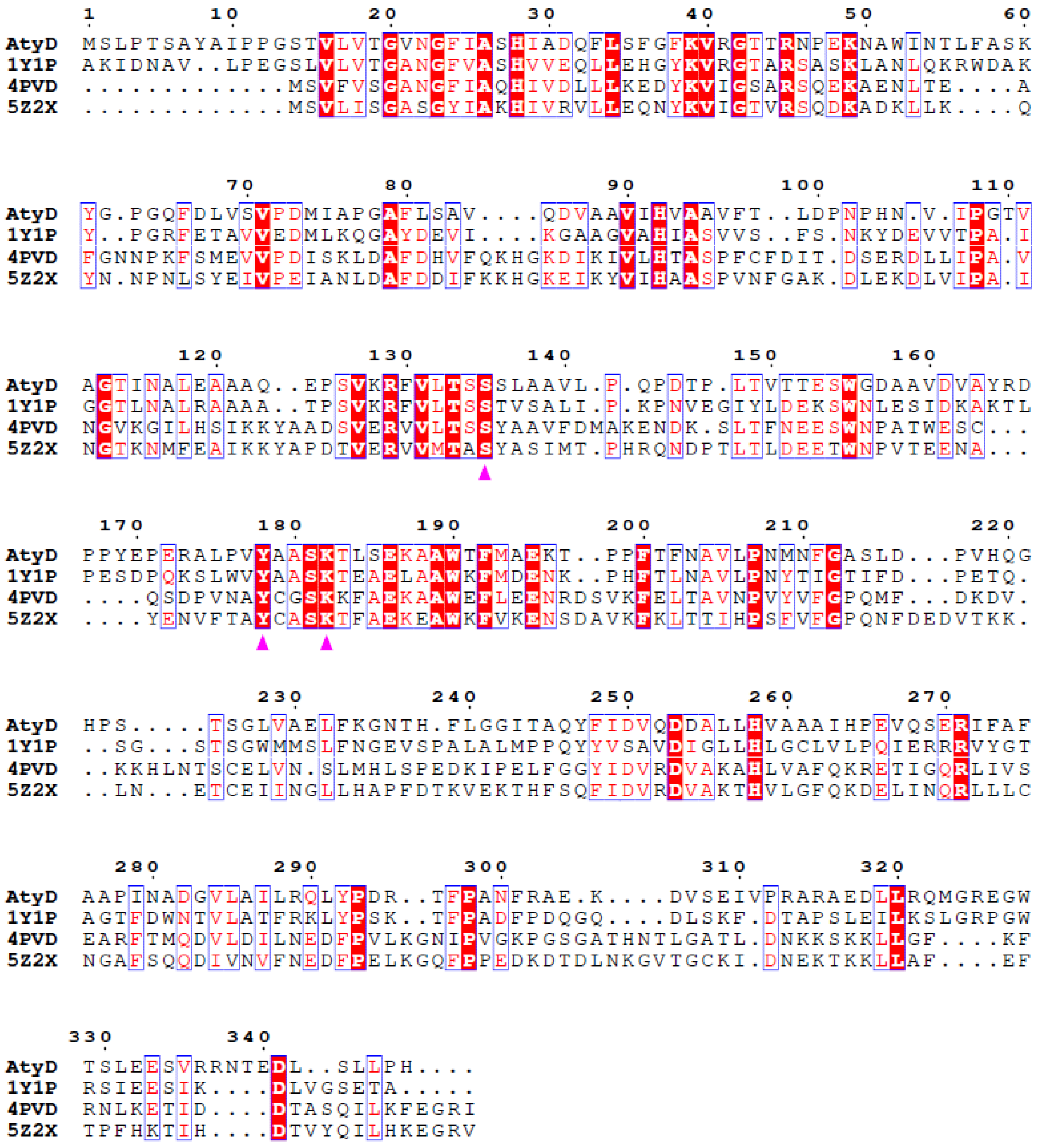

Figure S10. AlphaFold3 structural model of AtyD and sequence alignment with homologous enzymes.

(A) Predicted structure of AtyD generated using AlphaFold3. The inset highlights the three catalytic residues: Ser135, Tyr178, and Lys182. (B) Protein sequence alignment of AtyD with homologous aldehyde reductases from Ascomycetes. (C) Protein sequence alignment of AtyD with homologs from yeast and Basidiomycetes. Catalytic residues are marked with pink triangles, and residues for mutagenesis in this study are indicated with blue triangles. Homologs include *Pestalotiopsis fici* W106-1 (PFICI_07945, accession no. XP_007834717.1), *Eutypa lata* UCREL1 (accession no. EMR66145.1), and proteins with known structures: 1Y1P (PDB ID: 1Y1P), 4PVD (PDB ID: 4PVD), and 5Z2X (PDB ID: 5Z2X).

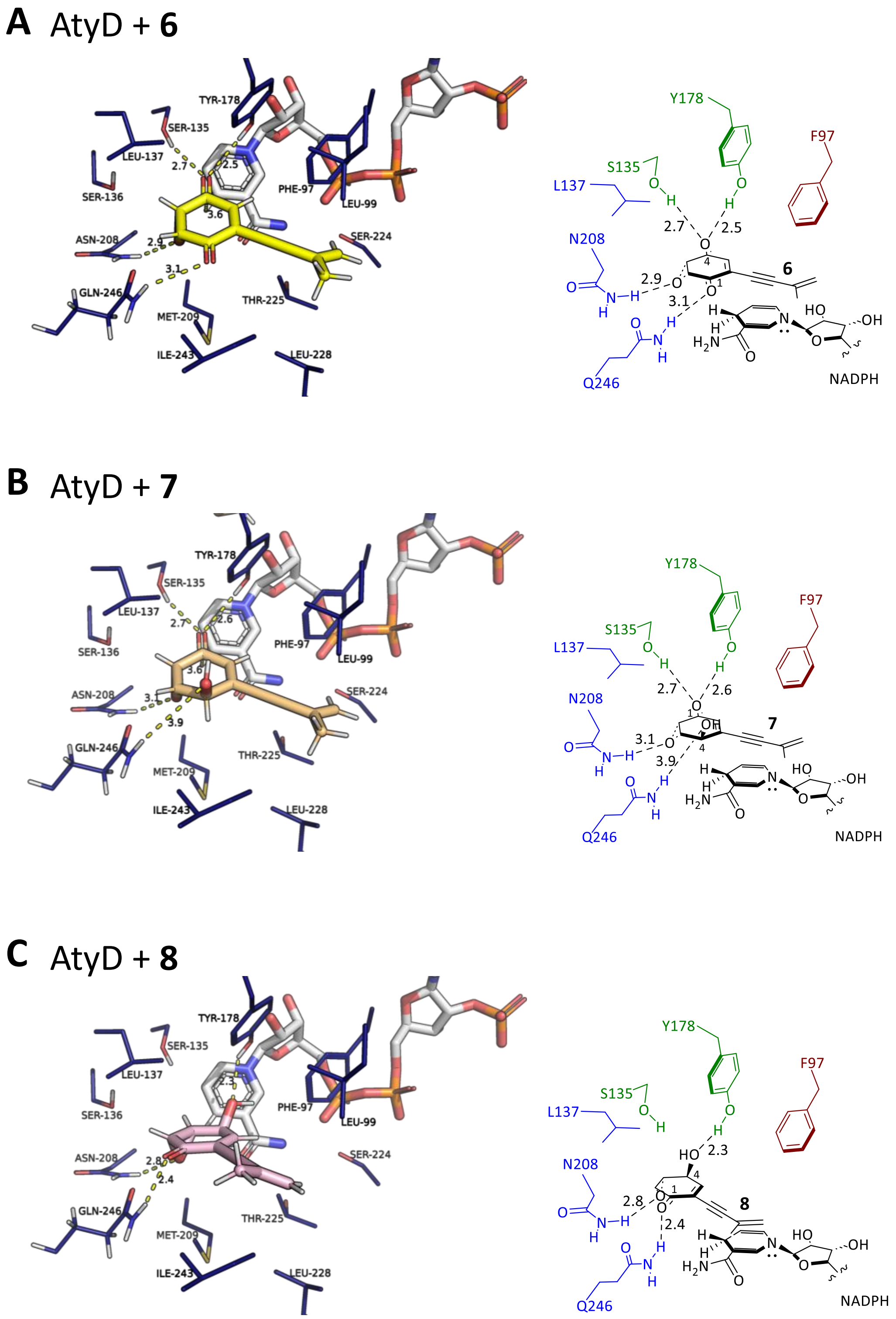

Figure S11. Molecular modeling and docking analysis of AtyD with **6**–**8**.

The left panel shows the predicted binding poses of (A) **6**, (B) **7** and (C) **8** in the catalytic site of AtyD. The right panel represent the 2D scheme of (A) **6**, (B) **7** and (C) **8**, highlighting key interactions with catalytic site residues. The NADPH cofactor is shown in black; catalytic residues are colored green; residues located near the epoxide group are colored blue; and residues located near the 1,3-enyne moiety are colored brown.

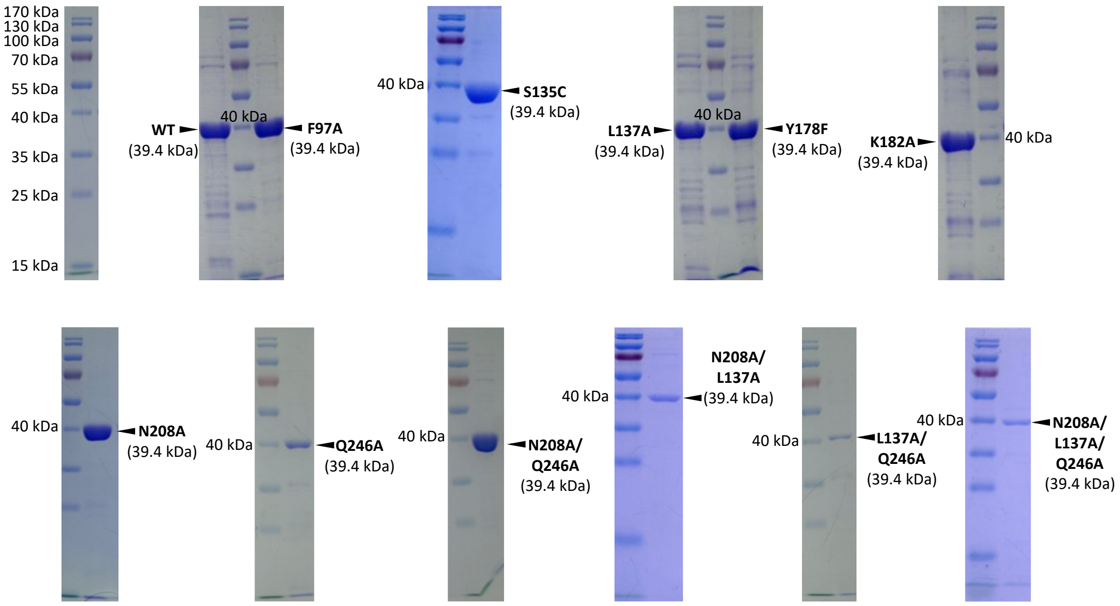

Figure S12. SDS-PAGE analysis of purified AtyD wild-type and mutants (protein size: 39.4 kDa).

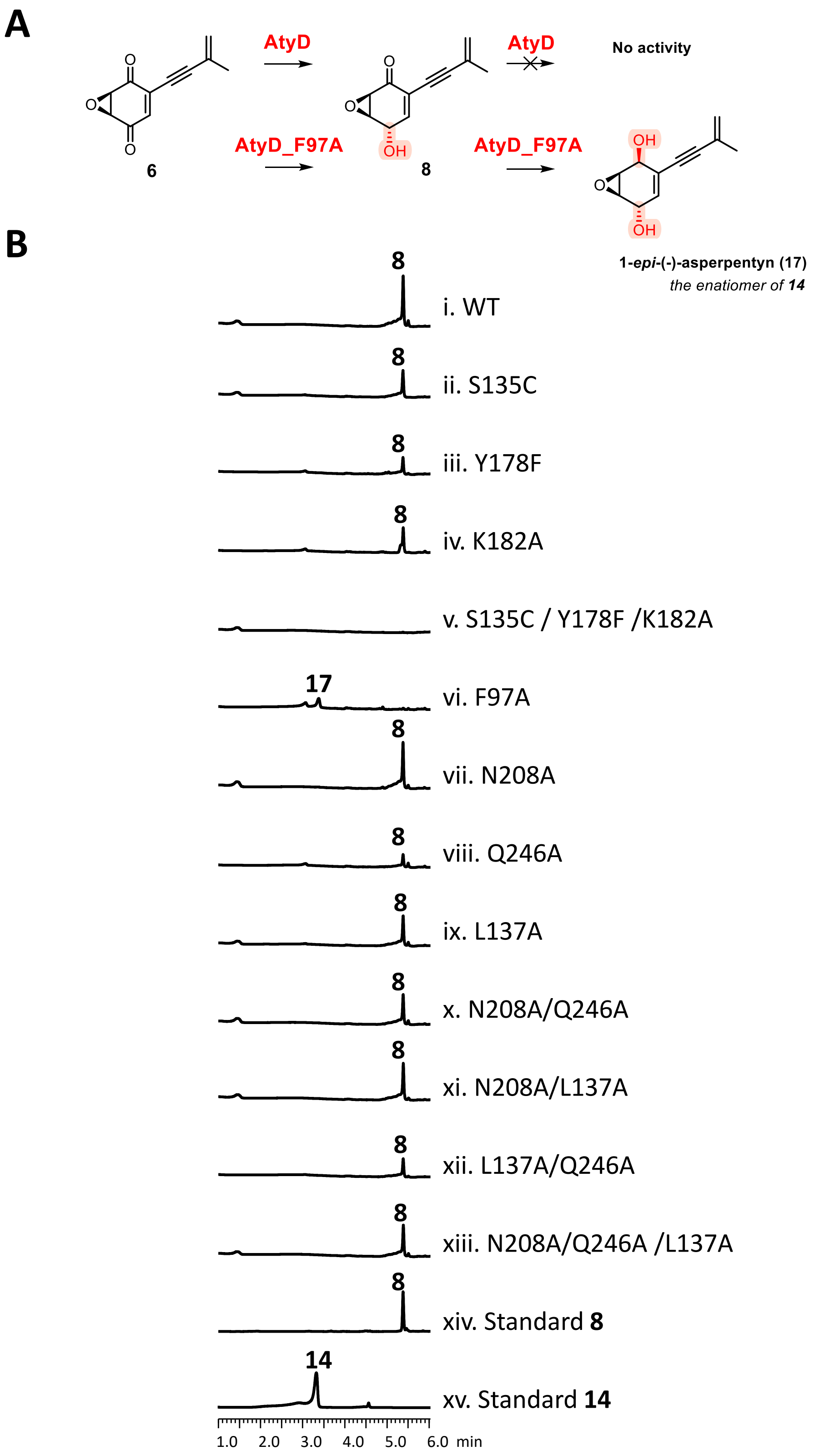

Figure S13. Functional verification of AtyD mutants using substrate **6**.

(A) Chemical reactions catalyzed by AtyD with substrate **6**.

(B) LC-DAD-MS analysis (UV detection at λ = 257 nm) of *in vitro* reactions of wild-type AtyD and mutant variants with substrate **6** and NADPH.

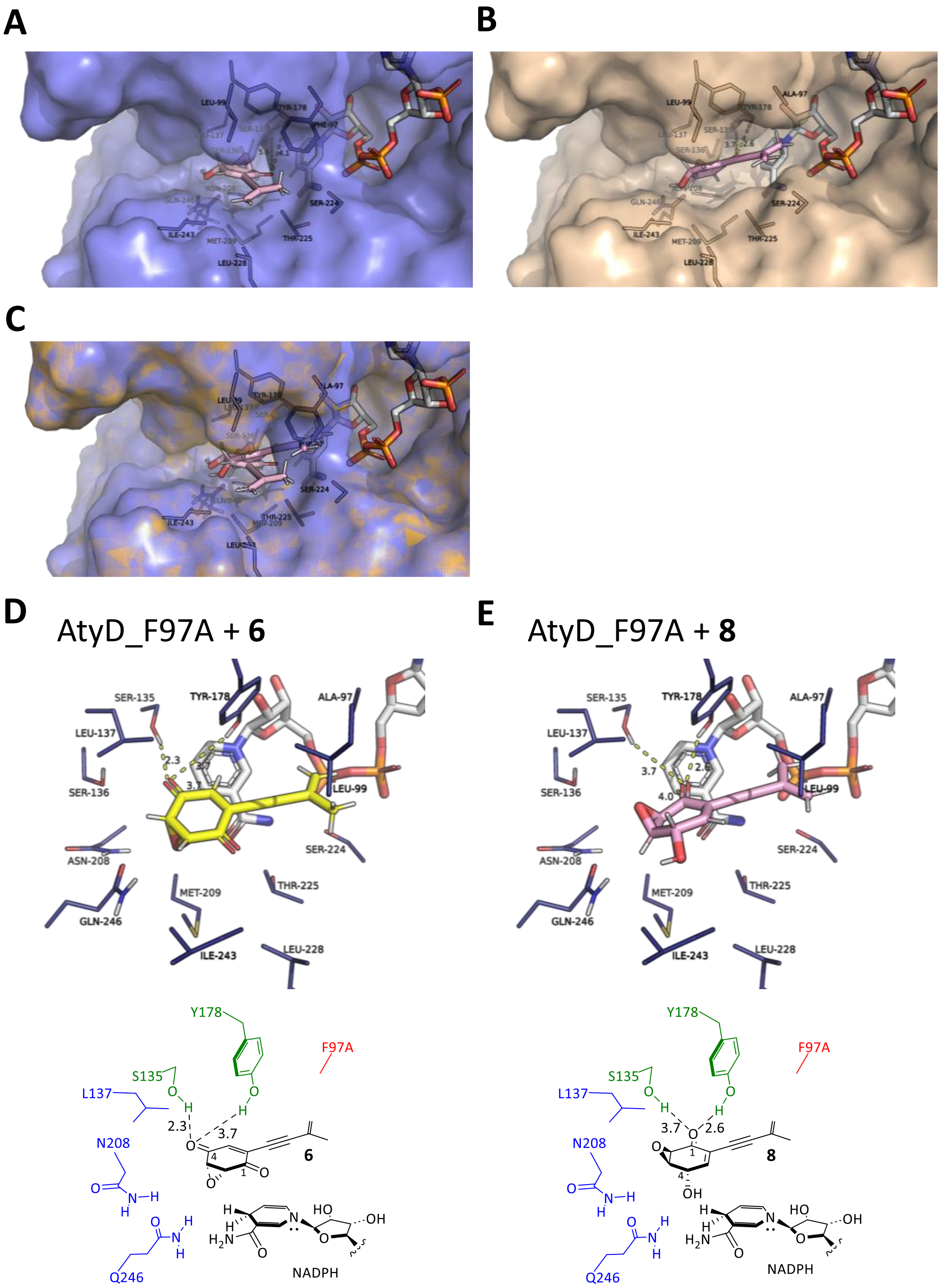

Figure S14. Structural comparison of substrate binding pockets between AtyD and AtyD_F97A mutant with **8.**

The docking model of (A) AtyD with **8**, showing the surface colored in blue and key residues labeled. (B) AtyD_F97A with **8**, showing the surface colored in tan. (C) Superposition of AtyD (blue) and AtyD_F97A (tan) binding pockets. The mutation enlarges the binding pocket and changes substrate orientation. The docking poses of AtyD_F97A with (D) **6** and (E) **8**. The NADPH cofactor is shown in black; catalytic residues are colored green; residues located near the epoxide group are colored blue; and residues located near the 1,3-enyne moiety are colored brown.

A AtyD + **10**

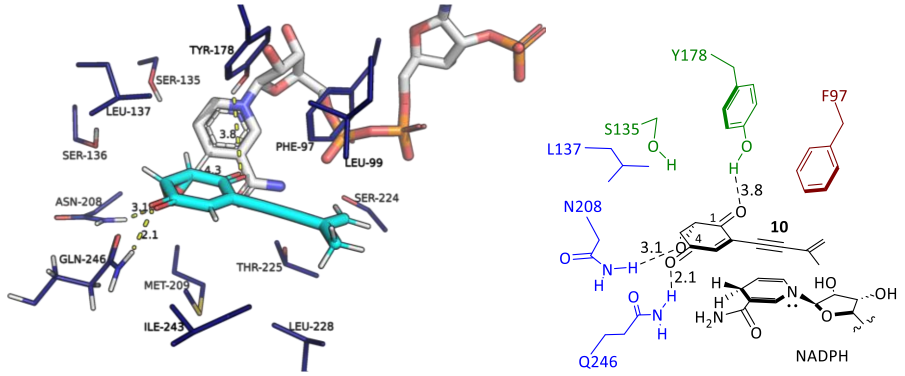

B AtyD + **13**

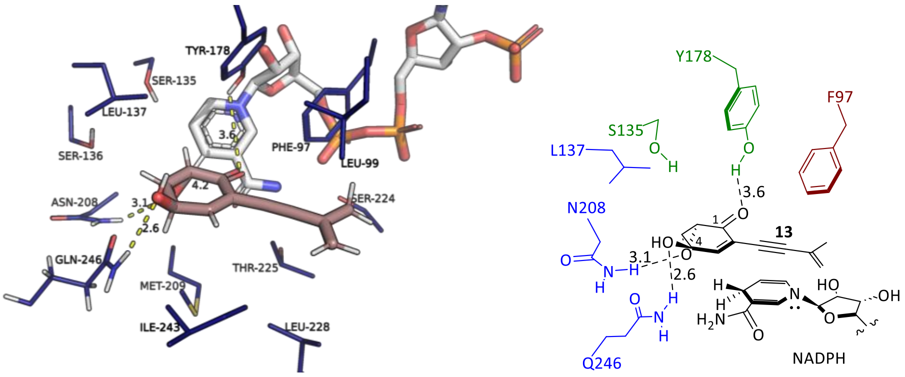

C AtyD + **11**

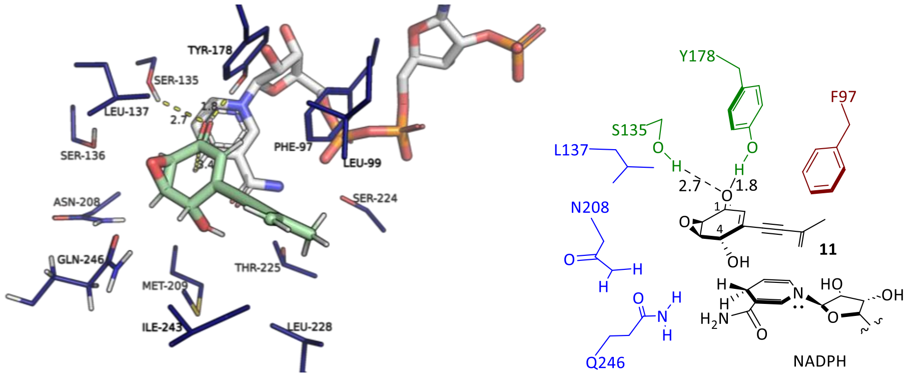

Figure S15. Molecular modeling and docking analysis of AtyD with **10**, **11** and **13.**
The left panel shows the predicted binding poses of (A) **10**, (B) **13** and (C) **11** in the active site of AtyD. The right panel represent the 2D scheme of (A) **10**, (B) **13** and (C) **11**, highlighting key interactions with catalytic site residues. The NADPH cofactor is shown in black; catalytic residues are colored green; residues located near the epoxide group are colored blue; and residues located near the 1,3-enyne moiety are colored brown.

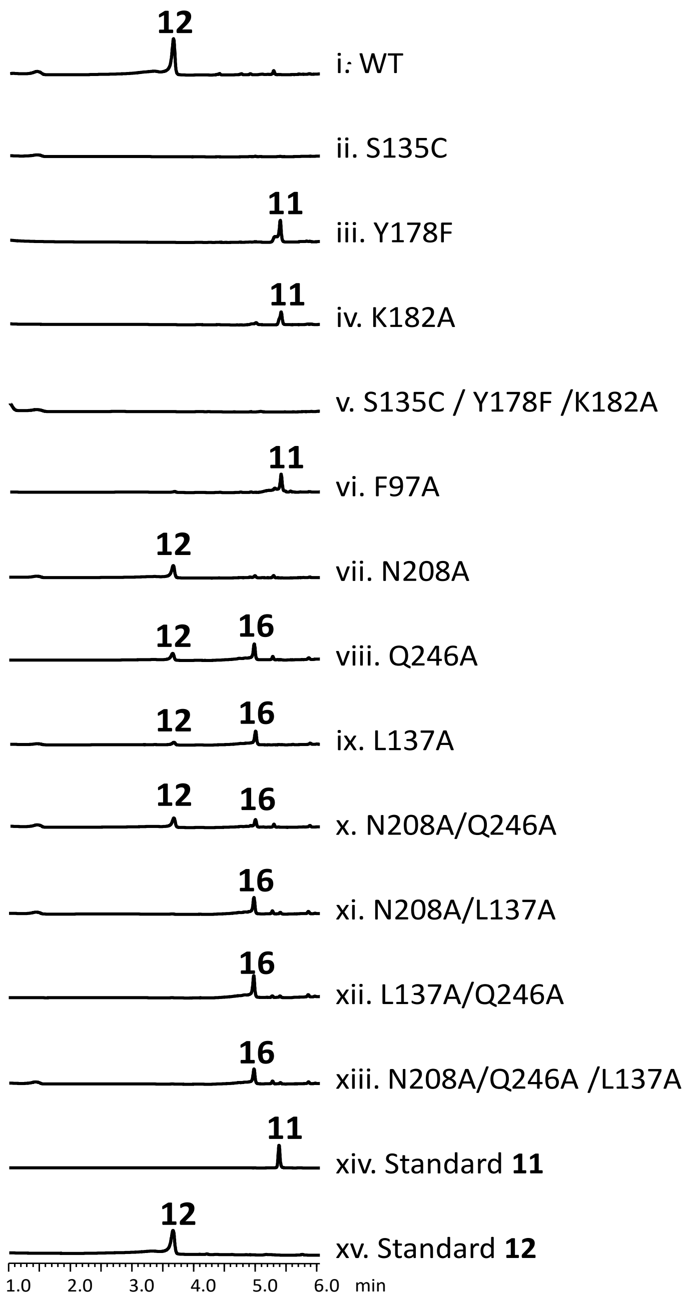

Figure S16. Functional verification of AtyD mutants with 10.

LC-DAD-MS analyses (λ = 257 nm) of *in vitro* reactions of AtyD wild type and mutant variants with NADPH and substrate **10**.

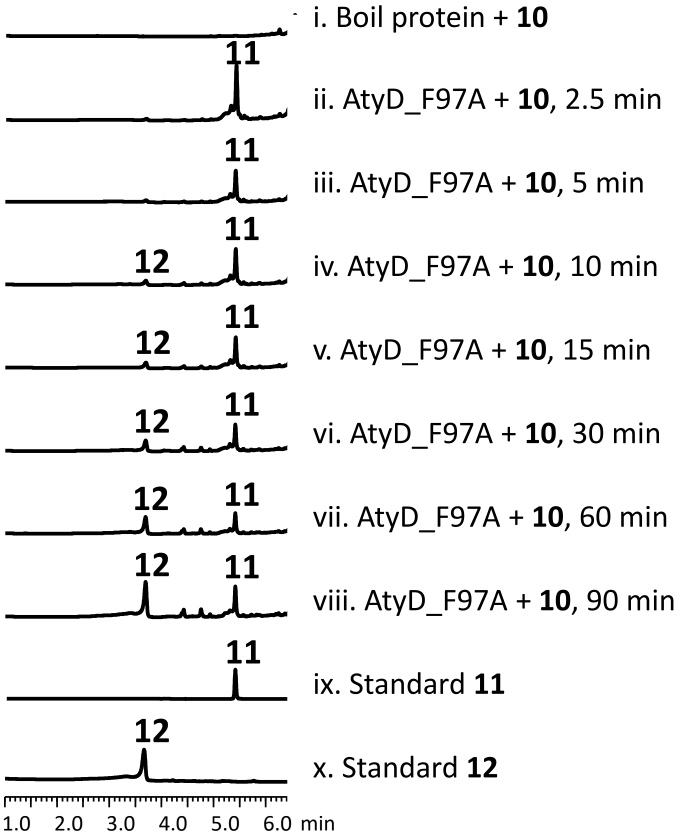

Figure S17. LC-DAD-MS chromatograms of the time-course reaction of AtyD_F97A with NADPH and substrate **10** (UV detection at λ = 257 nm).

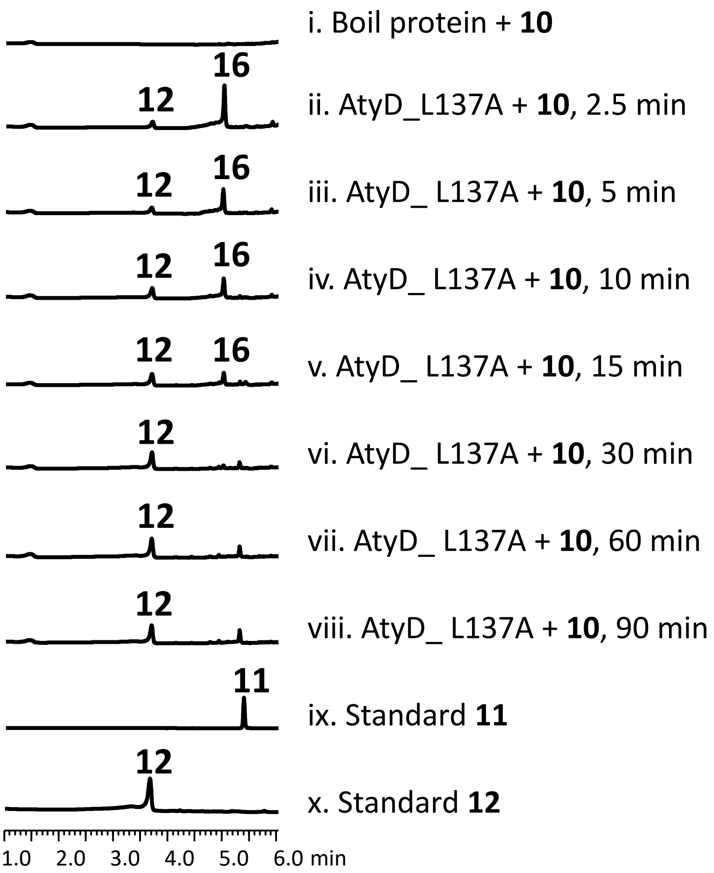

Figure S18. LC-DAD-MS chromatograms of the time-course reaction of AtyD_L137A with NADPH and substrate **10** (UV detection at λ = 257 nm).

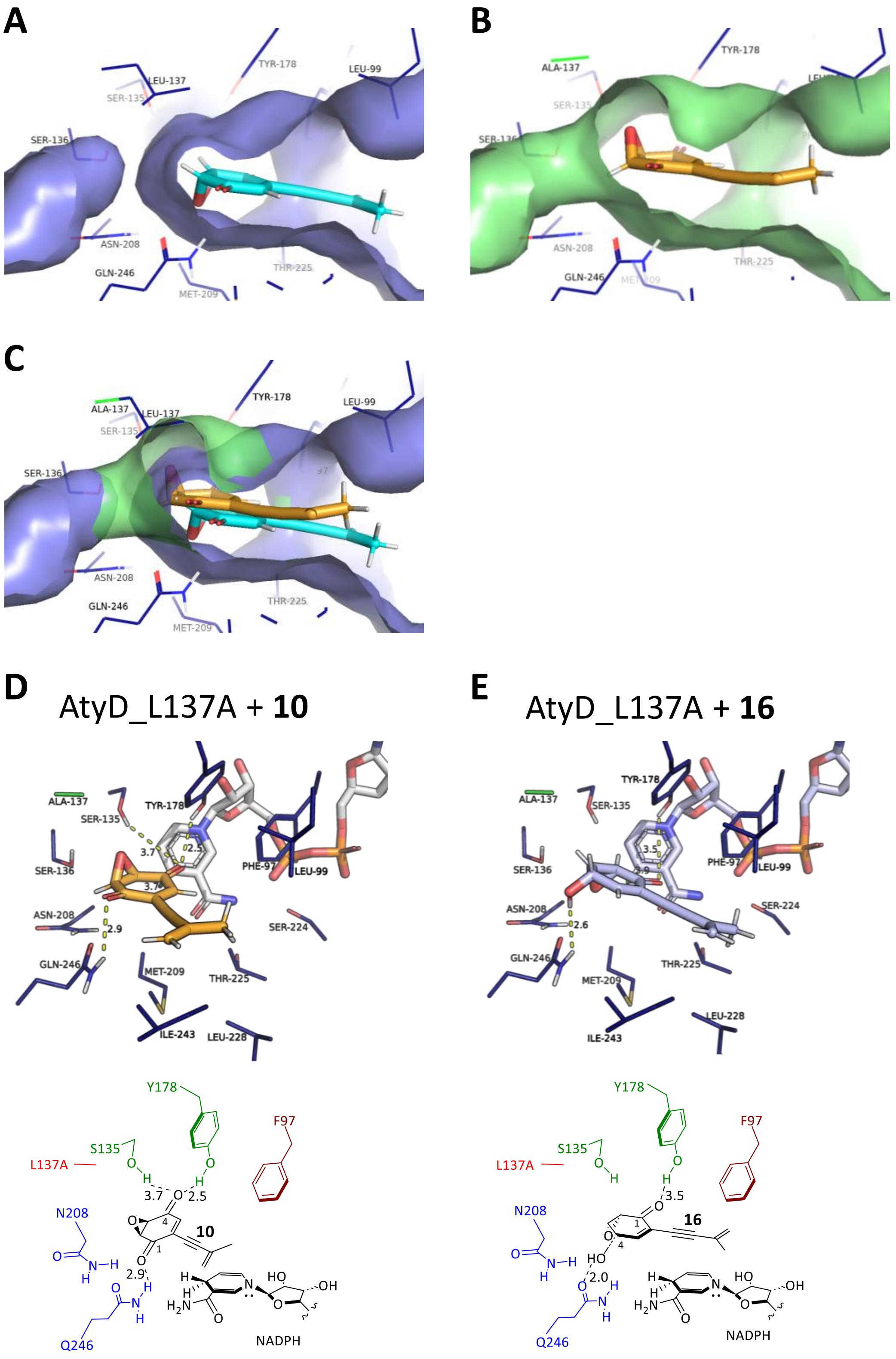

A
B
C
D
AtyD_L137A + 10
E
AtyD_L137A + 16
Y178
S135
L137A
F97
N208
Q246
NADPH

(*continued*)

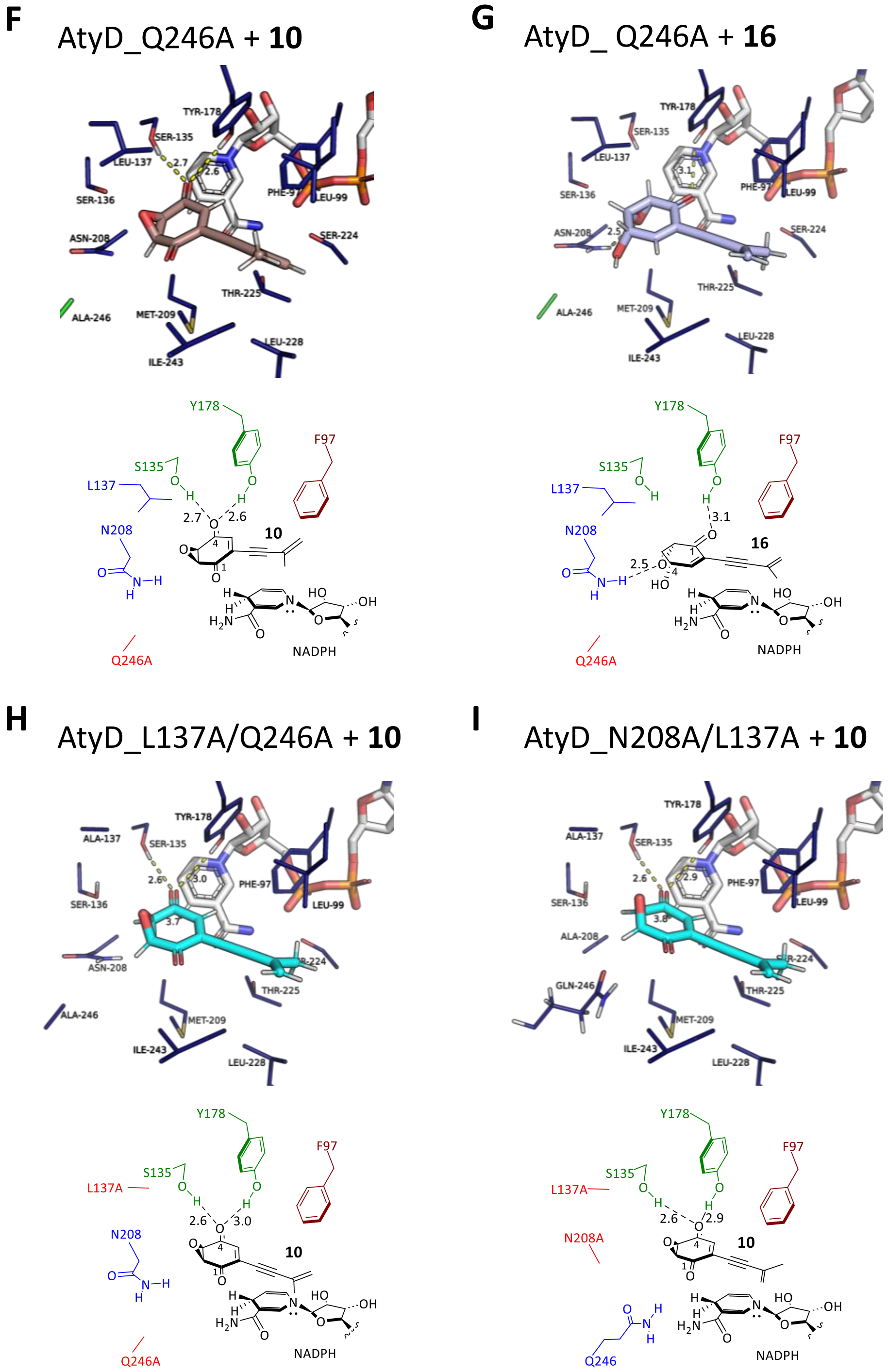

Figure S19. Molecular modeling and docking analysis of AtyD_L137A, AtyD_Q246A, AtyD_L137A/Q246A, and AtyD_N208A/L137A with substrates **10** or **16**. (A–C) Structural comparison of substrate binding pockets between AtyD and AtyD_L137A mutant with **10**. The docking poses of **10** in the catalytic site of (A) AtyD, the surface colored in blue; (B) AtyD_L137A, the surface colored in green; and (C) superposition of AtyD and AtyD_L137A mutant. Docking of AtyD_L137A with (D) **10** and (E) **16**; AtyD_Q246A with (F) **10** and (G) **16**; (H) AtyD_L137A/Q246A with **10** and (I) AtyD_N208A/L137A with **10**. Mutations L137A and Q246A alter active-site geometry and interactions, affecting the regioselectivity of ketoreduction.

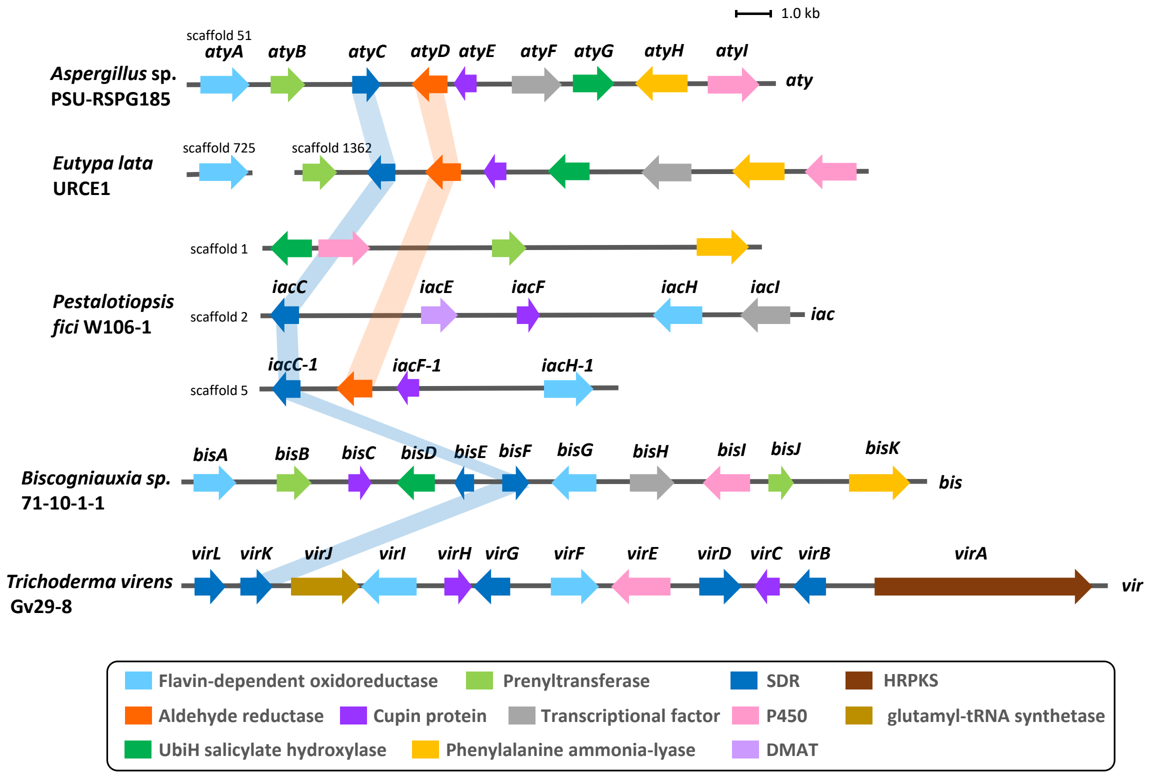

Figure S20. Examples of biosynthetic gene clusters (BGCs) encoding epoxyquinoid natural products that contain AtyC or AtyD homologs.

The *aty* BGC from *Aspergillus sp.* PSU-RSPG185, the BGC from *Eutypa lata* URCE1 (that produces eutypoxide B), the *iac* BGC from *Pestalotiopsis fici* W106-1 (that produces iso-A82775C), the *bis* BGC from *Biscogniauxia sp*. (that produces biscognienyne B), and the *vir* BGC from *Trichoderma virens* Gv29-8 (that produces trichoxide) harbor AtyC or AtyD homologs. AtyC shares amino acid sequence identity to *E. lata*-AtyC (EMR66150.1), IacC (XP_007830811), IacC-1 (XP_007834716), BisF (QJQ82460.1) and VirK (XP_013952628.1) with 70%, 64%, 70%, 69% and 53%, respectively. AtyD shares amino acid sequence identity to Iac-AtyD (XP_007834717.1) and *E. lata*-AtyD (EMR66145.1) with 70%, and 69%, respectively.

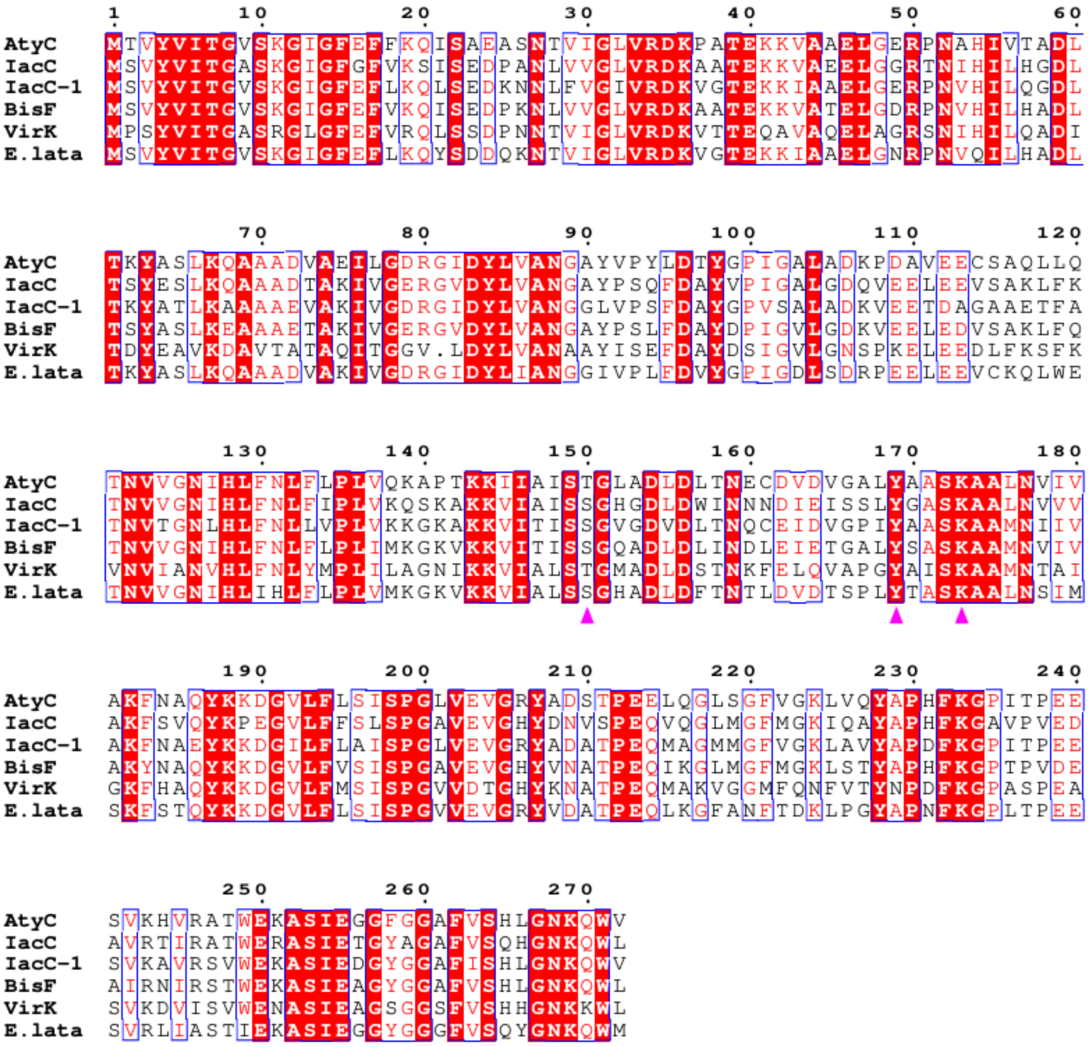

Figure S21. Protein sequence alignment of AtyC with homologous proteins.

Protein sequence alignment of AtyC with homologous short-chain dehydrogenases (SDRs). Homologs include *E. lata*-AtyC (EMR66150.1), IacC (XP_007830811), IacC-1 (XP_007834716), BisF (QJQ82460.1), and VirK (XP_013952628.1). The conserved catalytic residues—Threonine (or Serine), Tyrosine, and Lysine (corresponding to Thr150, Tyr169, and Lys173 in AtyC)—were identified by alignment with a carbonyl reductase from the classical SDR family (accession no. cd05325) and are indicated with pink triangles.

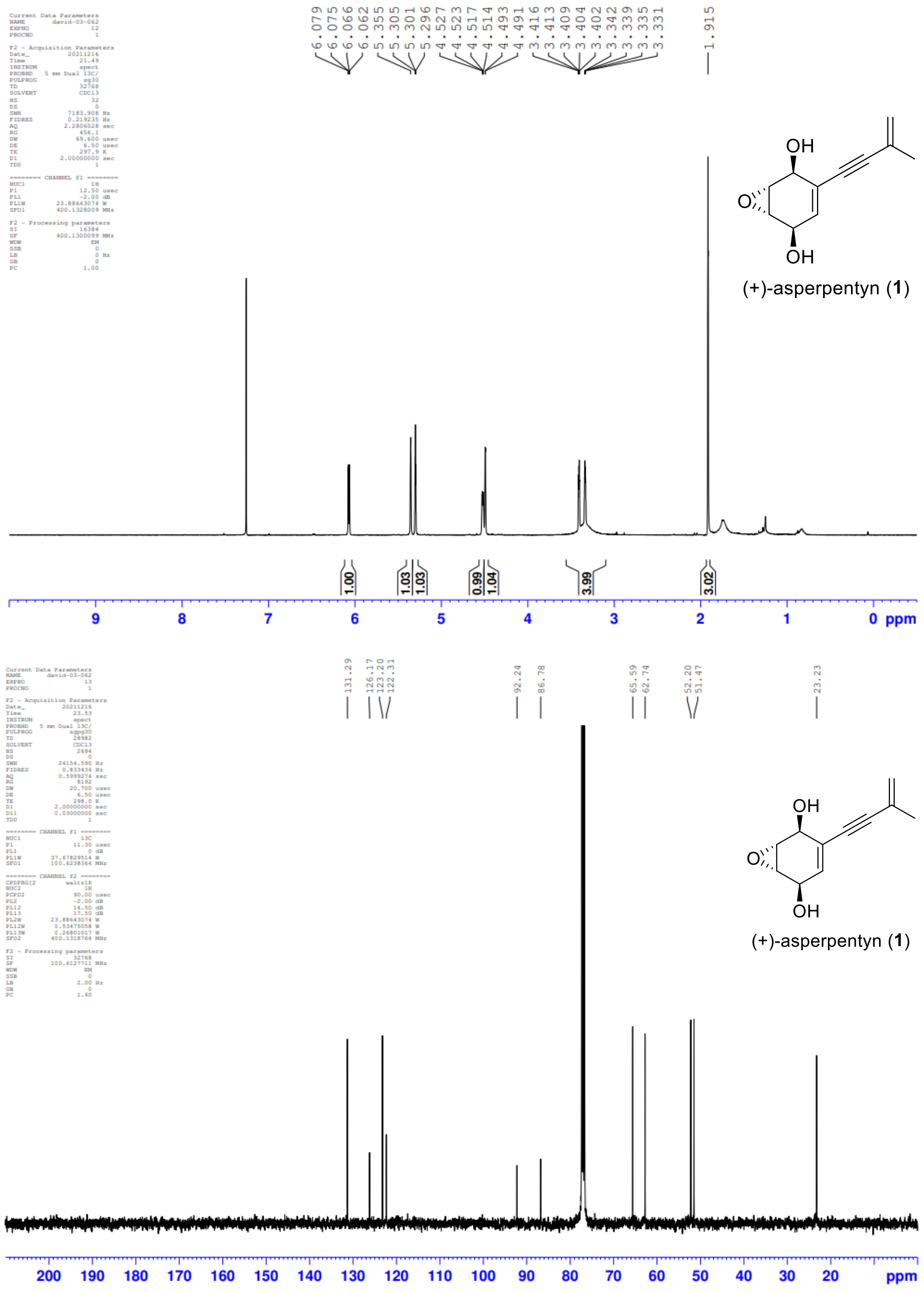

Figure S22. ^1H NMR spectrum ($CDCl_3$, 500 MHz) and ^{13}C NMR ($CDCl_3$, 125 MHz) of (+)-asperpentyn (**1**).

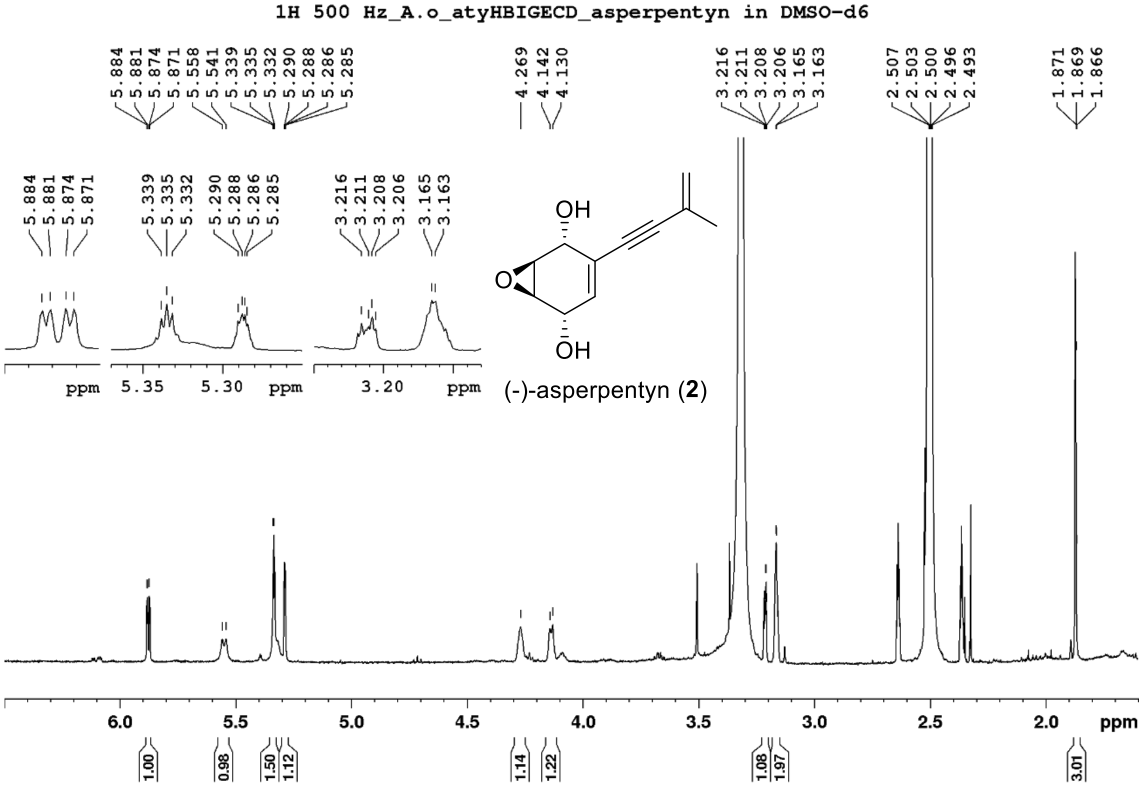

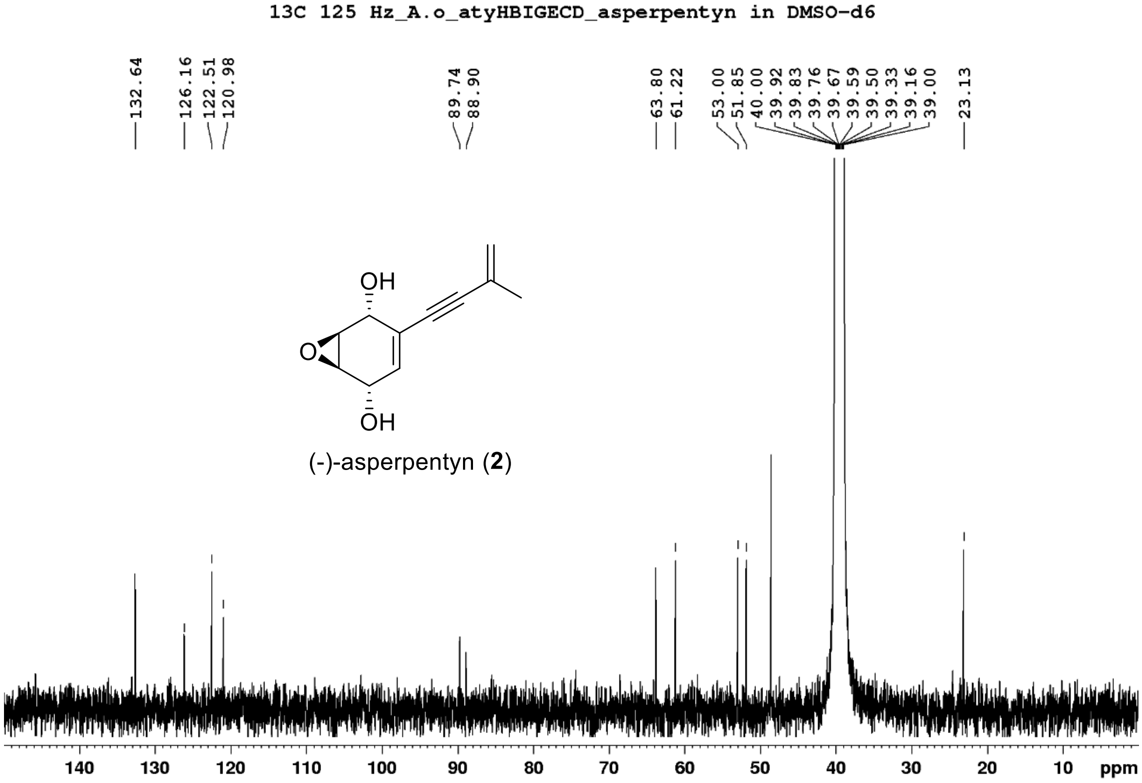

Figure S23. ^{1}H NMR spectrum (DMSO-d_6, 500 MHz) and ^{13}C NMR (DMSO-d_6, 125 MHz) of (-)-asperpentyn (**2**).

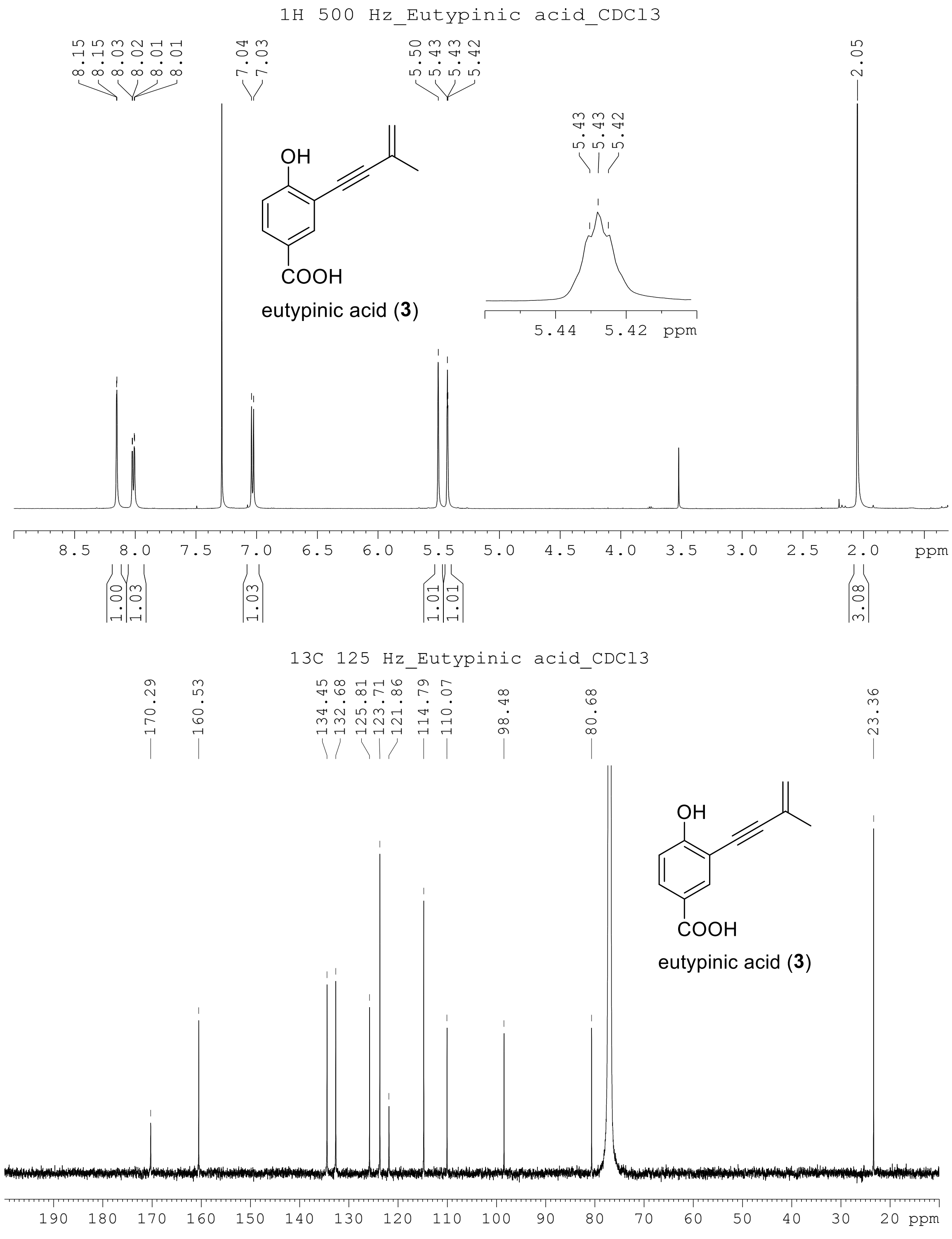

Figure S24. ^{1}H NMR spectrum ($CDCl_3$, 500 MHz) and ^{13}C NMR ($CDCl_3$, 125 MHz) of eutypinic acid (**3**).

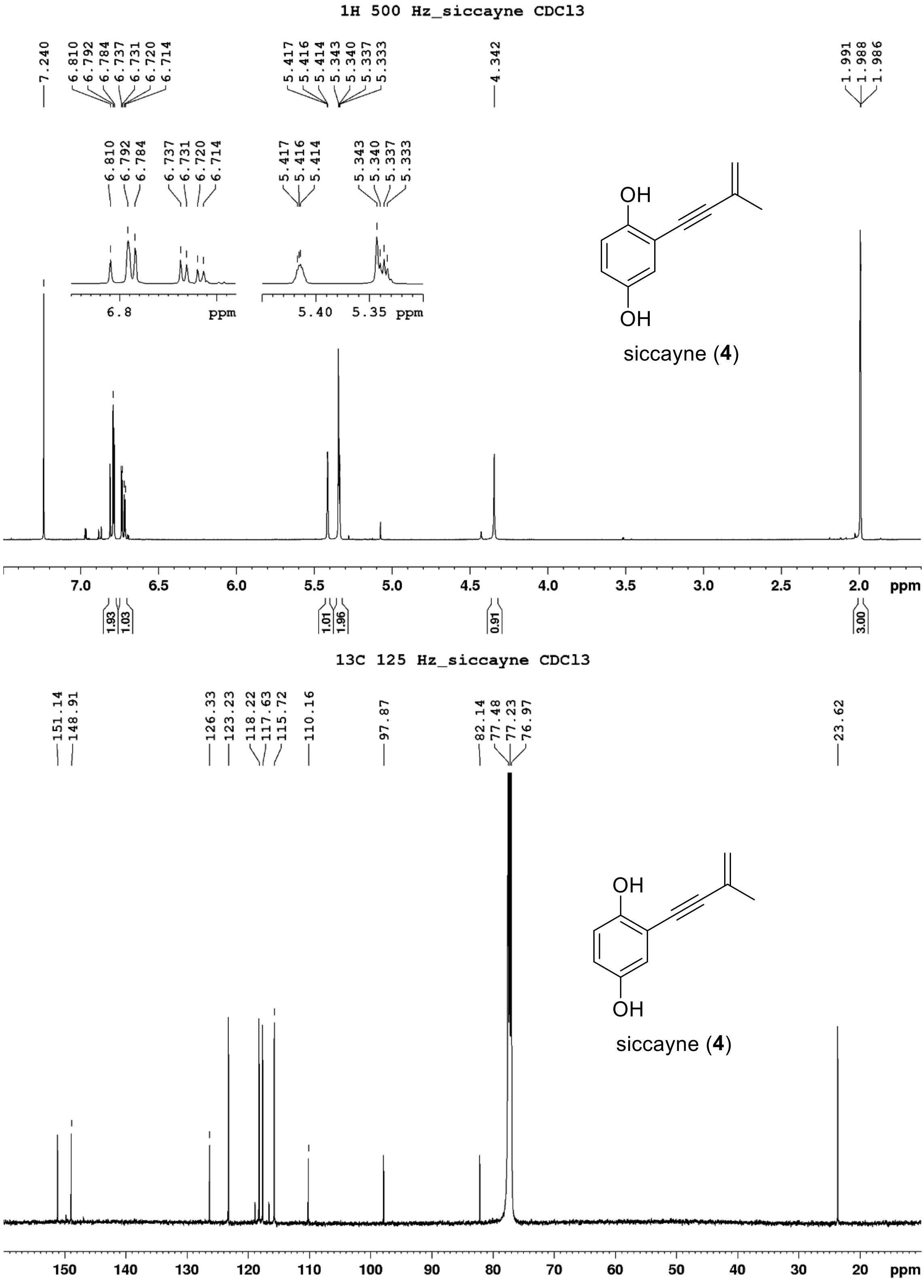

Figure S25. ^{1}H NMR spectrum ($CDCl_3$, 500 MHz) and ^{13}C NMR ($CDCl_3$, 125 MHz) of siccayne (**4**).

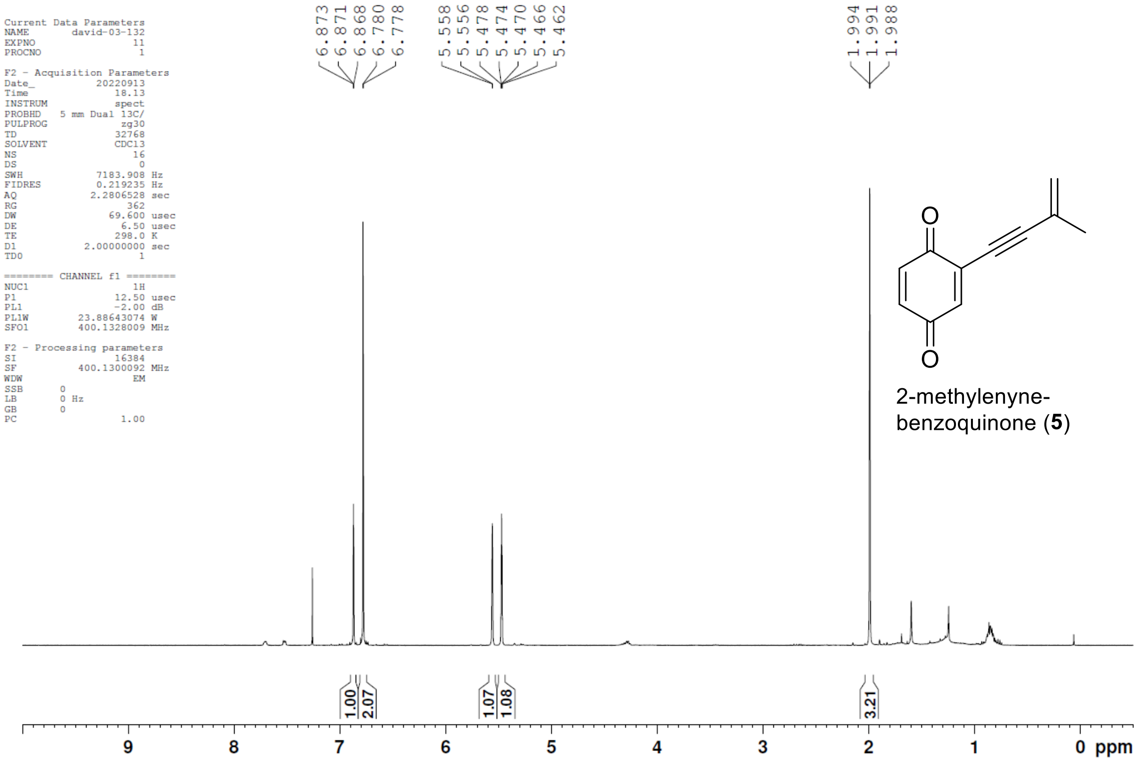

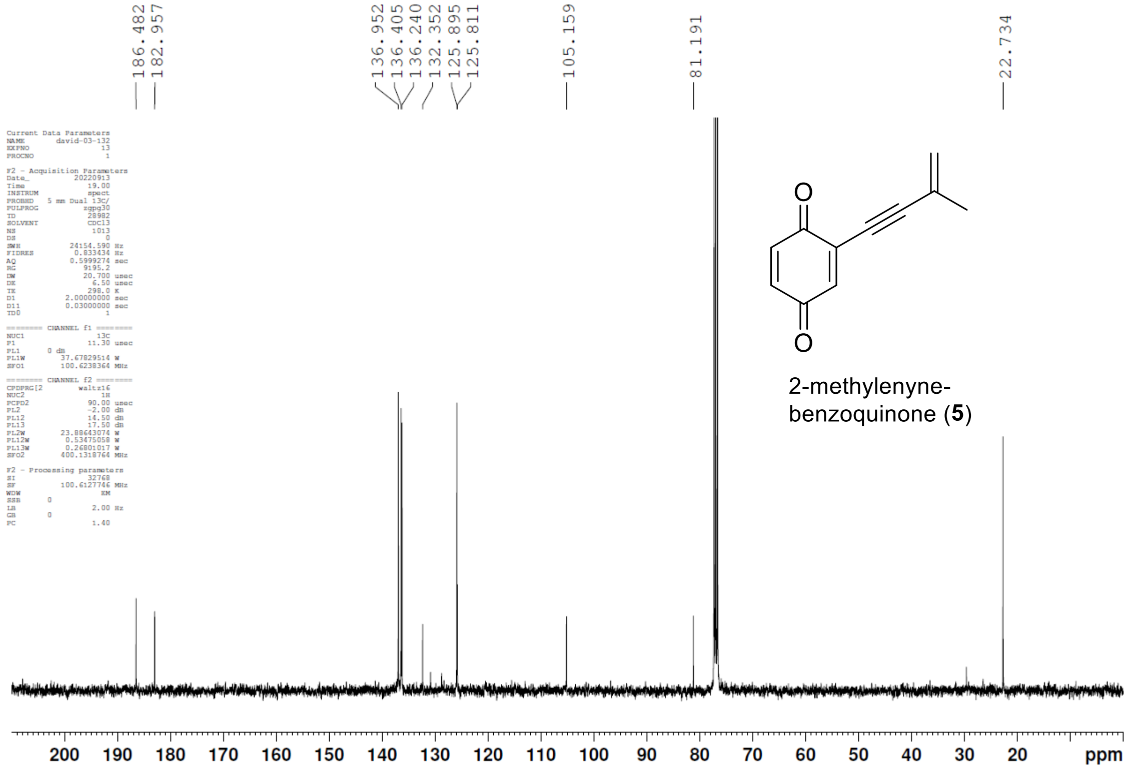

Figure S26. ^{1}H NMR spectrum ($CDCl_3$, 500 MHz) and ^{13}C NMR ($CDCl_3$,125 MHz) of 2-methylenyne-benzoquinone (**5**).

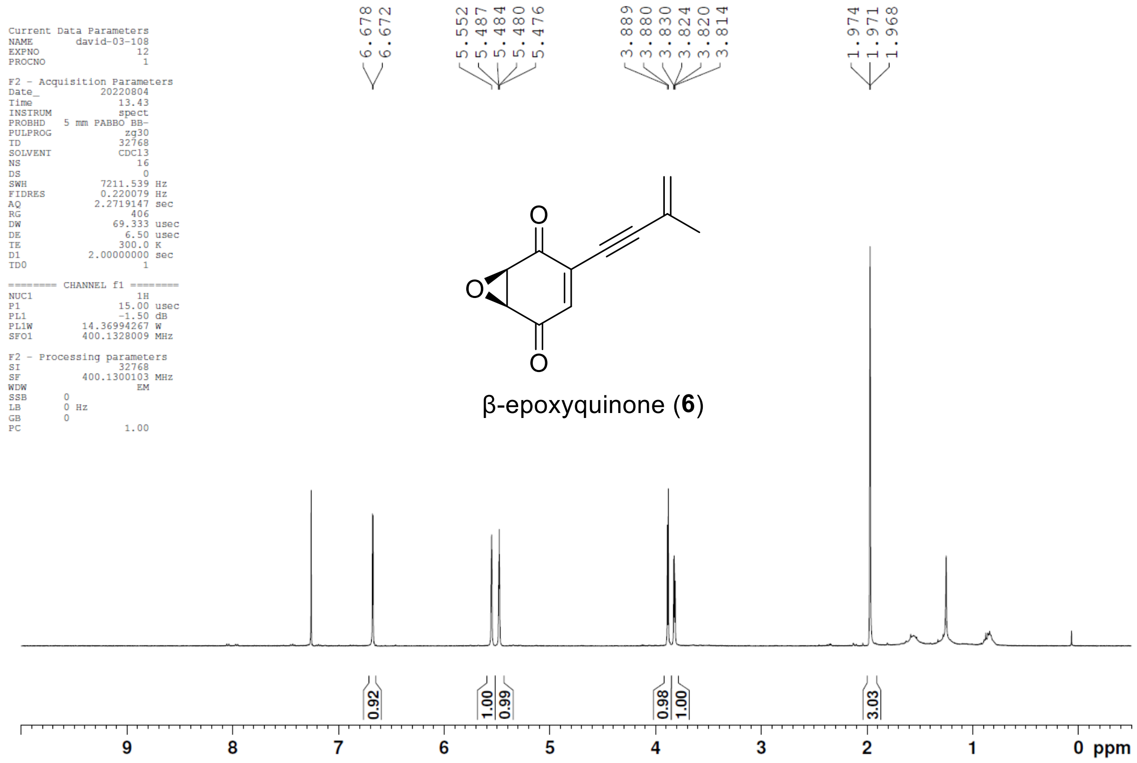

Figure S27. ^{1}H NMR spectrum ($CDCl_3$, 500 MHz) of (2*R*,3*S*)-β-epoxyquinone (**6**).

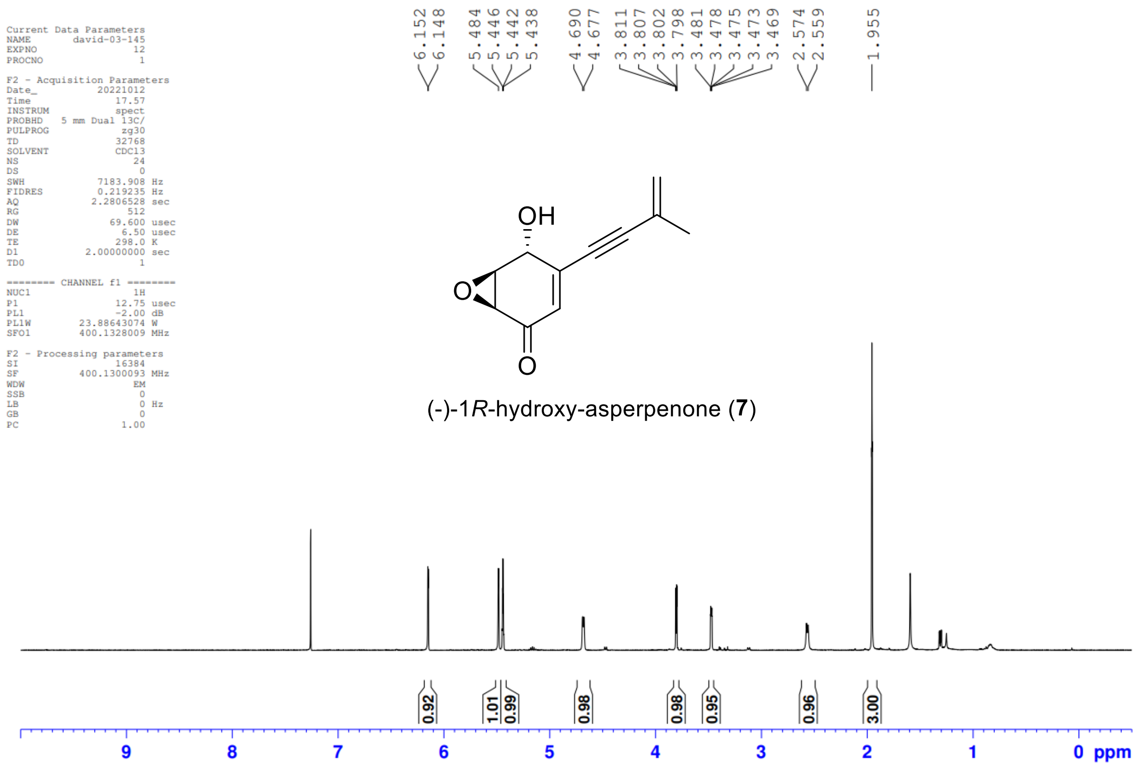

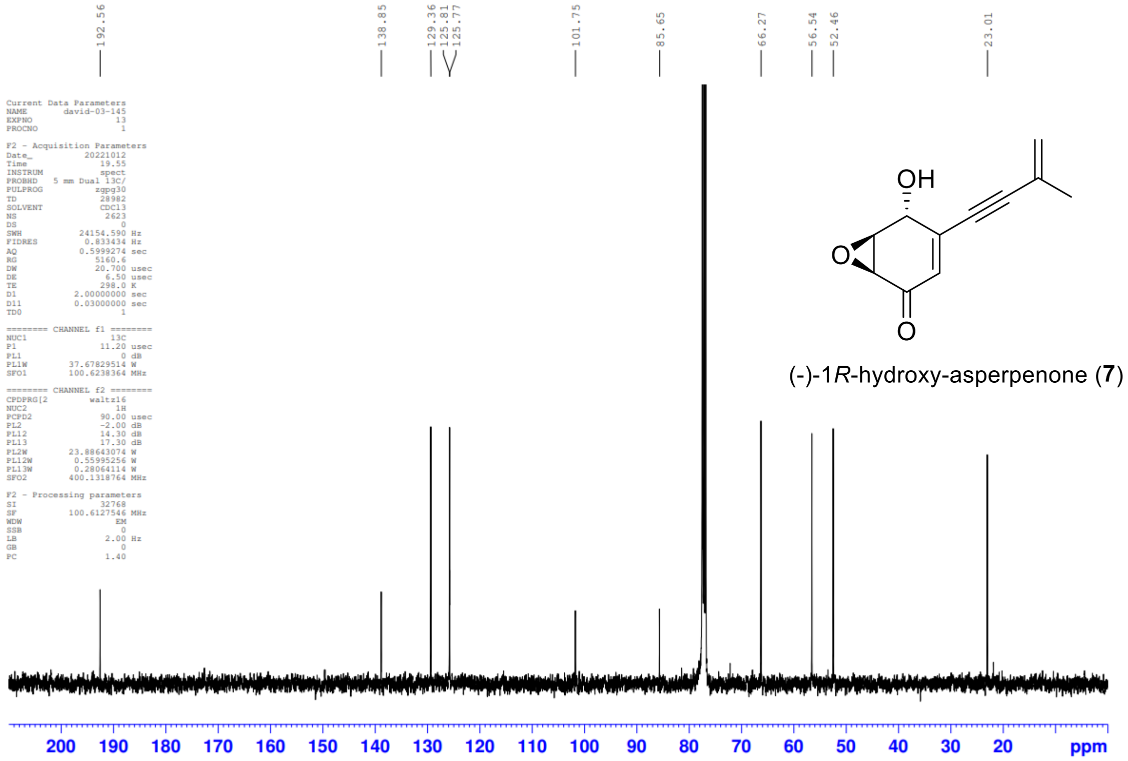

Figure S28. ^1H NMR spectrum ($CDCl_3$, 500 MHz) and ^{13}C NMR ($CDCl_3$, 125 MHz) of (-)-1*R*-hydroxy-asperpenone (**7**).

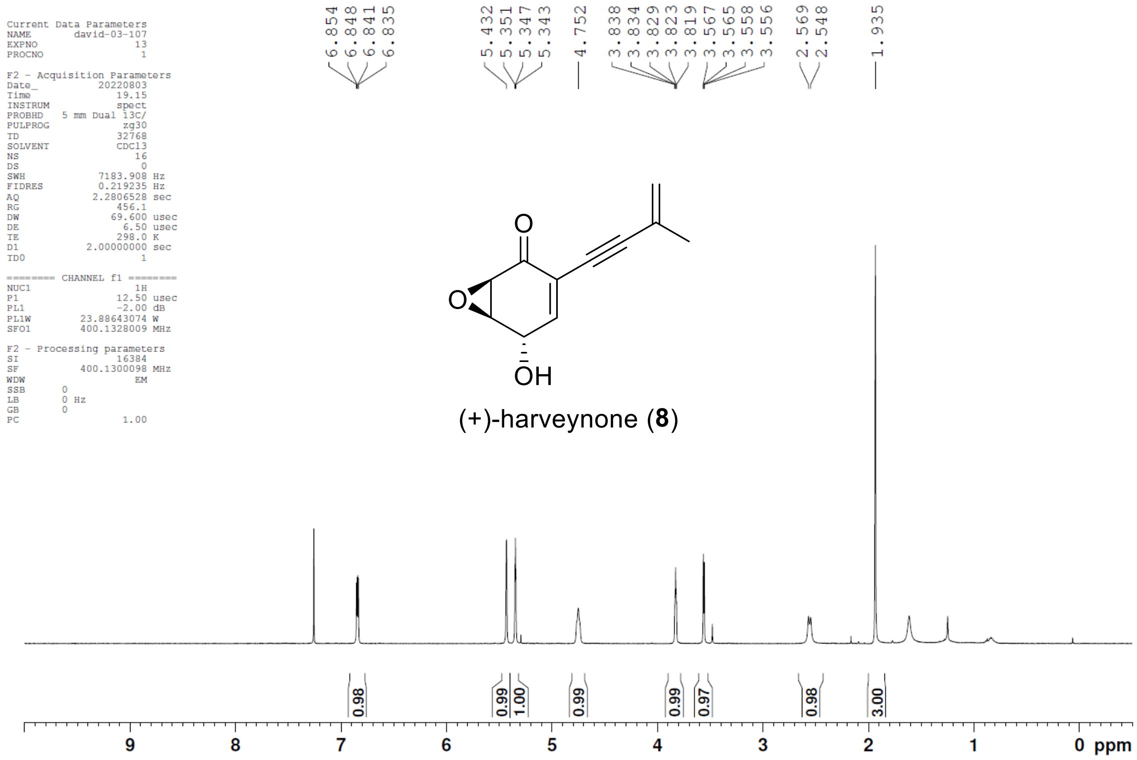

Figure S29. ^{1}H NMR spectrum ($CDCl_3$, 500 MHz) of (+)-harveynone (**8**).

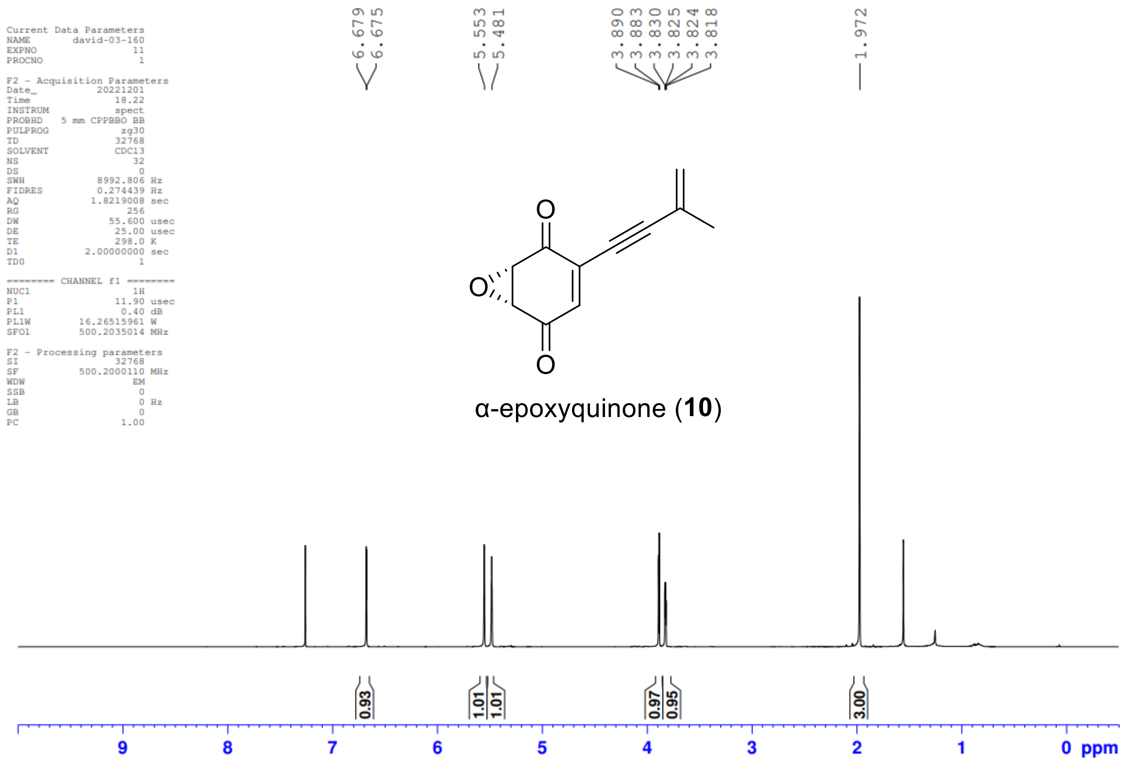

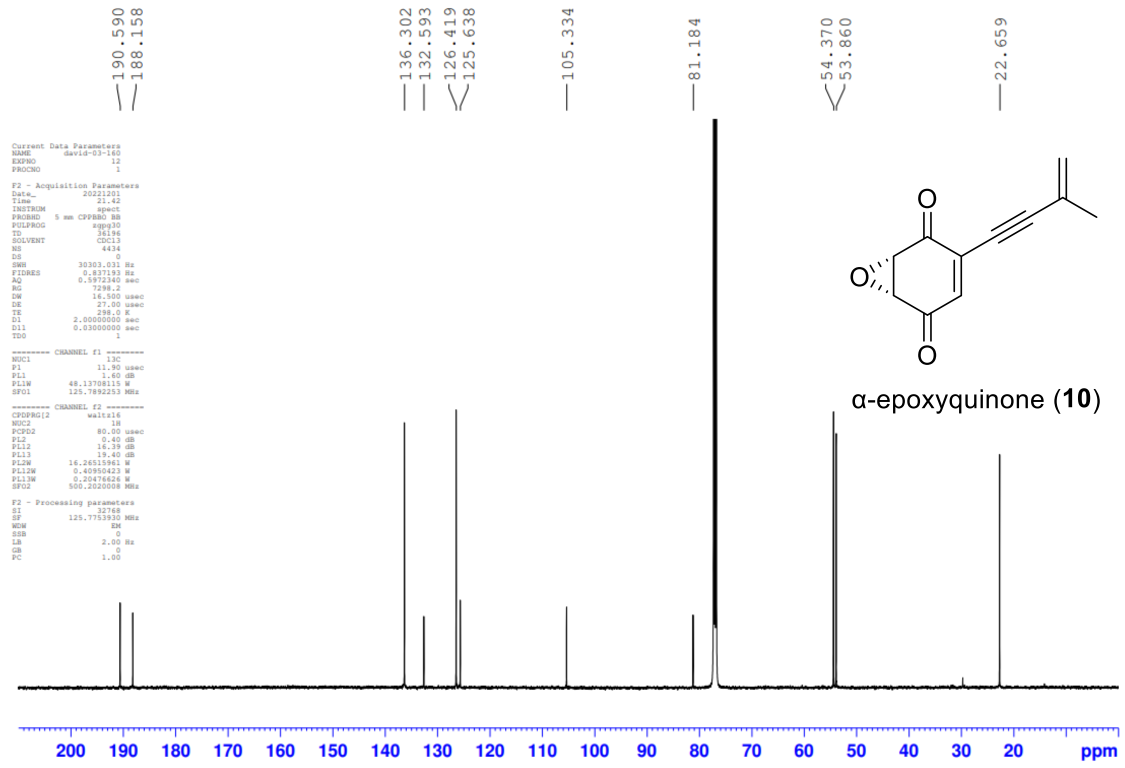

Figure S30. ^{1}H NMR spectrum ($CDCl_3$, 500 MHz) and ^{13}C NMR ($CDCl_3$, 125 MHz) of (2*S*,3*R*)-α-epoxyquinone (**10**).

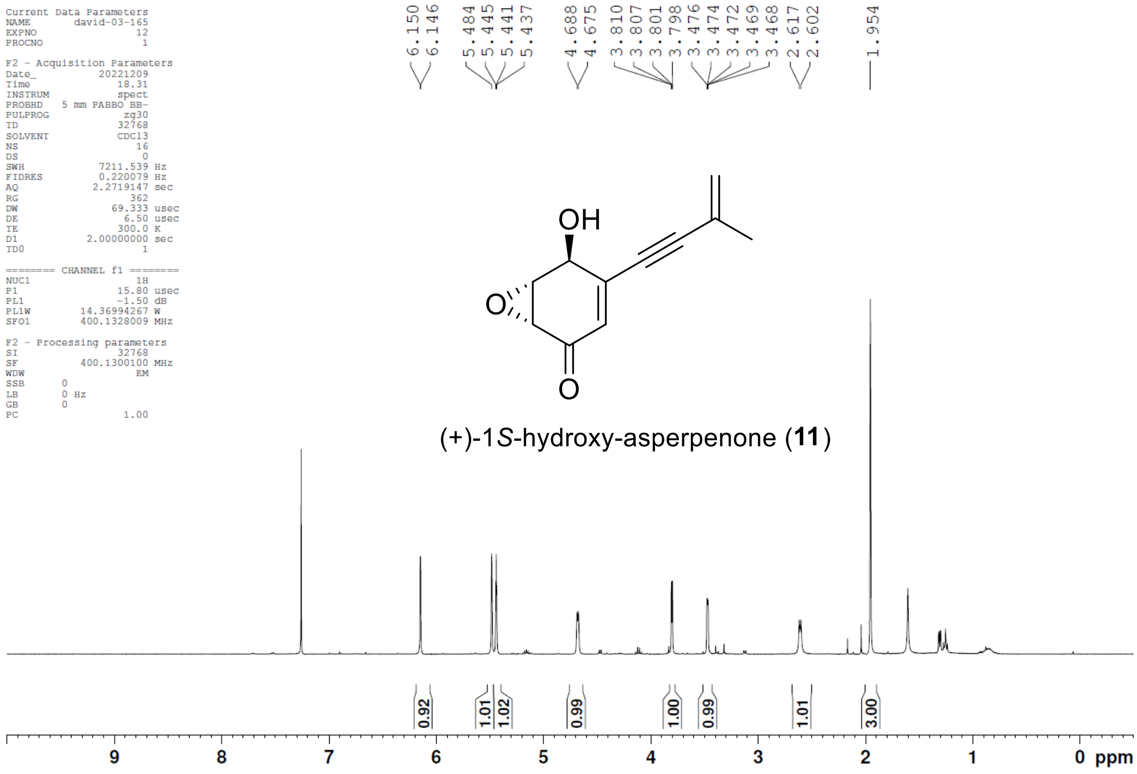

Figure S31. ^{1}H NMR spectrum ($CDCl_3$, 500 MHz) of (+)-1*S*-hydroxy-asperpenone (**11**).

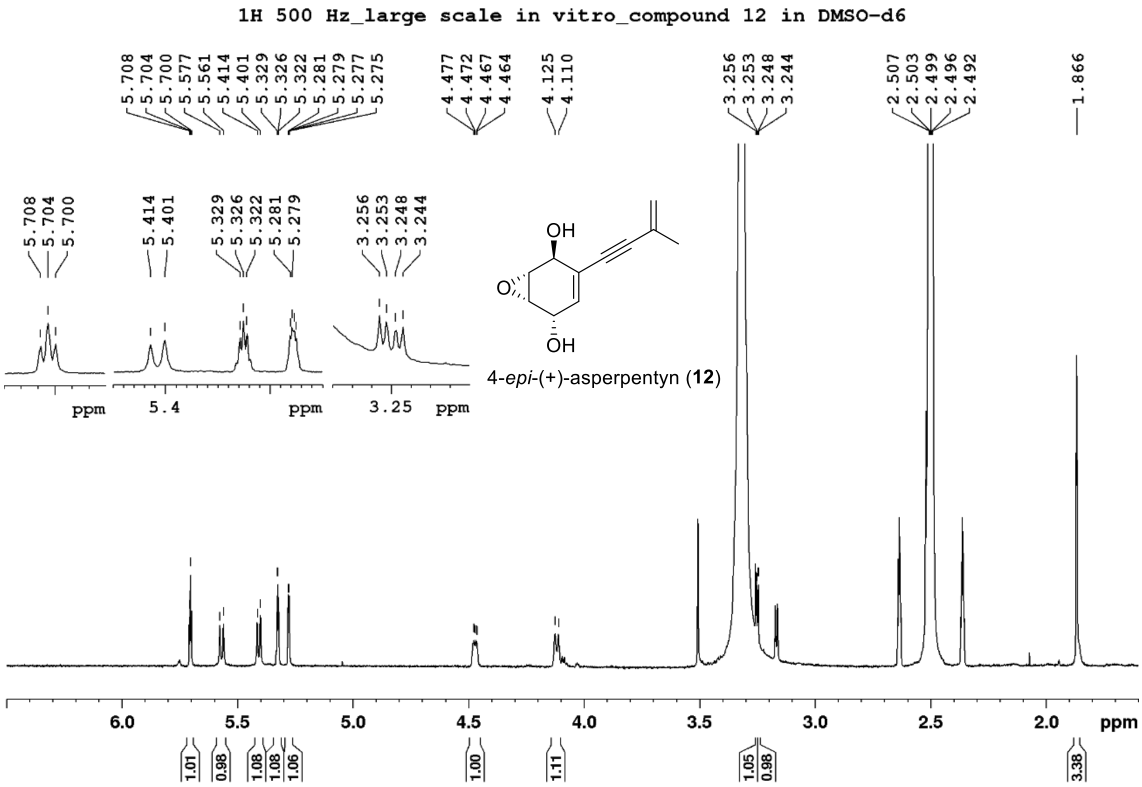

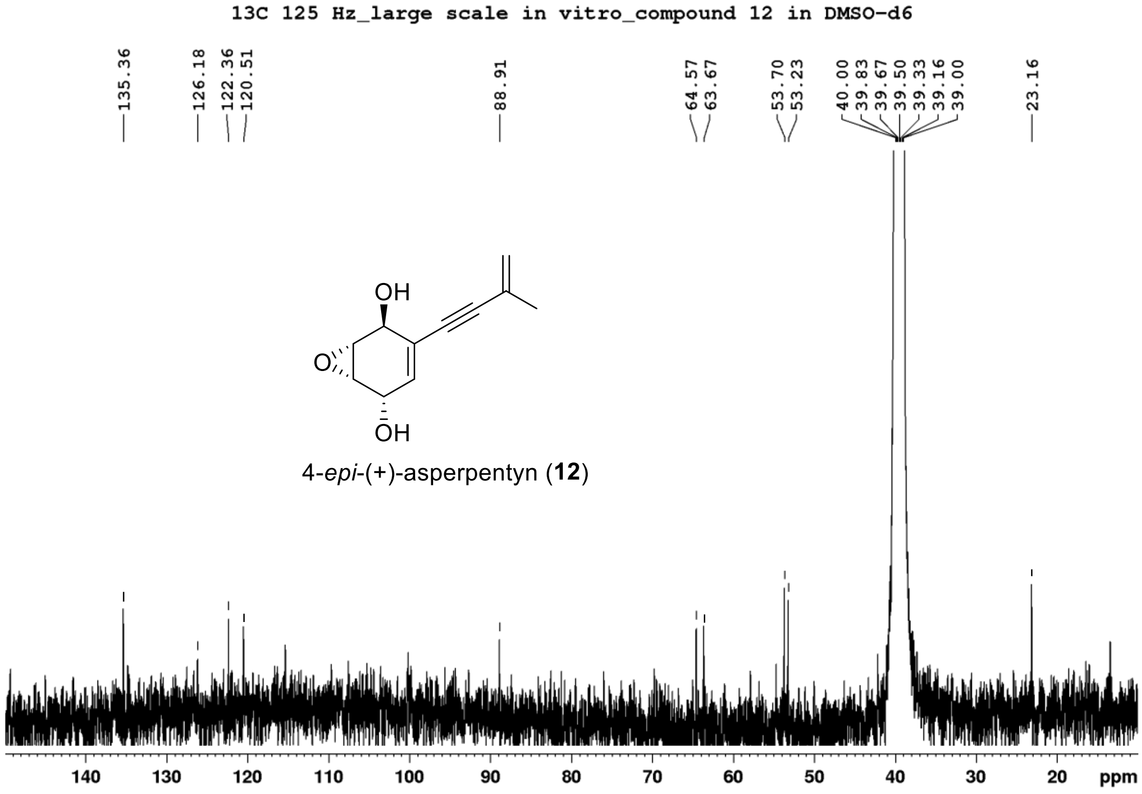

Figure S32. ^{1}H NMR spectrum (DMSO-d$_6$, 500 MHz) and ^{13}C NMR (DMSO-d$_6$, 125 MHz) of 4-*epi*-(+)-asperpentyn (**12**).

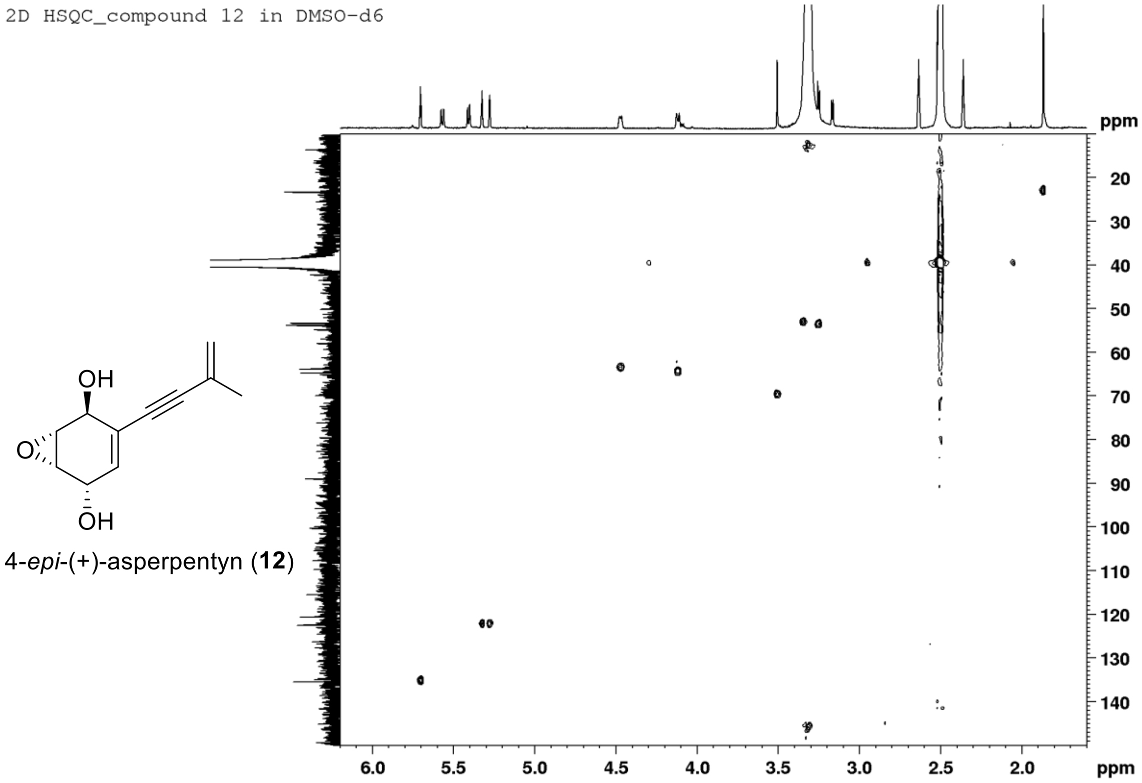

Figure S33. HSQC spectrum (DMSO-d_6, 500 MHz) of 4-*epi*-(+)-asperpentyn (**12**).

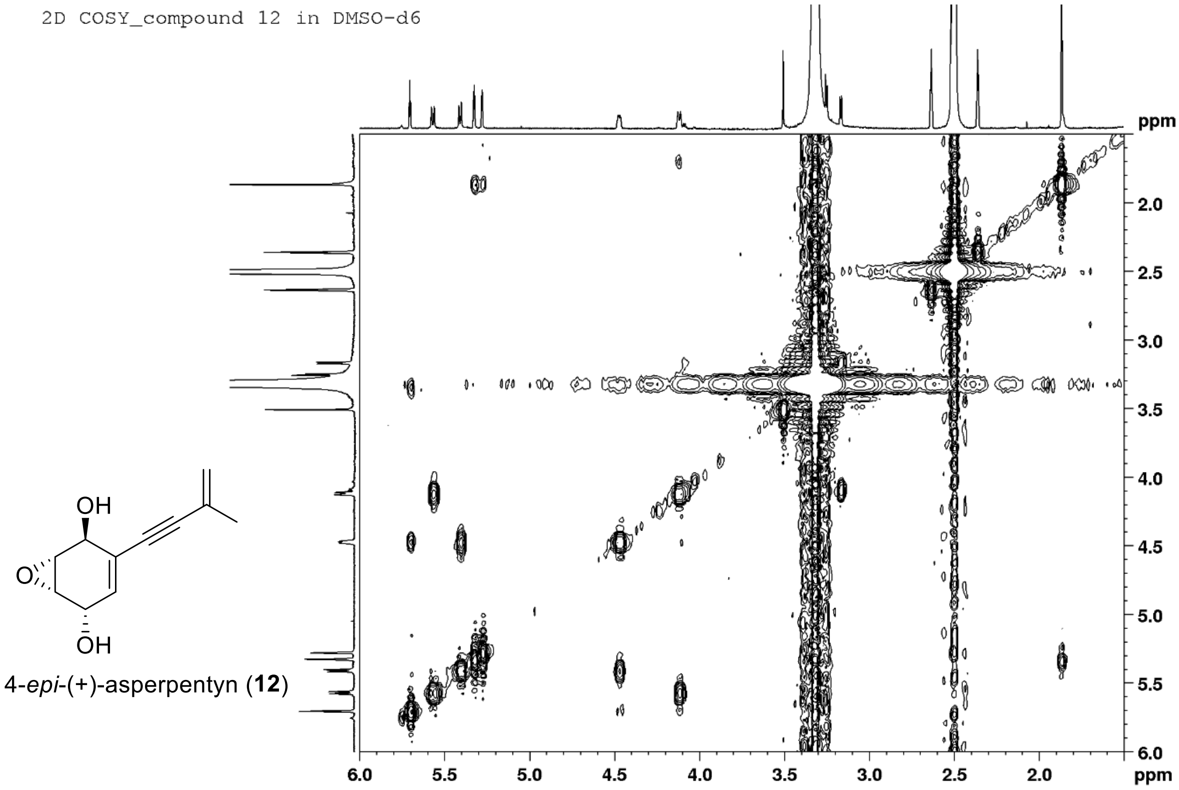

Figure S34. COSY spectrum (DMSO-d_6, 500 MHz) of 4-*epi*-(+)-asperpentyn (**12**).

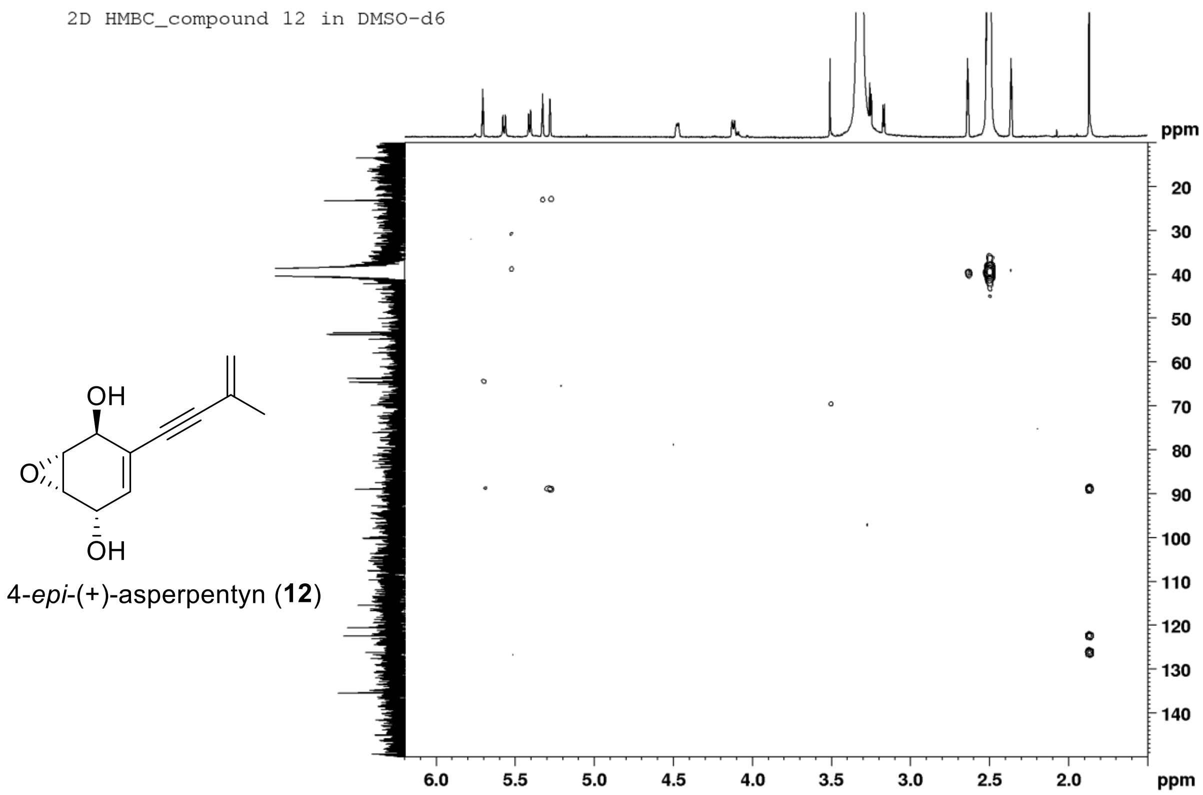

Figure S35. HMBC spectrum (DMSO-d_6, 500 MHz) of 4-*epi*-(+)-asperpentyn (**12**).

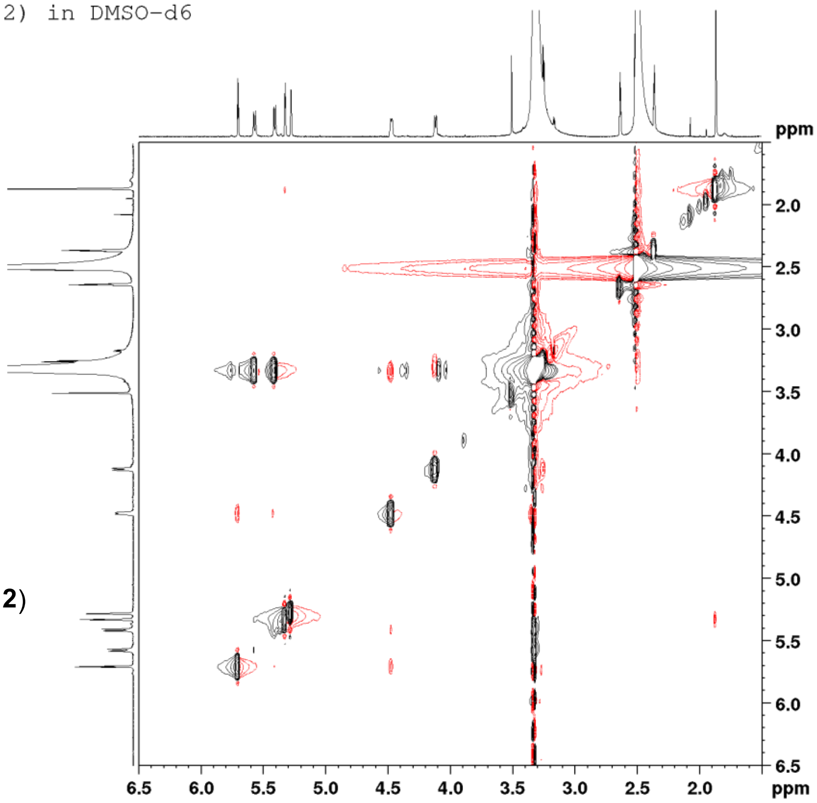

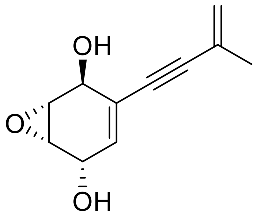

4-*epi*-(+)-asperpentyn (**12**)

Figure S36. NOESY spectrum (DMSO-d_6, 500 MHz) of 4-*epi*-(+)-asperpentyn (**12**).

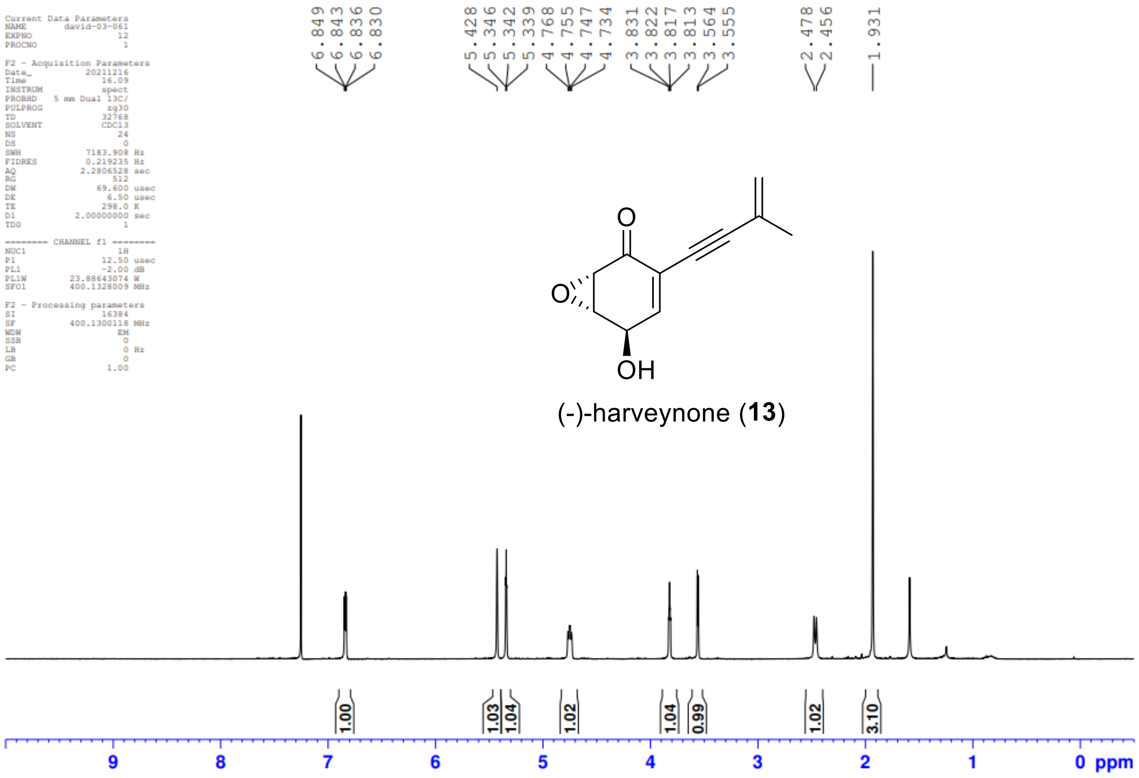

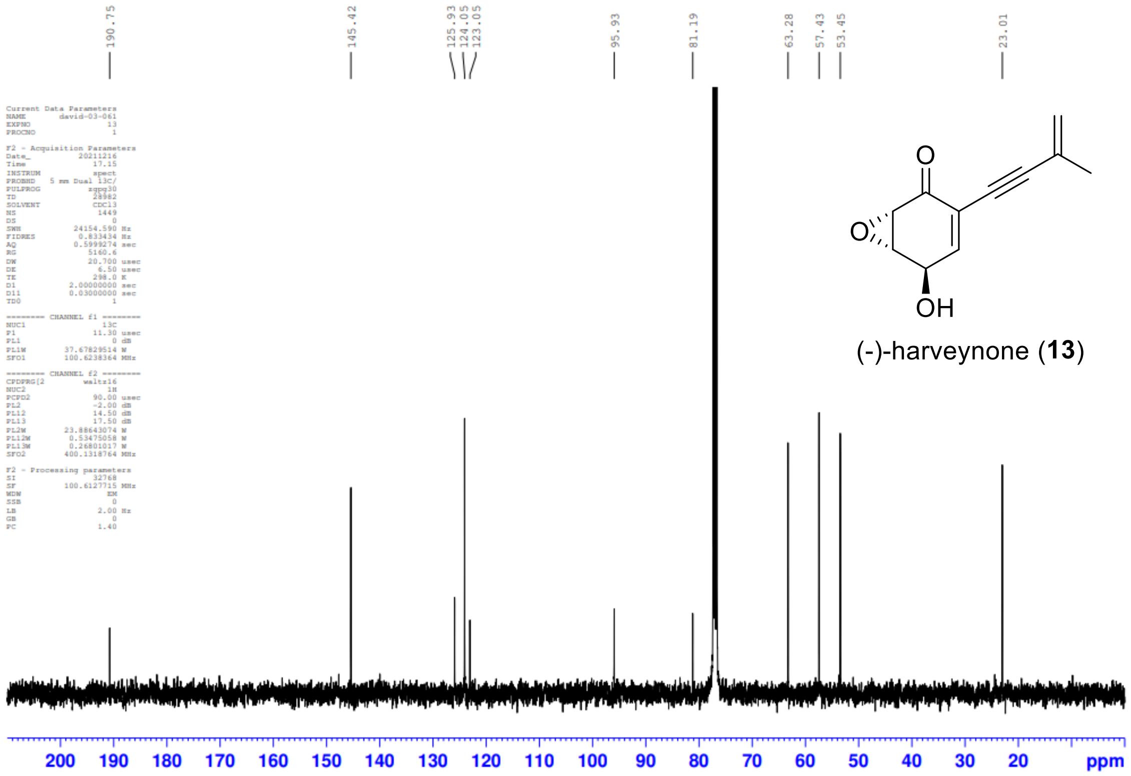

Figure S37. ^{1}H NMR spectrum ($CDCl_3$, 500 MHz) and ^{13}C NMR ($CDCl_3$, 125 MHz) of (-)-harveynone (**13**).

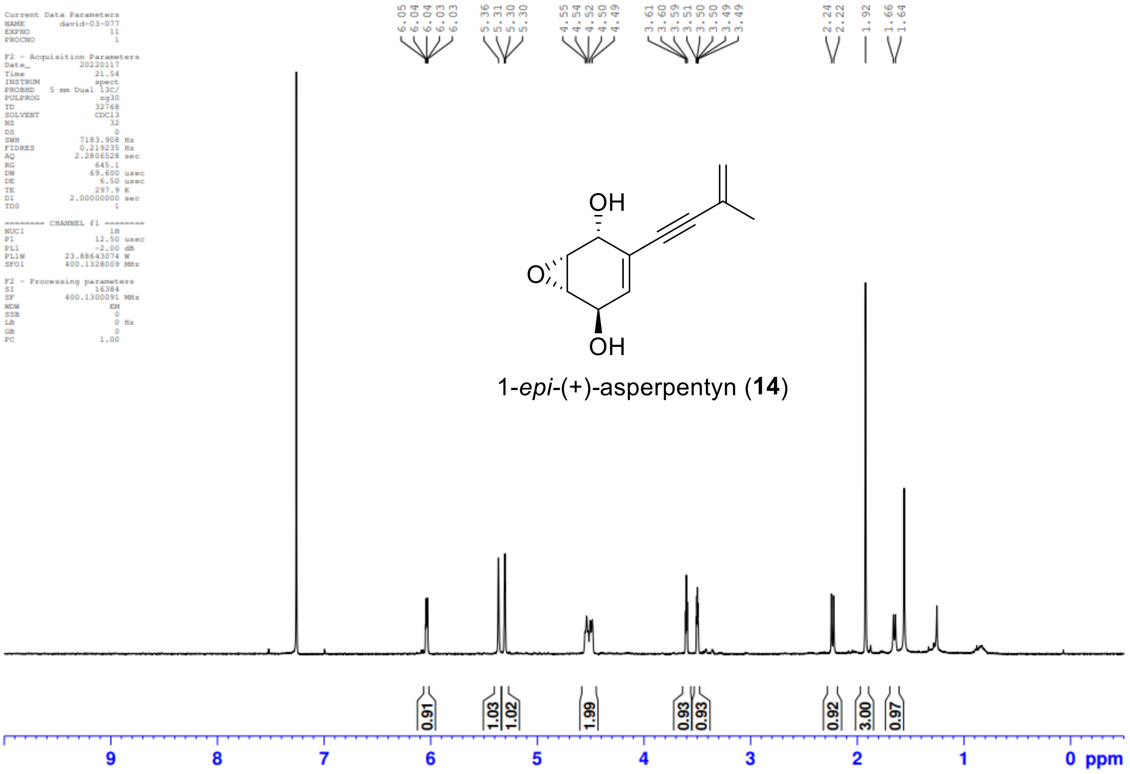

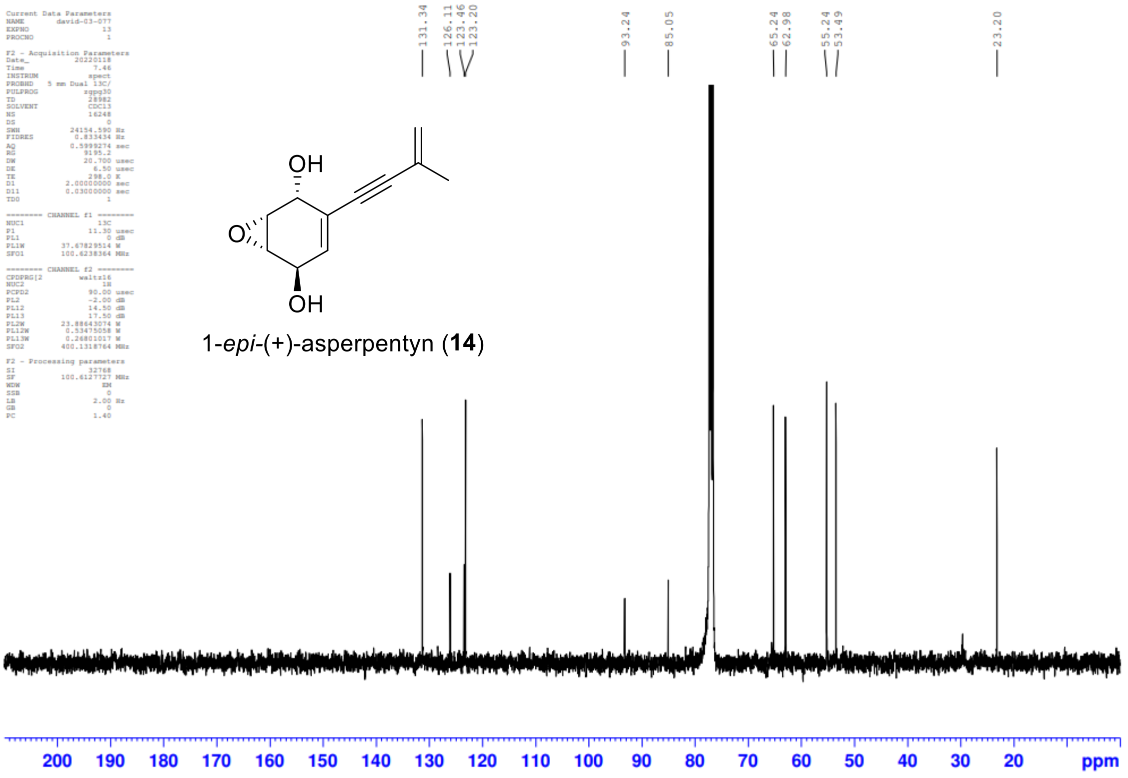

Figure S38. ^{1}H NMR spectrum ($CDCl_3$, 500 MHz) and ^{13}C NMR ($CDCl_3$, 125 MHz) of 1-*epi*-(+)-asperpenyn (**14**).

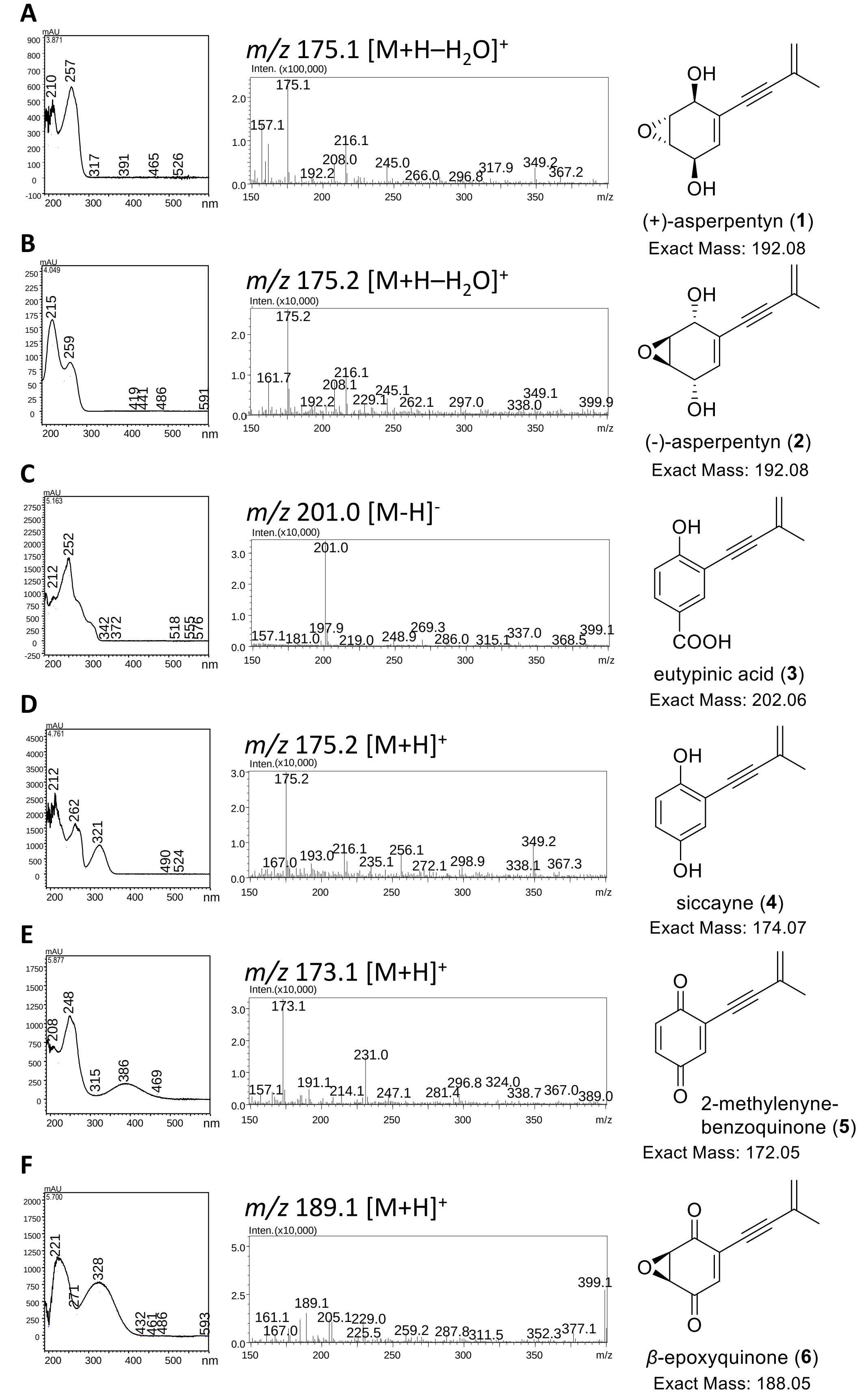

Figure S39. UV and MS spectra of **1**–**6**.

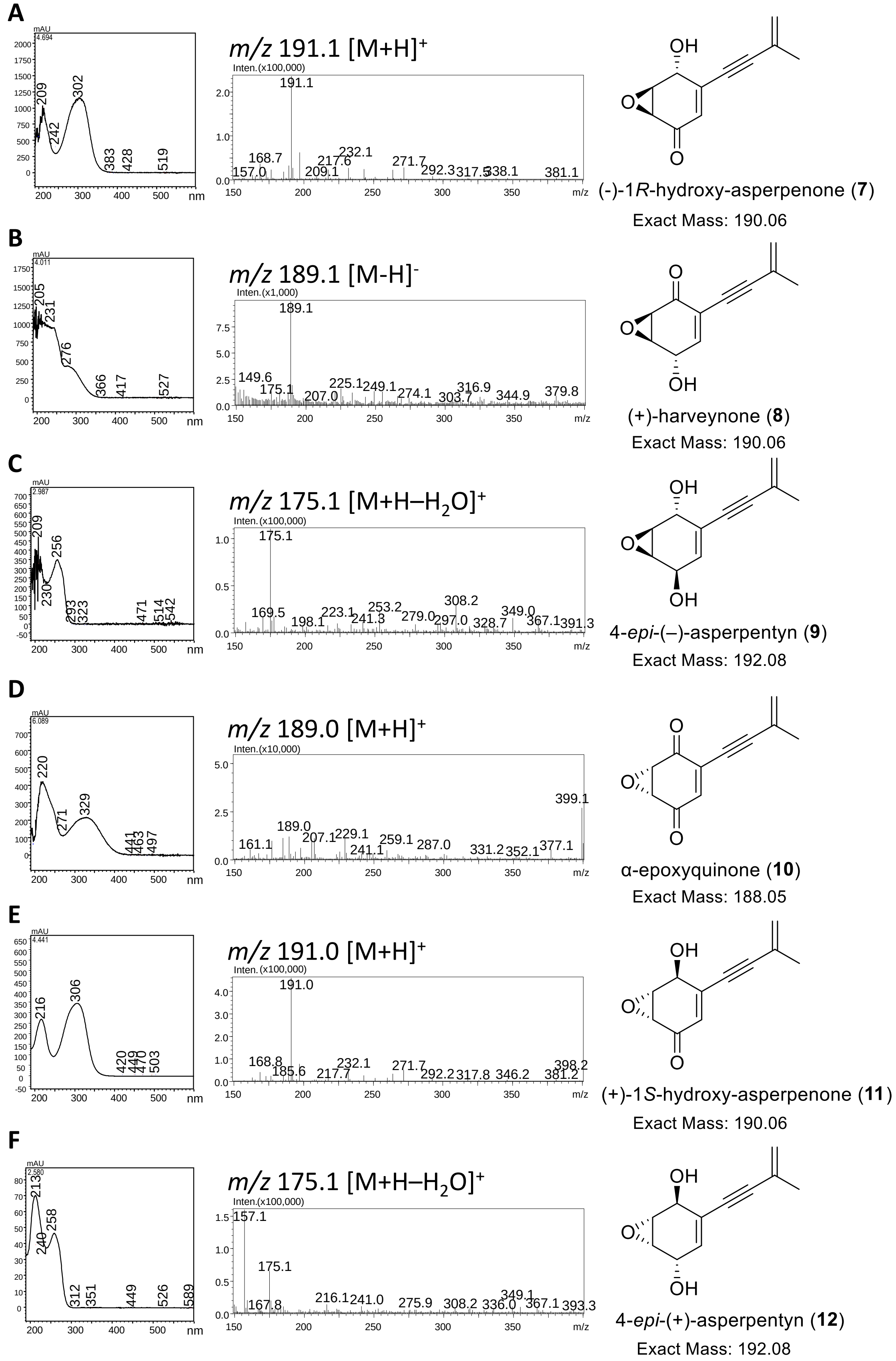

Figure S40. UV and MS spectra of **7**–**12**.

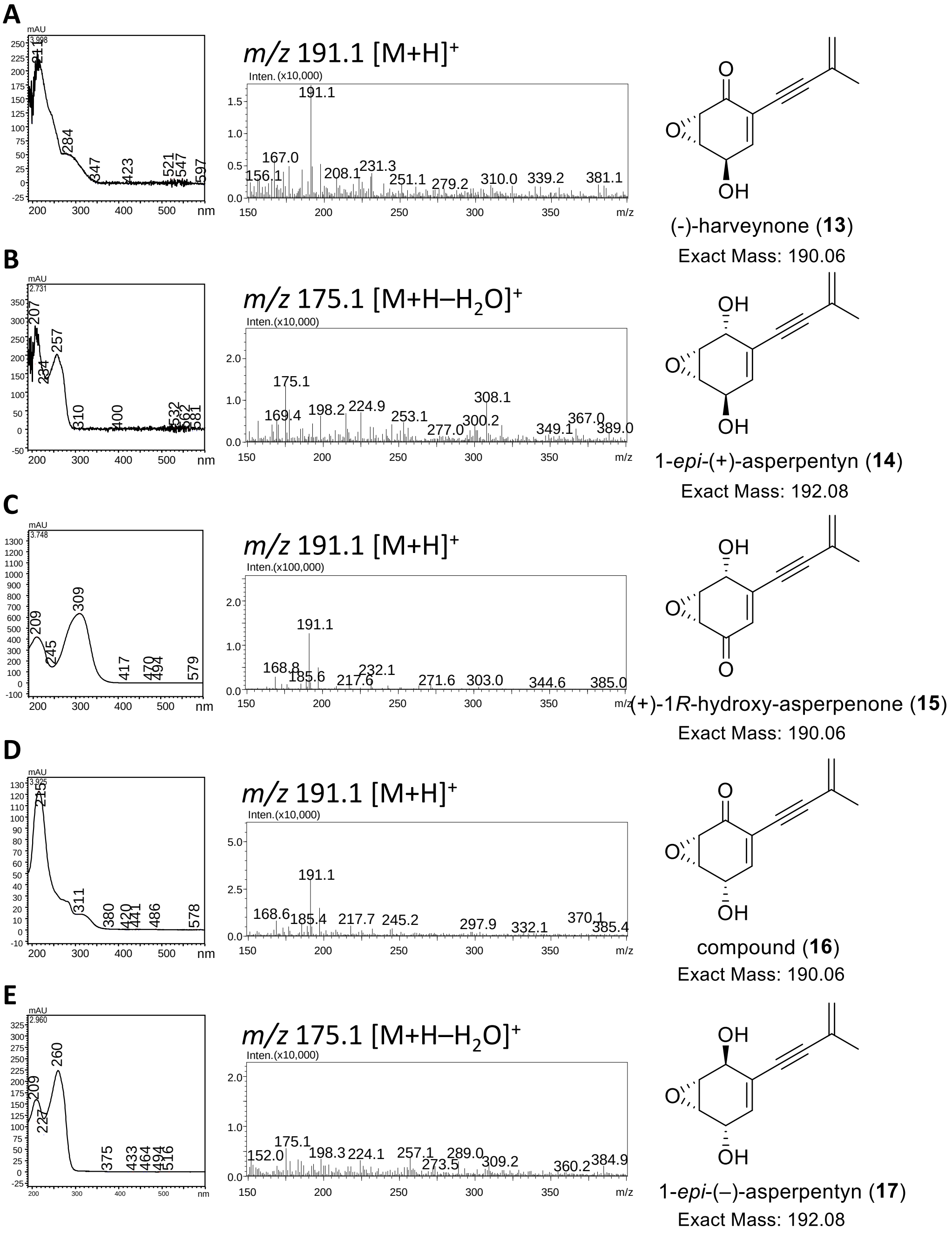

Figure S41. UV and MS spectra of **13**–**17**.

4-*epi*-(+)-asperpentyn (**12**), HRMS (EI) m/z: $[M]^+$ Calcd for $C_{11}H_{12}O_3$ 192.0786, Found 192.0782

[Mass Spectrum]
Data : 20230425_compound H-HR-001 Date : 25-Apr-2023 16:37
Sample : compound H
Note : 70eV
Ion Mode : EI+
RT : 0.10 min Scan# : 2
Elements : C 1000/0, H 1000/0, O 3/3
Mass Tolerance : 50mmu
Unsaturation (U.S.) : -0.5 - 1000.0

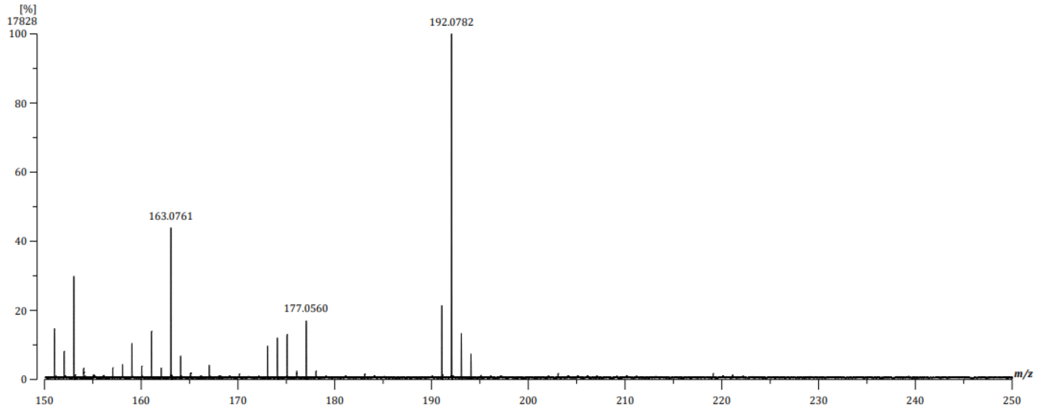

	Observed m/z	Int%					
	192.0782	100.00					
	Estimated m/z	Err[ppm / mmu]		U.S.	C	H	O
1	192.0786	-2.3 /	-0.4	6.0	11	12	3

Figure S42. (+)-HRMS spectra of 4-*epi*-(+)-asperpentyn (**12**).

4. Supplementary references

1. Mizuguchi, H., et al., *Characterization and application to hot start PCR of neutralizing monoclonal antibodies against KOD DNA polymerase.* The journal of biochemistry, 1999. **126**(4): p. 762–768.
2. Abramson, J., et al., *Accurate structure prediction of biomolecular interactions with AlphaFold 3.* Nature, 2024. **630**(8016): p. 493–500.
3. Notredame, C., D.G. Higgins, and J. Heringa, *T-Coffee: A novel method for fast and accurate multiple sequence alignment.* Journal of molecular biology, 2000. **302**(1): p. 205–217.
4. Hookins, D. and R. Taylor, *A double oxidation procedure for the preparation of halogen-substituted para-benzoquinone monoketals: asymmetric synthesis of (-)-harveynone.* Tetrahedron Letters, 2010. **51**(50): p. 6619–6621.
5. *WO2009066321 (A2) - Process for optically active sulfoxide compounds.*
6. Stille, J. and J. Simpson, *Stereospecific palladium-catalyzed coupling reactions of vinyl iodides with acetylenic tin reagents.* Journal of the American Chemical Society, 1987. **109**(7): p. 2138–2152.
7. Grünenfelder, D., et al., *Enantioselective synthesis of (-)-10-hydroxyacutuminine.* Angewandte Chemie-International Edition, 2022. **61**(16).
8. Man, Y., et al., *Total synthesis and structural revision of an isopanepoxydone analog isolated from Lentinus strigellus.* Organic & Biomolecular Chemistry, 2018. **16**(27): p. 5043–5049.
